# Comparative effectiveness of non-pharmacological interventions for social anxiety disorder in adults: a systematic review and meta-analysis

**DOI:** 10.3389/fpsyg.2026.1787151

**Published:** 2026-04-21

**Authors:** Yiman Xiong, Ningkun Xiao, Xianming Ding, Jiaye Zhang, Haoran Luo

**Affiliations:** 1 School of Leisure Sports, Chengdu Sport University, Chengdu, China; 2 Department of Immunochemistry, Institution of Chemical Engineering, Ural Federal University, Yekaterinburg, Russia; 3 Laboratory for Brain and Neurocognitive Development, Department of Psychology, Institution of Humanities, Ural Federal University, Yekaterinburg, Russia; 4 College of Sports Science, Jishou University, Jishou, China; 5 School of Economics and Management, Chengdu Sport University, Chengdu, China; 6 Institute of Physical Education, Sports and Tourism, Peter the Great St. Petersburg Polytechnic University, St. Petersburg, Russia

**Keywords:** adults, network meta-analysis, non-pharmacological interventions, social anxiety, systematic review

## Abstract

**Background:**

Social anxiety disorder (SAD) is a prevalent and disabling mental health condition in adults, associated with marked functional impairment, reduced quality of life, and substantial personal and societal burden. Previous meta-analyses have mainly evaluated single non-pharmacological interventions for SAD, but few evidence syntheses have compared the relative effectiveness of multiple non-pharmacological approaches within a unified analytic framework. As a result, the comparative efficacy of these interventions remains unclear.

**Methods:**

We searched the Cochrane Library, EBSCO, PubMed, Web of Science, and Embase from inception to 3 November 2025 for randomized controlled trials (RCTs) of non-pharmacological interventions for adults with SAD or clinically relevant social anxiety symptoms. Risk of bias was assessed using the Cochrane RoB 2 tool, and certainty of evidence was evaluated using GRADE. A frequentist random-effects network meta-analysis was conducted, and results were expressed as standardized mean differences (SMDs) with 95% confidence intervals (CIs).

**Results:**

A total of 104 RCTs involving 10,708 participants were included. Compared with control conditions, most non-pharmacological interventions were associated with significant reductions in social anxiety severity. Based on SUCRA values, cognitive behavioral therapy (CBT; 86.4%; SMD = −0.80, 95% CI: −0.92 to −0.68), combination therapy (CT; 71.7%; SMD = −0.74, 95% CI: −1.00 to −0.47), and psychotherapy (PT; 70.7%; SMD = −0.73, 95% CI: −0.92 to −0.54) ranked among the more effective interventions. Exploratory subgroup analyses suggested that CBT remained among the higher-ranked interventions across the country’s development level, intervention duration, baseline severity, and delivery format. However, the certainty of evidence was predominantly low to moderate, and subgroup findings should be interpreted cautiously.

**Conclusion:**

This network meta-analysis integrates direct and indirect evidence on non-pharmacological interventions for adult social anxiety. CBT, CT, and PT appear to be among the more effective approaches, although confidence in comparative estimates remains limited. These findings may inform shared decision-making and implementation planning, underscoring the need for well-designed head-to-head trials and more consistent reporting to strengthen comparative conclusions.

**Systematic review registration:**

PROSPERO (CRD420251179037).

## 1 Introduction

Social anxiety disorder (SAD) is a common mental disorder characterized by intense fear of negative evaluation and avoidance of social situations, often resulting in substantial impairment in daily functioning and quality of life (American Psychiatric Association, 2013). Epidemiological evidence indicates that the lifetime prevalence of SAD ranges from 4.0 to 18.7% (Stein et al., 2017; Van Ameringen et al., 2003). SAD typically begins during adolescence and frequently persists into adulthood (National Institute of Mental Health (NIMH), 2022). It has been associated with broad and multidimensional adverse consequences, including impaired social functioning (Voncken et al., 2008), interpersonal and emotional difficulties (Shechter Strulov and Aderka, 2025), and poorer academic and occupational outcomes (Alnemr et al., 2024; Gan et al., 2023). In addition, SAD is often accompanied by depression, irritability, substance misuse, and elevated suicide risk (McClannahan et al., 2025; Ohayon and Schatzberg, 2010; Patel et al., 2024; Wei et al., 2025).

Pharmacological treatment remains one of the main therapeutic approaches for SAD. However, its use must be considered alongside issues of tolerability, withdrawal symptoms, and safety in specific populations (Mavranezouli et al., 2015). Prior research has documented a range of medication-related adverse effects in patients with social anxiety, including metabolic abnormalities such as elevated total cholesterol (Pillinger et al., 2025), gastrointestinal and cardiovascular symptoms (Farhat et al., 2024), and potential increases in suicide risk (Khan and Bernadt, 2011). Given these limitations, together with barriers related to patient preference and treatment accessibility, non-pharmacological interventions have attracted increasing clinical and research attention.

Non-pharmacological interventions for SAD primarily include psychological, behavioral, and lifestyle-based approaches that do not rely on medication, and their therapeutic value has been supported by accumulating clinical evidence (Zaider and Heimberg, 2003). Regular physical exercise may reduce anxiety and depressive symptoms and improve overall mental health through both neurobiological and social-psychological pathways (Holland et al., 2024). Cognitive behavioral therapy (CBT), one of the most widely used interventions in mental health care, has demonstrated effectiveness in alleviating social anxiety, likely by modifying maladaptive cognitions and safety behaviors while encouraging gradual exposure to feared social situations (Manning et al., 2017). Virtual reality exposure therapy (VRET) delivers exposure-based treatment through immersive and controllable scenarios, enabling repeated confrontation with fear-related cues in a relatively safe environment and thereby reducing avoidance and improving functioning (Anderson et al., 2013). Social skills training can improve deficits in interpersonal competence through structured practice, feedback, and behavioral exercises and may be particularly helpful for patients with pronounced social performance difficulties (Beidel et al., 2014). Acceptance and commitment therapy (ACT) may also be beneficial by enhancing psychological flexibility and reducing experiential avoidance through strategies such as acceptance and cognitive defusion, while encouraging value-consistent social engagement (Hayes, 2016). Overall, these interventions appear to offer good accessibility and acceptability while avoiding the adverse effects associated with medication, and they have become an increasingly important component of SAD management (Purebl et al., 2023).

Despite growing interest in these approaches, the comparative evidence base remains limited. Most previous meta-analyses have focused on the efficacy of individual non-pharmacological interventions rather than systematically comparing multiple alternatives within a single analytic framework (Weng et al., 2024). Conventional pairwise meta-analysis is also unable to integrate indirect evidence across multiple competing interventions (Luo et al., 2025), which limits its usefulness when clinicians and policymakers must choose among several options. In addition, the evidence for some non-pharmacological interventions remains fragmented, with small samples, variable methodological quality, and limited generalizability (Dworkin et al., 2007).

More broadly, previous research on SAD has largely relied on pairwise comparisons of single interventions and has lacked a comprehensive synthesis capable of ranking the relative effectiveness of multiple non-pharmacological approaches within a unified framework (Kindred et al., 2022; Scaini et al., 2016). Network meta-analysis (NMA) allows the simultaneous integration of direct and indirect evidence and enables quantitative comparison and ranking of multiple interventions (Chaimani et al., 2024). Accordingly, we conducted a systematic review and network meta-analysis to evaluate and compare the effectiveness of different non-pharmacological interventions for reducing core social anxiety symptoms in adults with SAD. This study aimed to clarify the comparative efficacy of these interventions, address the lack of head-to-head evidence, and inform more evidence-based clinical decision-making for adults with social anxiety disorder.

## 2 Methods

The study protocol was registered in PROSPERO (CRD420251179037). To guarantee methodological transparency and rigour, we adhered to the PRISMA 2020 and PRISMA-NMA guidelines (Salanti et al., 2014).

### 2.1 Inclusion criteria

We selected studies according to the eligibility criteria listed below:

1. All participants were adults (aged ≥18).
2. Interventions did not involve drugs or other active substances, such as cognitive behavioral therapy and exercise.
3. Studies assessed social anxiety levels using valid and reliable scales.
4. Randomized controlled trials, including parallel-group and cluster-randomized designs (cross-over trials were included only if data from the first treatment period were available or could be extracted to avoid carryover effects).

### 2.2 Exclusion criteria

We excluded studies that met any of the conditions listed below:

1. Participants outside the adult age range (aged <18).
2. Interventions or control measures involving drugs or other active substances.
3. Failure to assess social anxiety-related outcomes, or use of unvalidated social anxiety scales.
4. Non-randomized controlled trials (e.g., observational studies, case reports, quasi-experimental designs).

### 2.3 Research screening

We used EndNote 21.5 software to screen all the retrieved research. Specifically, after the elimination of duplicate literature through the software’s own removal of duplicates function and manual removal of duplicates, two researchers independently screened the literature titles and abstracts according to the pre-formulated inclusion and exclusion criteria. All literature that may meet the inclusion criteria was summarized and the full text was retrieved for full-text review. In addition, any differences arising in the literature screening process were discussed one by one through remote meetings with a third experienced researcher in order to reach consensus. For detailed retrieval strategies and results, please refer to the Supplementary S1.

### 2.4 Data extraction

Two researchers extracted information from the final incorporated literature based on the pre-set standardized data extraction table: (1) basic research information; (2) demographic characteristics of the research subjects; (3) information of the intervention group and control group; (4) outcome indicators.

To enhance comparability across trials, the primary outcome was defined as post-intervention social anxiety severity at the earliest post-treatment time point reported in each study. When multiple eligible social anxiety scales were reported within a study at the same time point, we applied a pre-specified hierarchy (LSAS > SIAS > SPIN > other validated scales) to select a single effect estimate per study per comparison. This hierarchy reflects the comprehensiveness of the clinical constructs measured. The LSAS was prioritized as the gold standard, as it comprehensively assesses both fear and avoidance across distinct social interaction and performance situations (Heimberg et al., 1999). The SIAS was ranked second; while highly sensitive, it predominantly focuses on interaction anxiety rather than performance anxiety (Mattick and Clarke, 1998). The SPIN was ranked third, functioning more as a brief screening measure for a broad spectrum of symptoms (Connor et al., 2000). Other validated scales were only extracted when these primary measures were unavailable. Discrepancies were resolved by consensus after full-text verification.

### 2.5 Classification of interventions and rationale

We defined non-pharmacological interventions as structured strategies targeting psychological, behavioral, or lifestyle processes without the use of medication, consistent with previous conceptual frameworks (Kanzler et al., 2021; Liebich et al., 2024). Because intervention labels varied across trials, interventions were classified *a priori* according to their dominant therapeutic orientation and primary clinical target, rather than assuming full mechanistic equivalence among all interventions within a node.

The following categories were used: control conditions (CON), including waiting list, placebo/sham, treatment as usual, and routine care; virtual reality exposure-based therapy (VRET), including VR-delivered exposure-based programs; cognitive behavioral therapy (CBT), in which both cognitive and behavioral/exposure elements were central; acceptance and commitment therapy (ACT), delivered in person or online, including VR-assisted ACT when ACT remained the core therapeutic component; relaxation therapy (RT), defined as a broad relaxation-oriented node including mindfulness-based stress reduction, applied relaxation, and related relaxation-focused programs; social and interpersonal skills (SIS), including social efficacy therapy, cognitive social skills training, and other skills-focused programs centered on social performance and interpersonal competence; combination therapy (CT), including explicitly multi-component interventions in which no single active component could be considered dominant; psychotherapy (PT), including psychodynamic therapy, interpersonal psychotherapy, and related bona fide psychotherapies; attention training (AT), including attentional bias modification and interpretation bias modification; and reading therapy (READ), including guided self-help books/manuals and reading-based programs.

RT was retained as a broad class-level node for analytic feasibility and network connectivity. However, mindfulness-oriented approaches and somatic relaxation approaches may operate through partly distinct pathways; therefore, the RT estimate should be interpreted as a class-level estimate for relaxation-oriented approaches rather than as evidence that these interventions are interchangeable.

To evaluate the plausibility of the transitivity assumption, we summarized the distribution of prespecified effect modifiers across direct comparisons in the network, including baseline severity, diagnostic method, delivery format, number of sessions, and therapist qualification. Detailed definitions and clustering rules are provided in Supplementary Table S3, and the transitivity assessment is summarized in Supplementary Table S5.

### 2.6 Risk of bias and quality assessment

The risk of bias for all included studies was evaluated independently by two reviewers. They utilized the Cochrane Risk of Bias 2.0 (RoB 2) tool for this assessment. This evaluation examined five key domains: (1) the process of randomisation; (2) deviations from the intended interventions; (3) missing outcome data; (4) measurement of the outcome; (5) selection of the reported results. We judged each domain as “low risk,” “some concerns,” or “high risk.” Following the RoB 2 guidelines, the overall risk status was determined using these criteria: (1) If any domain was rated as high risk, the overall classification was “high risk”; (2) If one or more domains raised some concerns but none were high risk, the study was labeled as “some concerns”; (3) The study was considered “low risk” only if all domains were rated as low risk. Any discrepancies between the reviewers were resolved through consultation with a third reviewer.

### 2.7 Data synthesis and statistical analysis

Network Meta-Analysis (NMA) can integrate direct evidence and indirect evidence, and compare the efficacy of multiple interventions (Shim et al., 2017). We used it to show the structural characteristics of the evidence network in this study by drawing a network structure diagram. Specifically, the nodes in the network evidence diagram represent different interventions, and the size of the node is positively correlated with the total sample size, that is, the larger the node, the larger the sample volume covered by the node. The connection between nodes represents the existence of direct comparative studies between the two interventions, and the thickness of the connection is directly related to the number of studies of direct comparison (Florez et al., 2024).

We synthesized continuous outcomes using standardized mean differences (SMDs) along with 95% confidence intervals (CIs). This method was chosen to accommodate the varying measurement scales across studies. The SMDs were derived from the reported means and standard deviations (SDs) of the intervention and comparator groups. In this context, a negative SMD signifies a greater reduction in social anxiety severity (indicating improvement) compared to the control. We defined statistical significance at an alpha level of 0.05 (Higgins et al., 2024). In cases where standard deviations were missing, we calculated them from standard errors, confidence intervals, or t-values, following the formulas provided in Supplementary S2.

We conducted a random-effects NMA within a frequentist framework using Stata 17.0. Multi-arm trials were modelled accounting for the correlation between effect estimates arising from shared comparators. Between-study heterogeneity was quantified using τ^2^.

Consistency was evaluated at both global and local levels. For global inconsistency, we used the design-by-treatment interaction model; for local inconsistency, we used loop-specific inconsistency (inconsistency factor, IF) and node-splitting approaches. An IF with a 95% CI including 0 and node-splitting *p* > 0.05 were interpreted as no material evidence of inconsistency for that loop/comparison (Higgins et al., 2012). When inconsistency was suggested, we explored potential sources using pre-specified subgroup analyses (e.g., country development level and intervention duration) and examined the distribution of effect modifiers to assess the plausibility of transitivity.

Interventions were ranked using the surface under the cumulative ranking curve (SUCRA), ranging from 0% (least effective) to 100% (most effective). SUCRA rankings were interpreted as probabilistic summaries rather than definitive clinical recommendations, particularly when evidence certainty was low or inconsistency/heterogeneity was present. Small-study effects and publication bias were assessed using comparison-adjusted funnel plots at the network level and, for pairwise comparisons including at least 10 studies, Egger’s regression tests with trim-and-fill analyses.

## 3 Results

### 3.1 Literature selection

**Figure 1:**
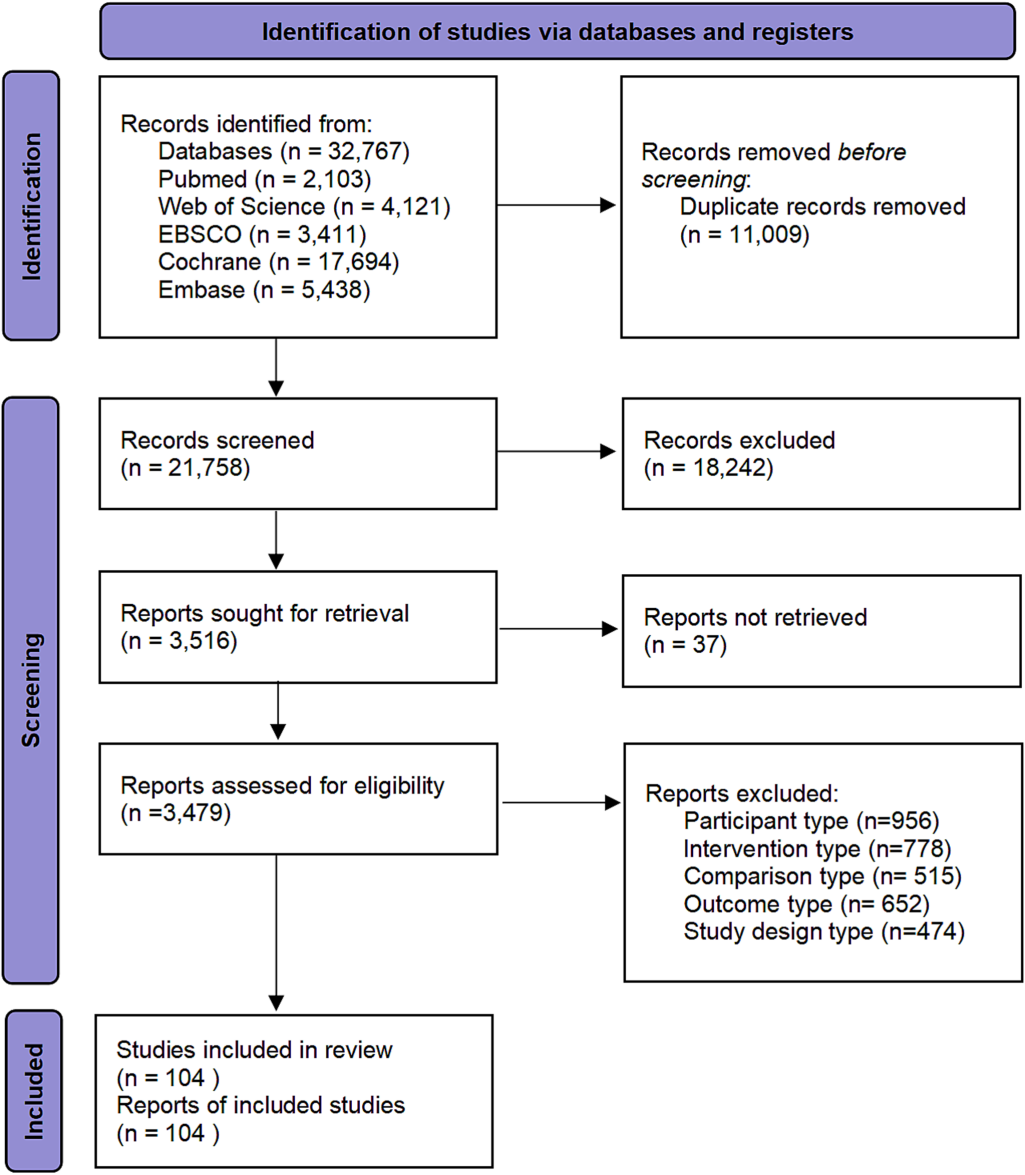
Flow diagram of systematic literature search.

This study retrieved a total of 32,767 potentially relevant publications through multi-database searches, specifically from PubMed (*n* = 2,103), Web of Science (*n* = 4,121), EBSCO (*n* = 3,411), Cochrane Library (*n* = 17,694), and Embase (*n* = 5,438). Automated deduplication was performed using the EndNote reference management tool, eliminating 11,009 duplicate records. Subsequently, 18,242 documents that did not meet the criteria were eliminated by reading titles and abstracts. In the end, 3,516 documents entered the full-text retrieval stage. 37 articles failed to be retrieved successfully due to the limitations of resource acquisition conditions. We reviewed the full text of 3,479 documents based on the pre-set inclusion and exclusion criteria, and finally determined that 104 studies met the inclusion criteria (see Figure 1; Supplementary S4).

### 3.2 Study characteristics

A total of 104 randomized controlled trials involving 10,708 participants from 27 countries or regions were included. The mean age ranged from 19.0 ± 1.4 years to 46.2 ± 10.8 years. Sample sizes ranged from 14 to 2,122 participants. Regarding sex distribution, 103 studies reported the sex ratio; 102 used mixed-sex samples, one included only females, and two did not report the proportion of male participants. Across the included trials, nine categories of non-pharmacological interventions were identified, with CBT being the most frequently studied approach. The detailed clinical and methodological characteristics of the included studies are presented in Table 1.

**Table 1:** Main characteristics of the randomized controlled trials included in the network meta-analysis.

Num	Study	Country/region	Continent	% of men	Sample *N*	Age	Intervention	Duration	Scale
1	Bouchard et al. (2017)	CAN	NA	11.8	17	36.2 ± 14.9	Cognitive behavioral therapy + virtual reality exposure therapy	60 min/1 time/14 wk	LSAS-SR
				22.7	22	36.7 ± 11.1	Cognitive behavioral therapy + reality exposure therapy	60 min/1 time/14 wk	
				45.0	20	30.6 ± 9.1	WL	–	
2	Gorinelli et al. (2023)	FIN	EU	29.7	37	24.0 ± 4.4	Virtual reality acceptance and commitment therapy	25 min/1 time/3 wk	SIAS
				30.8	39	25.8 ± 8.0	WL	–	
3	Olthuis et al. (2014)	CAN	NA	15.0	40	36.2 ± 12.2	Telephone-delivered cognitive behavioral therapy	50 min/1 time/8 wk	LSAS
				27.5	40	36.5 ± 10.4	WL	–	
4	Recabarren et al. (2019)	CHE	EU	9.4	32	21.2 ± 2.3	Group training integrating mindfulness, cognitive Behavioral Therapy, social skills and emotional regulation	120 min/1 time/8 wk	LSAS-SR
				15.6	32	21.5 ± 2.8	WL	–	
5	Piet et al. (2010)	DNK	EU	30.8	14	21.6 ± 2.8	Mindfulness-based cognitive therapy	120 min/1 time/8 wk	LSAS
					12	22.1 ± 2.5	Group cognitive-behavioral therapy	120 min/1 time/ 8wk	
6	Knijnik et al. (2004)	BRA	SA	66.7	15	34.5 ± 9.4	Psychodynamic group therapy	90 min/1 time/12 wk	LSAS
				66.7	15	35.1 ± 8.9	Credible placebo control	90 min/1 time/12 wk	
7	Amir and Taylor (2012)	USA	NA	17.4	23	28.9 ± 11.2	Interpretation modification programme	20 min/2 time/6 wk	LSAS
				38.5	26	33.1 ± 12.9	Interpretation control condition	20 min/2 time/6 wk	
8	Nordmo et al. (2015)	NO	EU	58.8	17	23.7 ± 3.4	Psychoeducation	4–6 h/1 time	SIAS
				54.5	20	27.3 ± 8.1	Guided internet-based cognitive behavioral therapy	4–6 h/1 time	
9	Berger et al. (2009)	CH	EU	41.9	31	28.1 ± 5.4	Internet-based cognitive behavioral therapy	Flex 10 wk	LSAS
				47.6	21	30.0 ± 5.1	WL	–	
10	Andersson et al. (2006)	SWE	EU	43.8	32	36.4 ± 9.4	Internet-based self-help cognitive-behavioral treatment	180 min/2 times/9 wk	LSAS-SR
				53.1	32	38.2 ± 11.0	WL	–	
11	Rapee et al. (2013)	AUS	OC	59.0	68	31.5 ± 10.2	Group cognitive behavioral therapy + attention bias modification programme group	120 min/1 time/16 wk +10 min/1 time/day	LSAS
				40.0	66	33.0 ± 10.3	Cognitive behavioral therapy	120 min/1 time/12 wk	
				49.4	77	31.1 ± 10.1	WL	–	
12	Blanco et al. (2010)	USA	NA	47.1	34	30.7 ± 8.0	Cognitive behavioral group therapy	150 min/1 time/12 wk	LSAS
				63.0	27		Placebo	–	
13	Handley et al. (2015)	AUS	OC	19.0	21	28.9 ± 8.3	Group cognitive behavioral therapy for perfectionism	120 min/1 time/8 wk	FNE-B
				19.0	21	33.0 ± 12.3	WL	–	
14	de Rutte et al. (2025)	USA	NA	29.4	54	37.7 ± 10.7	Game-based attention bias correction training	12 min/4 time/4 wk	LSAS-SR
				18.0	50	38.5 ± 10.0	Sham control	12 min/4 time/4 wk	
15	Gharraee et al. (2018)	IRN	AS	52.9	17	23.4 ± 4.6	Compassion focused therapy	60 min/1 time/12 wk	LSAS
				53.3	15	22.0 ± 4.4	WL	–	
16	Ezenwaji et al. (2021)	NG	AF	19.8	43	22.1 ± 3.0	Group rational emotive behavioral therapy	60 min/1 time/12 wk	SAS-A
				17.4	43	22.5 ± 3.0	AU	30 min/1 time/8 wk	
17	Bunnell et al. (2013)	USA	NA	46.7	15	24.2 ± 8.0	Attention training	NR/1 time/8 wk	LSAS-SR
				62.5	16	24.4 ± 7.0	CON	NR/1 time/8 wk	
18	Krafft et al. (2020)	USA	NA	23.1	52	20.4 ± 2.91	Acceptance and commitment therapy	NR/1 time/8 wk	LSAS
				22.0	50	20.6 ± 4.6	Traditional cognitive-behavioral therapy	NR/1 time/8 wk	
19	Amir et al. (2009)	USA	NA	36.4	22	27.6 ± 8.3	Attention modification programme	20 min/2 time/4 wk	LSAS
				45.5	26	31.1 ± 13.2	CON	20 min/2 time/4 wk	
20	Vally et al. (2024)	UAE	AS	31.3	64	20.7 ± 1.5	Acceptance and commitment therapy	30 min/1 time/4 wk	SIAS
				29.2	65	20.7 ± 1.6	WL	–	
21	Lazarov et al. (2018)	ISR	AS	64.0	25	33.6 ± 5.1	Cognitive behavioral group therapy + attention bias modification	90 min/1 time/18 wk +15 min/1 time/18 wk	LSAS
				64.0	25	35.5 ± 10.6	Cognitive behavioral group therapy	15 min/1 time/18 wk	
22	Bjornsson et al. (2011)	USA	NA	54.5	22	20.3 ± 1.9	Cognitive behavioral group therapy	120 min/1 time/8 wk	LSAS
				52.2	23	19.4 ± 1.1	Group psycho therapy	120 min/1 time/8 wk	
23	Mueller and Cougle (2023)	USA	NA	22.2	27	19.0 ± 1.4	Emotional writing + social skills training + exposure therapy	10 min/2 time/1 wk	SPIN
				7.4	27	19.9 ± 2.8	WL	–	
24	Clark et al. (2003)	UK	EU	48.0	20	33.2 ± 8.1	Cognitive therapy	75 min/1 time/16 wk	LSAS
					20		Self-exposure	30–40 min/1 time/16 wk	
25	D'El Rey et al. (2008)	BR	SA	60.0	15	33.0 ± 9.1	Cognitive behavioral group therapy	120 min/1 time/12 wk	SPIN
				50.0	16	34.9 ± 9.3	–	–	
26	Dagöö et al. (2014)	SWE	EU	51.9	27	34.7 ± 11.2	Mobile-based cognitive behavior therapy	NR/1 time/9 wk	LSAS-SR
				44.0	25	39.1 ± 11.3	Mobile-based interpersonal psycho therapy	NR/1 time/9 wk	
27	Cottraux et al. (2000)	FRA	EU	40.7	27	35.1 ± 10.7	Cognitive therapy +social skills training	60 min/1 time/6 wk +120 min/1 time/6 wk	FQ-SP
				42.9	28	32.8 ± 9.6	ST: supportive therapy	30 min/0.5 time/12 wk	
28	Heimberg et al. (1998)	USA	NA	44.4	36	37.0 ± 9.7	Cognitive behavioral group therapy	150 min/1 time/12 wk	FQ-SP
				45.6	33	34.0 ± 9.6	Educational support group therapy programme	150 min/1 time/12 wk	
				57.6	33	36.1 ± 10.2	Placebo	–	
29	Yoshinaga et al. (2016)	JPN	AS	61.9	21	32.5 ± 8.2	Cognitive behavioral therapy combined with standard care	50 min/1 time/16 wk	SPAI
				57.1	21	31.6 ± 9.2	UC	–	
30	Kählke et al. (2023)	DE	EU	38.0	100	27 ± 6.34	Unsupervised internet-based self-help intervention based on the cognitive behavioral therapy model	60 min/1 time/10 wk	LSAS
					100		WL	–	
31	Clark et al. (2006)	UK	EU	56.0	21	32.0 ± 8.6	Cognitive therapy	90 min/1 time/14 wk	LSAS
					21		Exposure therapy + applied relaxation therapy	90 min/1 time/14 wk	
					20		WL	–	
32	Boettcher et al. (2014)	SWE	EU	31.8	66	33.4 ± 10.4	Attention bias modification	NR/1 td/2 wk	LSAS-SR
				40.3	67		Internet-based cognitive-behavioral self-help	NR/1 td/2 wk	
33	Borgeat et al. (2009)	CH	EU	53.8	13	43.1 ± 9.9	Self-focused exposure therapy	150 min/1 time/8 wk	SADS
				50.0	14	37.1 ± 9.6	Standard cognitive-behavioral therapy	150 min/1 time/8 wk	
34	Powell et al. (2020)	UK	EU	17.7	1,061	37.4 ± 13.9	Self-help online intervention based on cognitive Behavioral Therapy principles	NR/NR/12 wk	SPIN-17
				20.1	1,061	36.9 ± 13.6	WL	–	
35	Abeditehrani et al. (2024)	IRN	AS	33.3	36	28.7 ± 7.8	Cognitive behavioral group therapy	150 min/1 time/12 wk	LSAS
				33.3	36	28.5 ± 8.4	Psychodrama	150 min/1 time/12 wk	
				33.3	36	27.6 ± 6.2	Cognitive behavioral psychodrama therapy	150 min/1 time/12 wk	
				33.3	36	27.5 ± 6.6	WL	–	
36	Bell et al. (2012)	NZ	OC	27.5	40	33.6 ± 10.9	Computerised cognitive behavioral therapy	NR/NR/12 wk	LSAS
				37.2	43	36.9 ± 12.0	WL	–	
37	Kählke et al. (2019)	DE	EU	38.0	100	26.7 ± 6.1	Unsupervised internet-based self-help intervention based on the cognitive behavioral therapy model	60 min/1 time/10 wk	LSAS
				27.0	100	26.7 ± 6.6	WL	–	
38	de Oliveira et al. (2012)	BRA	SA	29.4	17	33.9 ± 9.9	Trial-based thought record	60 min/12 times/16 wk 60 min/12 times/16 wk	LSAS
				21.1	19	34.9 ± 13.4	Conventional cognitive therapy		
39	Garcia-Lopez et al. (2006)	ESP	EU	29	15	20.8 ± 0.8	Adolescent cognitive behavioral group therapy	90 min/1 time/14 wk	SAS-A
					14		Adolescent social efficacy therapy	150 min/1 time/17 wk	
					15		School-based group sessions	90 min/1 time/12 wk	
40	Enock et al. (2014)	USA	NA	52.2	158	34.8 ± 11.4	Attention bias modification training	2.5 min/21 times/4 wk	LSAS-SR
					27		WL	–	
41	McCall et al. (2018)	CAN	NA	36.7	30	21.5 ± 4.1	Web-based cognitive behavioral therapy	NR/flex/4 month	SIAS
				20.0	35	22.1 ± 6.5	WL	–	
42	Mall et al. (2011)	DE	EU	58.3	12	25.5 ± 5.2	Cognitive model-based DVD self-help programme	NR/1 time/8 wk	SIAS
				25.0	12	26.1 ± 2.6	WL	–	
43	Mattick et al. (1989)	AUS	OC	27.3	11	42.7 ± 9.5	Cognitive restructuring without exposure	120 min/1 time/6 wk	SIAS
				36.4	11	41.7 ± 16.2	Guided exposure	120 min/1 time/6 wk	
				72.7	11	39.7 ± 9.3	Guided exposure + cognitive restructuring	120 min/1 time/6 wk	
				50.0	10	42.9 ± 15.1	WL	–	
44	Goldin et al. (2016)	USA	NA	44.4	36	34.1 ± 8.0	Cognitive-behavioral group therapy	150 min/1 time/12 wk	LSAS-SR
				44.4	36	29.9 ± 7.6	Mindfulness-based stress reduction	150 min/1 time/12 wk	
				44.4	36	34.1 ± 7.8	WL	–	
45	Furmark et al. (2009)	SWE	EU	34.5	29	34.9 ± 8.4	Internet-based cognitive behavioral therapy	NR/1 time/9 wk	LSAS-SR
				34.5	29	32.5 ± 8.5	Reading therapy	NR/1 time/9 wk	
				35.7	28	35.0 ± 10.4	Reading + online discussion	NR/1 time/9 wk	
				24.1	29	36.4 ± 9.8	Internet-based relaxation therapy	NR/1 time/9 wk	
46	Nilsson et al. (2012)	SWE	EU	42.9	7	36.4 ± 10.3	Imagery reimagining	60 min/1 time/1 wk	SIAS
				71.3	7	30.6 ± 15.5	Reading task	30–60 min/1 time/1 wk	
47	Hyett et al. (2018)	AUS	OC	43.1	17	35.2 ± 15.0	Imagery rescripting	90 min/1 time/1 wk	SIPS
					22		Verbal restructuring	90 min/1 time/1 wk	
					19		WL	–	
48	McEvoy et al. (2022)	AUS	OC	54.9	53	30.1 ± 12.8	Imagery-enhanced cognitive behavioral therapy	120 min/1 time/12 wk	SIAS
				46.3	54	27.1 ± 9.7	Verbal-directed cognitive behavioral therapy	120 min/1 time/12 wk	
49	Sigurðardóttir et al. (2022)	IS	EU	17.3	23	29.0	Online cognitive behavioral therapy	45 min/flex/16 wk	SIAS
					23		Online cognitive behavioral therapy + online progressive muscle relaxation	45 min/flex/16 wk	
					23		WL	–	
50	Johansson et al. (2017)	SWE	EU	50.0	36	42.9 ± 13.3	Internet-based emotion-focused psychodynamic therapy	10–15 min/1 time/10 wk	LSAS-SR
				27.8	36	42.9 ± 13.6	WL	–	
51	Carlbring et al. (2012)	SWE	EU	35.0	40	35.1 ± 13.3	Attention bias correction training	20 min/2 time/4 wk	LSAS-SR
				28.2	39	38.0 ± 12.0	Placebo attention training	20 min/2 time/4 wk	
52	Thew et al. (2022)	HKG	AS	31.8	22	34.5 ± 10.4	Therapist-guided online cognitive Therapy based on cognitive models	15–20 min/1–2 time/14 wk	LSAS-SR
				72.7	22	31.7 ± 8.4	WL	–	
53	Tulbure et al. (2015)	RO	EU	42.1	38	30.6 ± 8.0	Internet social phobia cognitive-behavioral therapy	NR/1 time/9 wk	LSAS-SR
				39.5	38	27.9 ± 7.8	WL	–	
54	Cougle et al. (2020a,b)	USA	NA	25.8	31	28.8 ± 10.7	Interpretation bias modification	25 min/2 time/4 wk	SPIN
				27.3	33	30.2 ± 12.4	Progressive muscle relaxation	25 min/2 time/4 wk	
55	Lin et al. (2019)	SG	AS	26.0	25	25.6	Exposure therapy based on arousal feedback	60 min/1 time/4 wk	LSAS
					25		WL	–	
56	Salaberría and Echeburúa (1998)	ESP	EU	52.0	24 24	31.0 ± 8.3	Self-exposure in vivo group self-exposure *in vivo* with cognitive Therapy	150 min/1 time/8 wk 150 min/1 time/8 wk	SAD
57	Furmark et al. (2009)	SWE	EU	22.5	40	35.0 ± 10.2	Internet-based cognitive behavioral therapy	60 min/1 time/10 wk	LSAS-SR
				40.0	40	37.7 ± 10.3	Reading therapy	60 min/1 time/10 wk	
				35.0	40	35.7 ± 10.9	WL	–	
58	Olivares-Olivares et al. (2016)	ESP	EU	38.7	31	19.9 ± 1.0	Social effectiveness therapy	120 min/1–2 time/12 wk	SPAI-SP
				40.0	30	20.0 ± 1.0	Cognitive behavioral group therapy	150 min/1 time/12 wk	
59	Bautista et al. (2022)	USA	NA	20.0	20	22.1 ± 6.0	Internet-based cognitive behavioral therapy + peer coaching support	NR/1 time/6 wk	SIAS-6
				40.0	15	21.5 ± 2.4	WL	–	
60	Kocovski et al. (2013)	CAN	NA	50.9	53	34.9 ± 12.5	Mindfulness and acceptance-based group therapy	120 min/1 time/12 wk	SPIN
				47.1	53	32.6 ± 9.0	Traditional cognitive behavioral group therapy	120 min/1 time/12 wk	
				35.4	31	36.5 ± 11.5	WL	–	
61	Kushner et al. (2013)	USA	NA	61.6	171	39.1 ± 9.7	Cognitive behavioral therapy	60 min/1 time/1.2 wk	SPS
				57.7	173	39.5 ± 10.6	Progressive muscle relaxation training	60 min/1 time/1.2 wk	
62	Leichsenring et al. (2013)	GER	EU	45.9	209	34.8 ± 12.0	Cognitive behavioral therapy	50 min/1 time/38.7 wk	LSAS
				46.3	207	34.3 ± 12.1	Psychodynamic therapy	50 min/1 time/37.4 wk	
				41.8	79	38.4 ± 12.3	WL	–	
63	Herbert et al. (2018)	USA	NA	43.7	49	29.9 ± 11.6	Acceptance and commitment therapy	60–90 min/1 time/10 wk	LSAS
				55.0	53	30.0 ± 10.2	Traditional cognitive behavioral group therapy	60–90 min/1 time/10 wk	
64	Schmidt et al. (2012)	USA	NA	31.6 23.1	57 39	37.5 ± 11.3 34.6 ± 9.9	False safety behavior elimination therapy WL	120 min/1 time/10 wk –	ASI
65	Zainal et al. (2021)	USA	NA	22.7	26	23.3 ± 9.3	Self-guided virtual reality exposure therapy	25–30 min/2 time/2 wk	SIAS
					18	23.3 ± 9.3	WL	–	
66	Alden and Taylor (2011)	CAN	NA	65.0	31	34.7	Integrated interpersonal cognitive-behavioral therapy	120 min/1 time/12 wk	SIAS
				52.0	25	33.2	WL	–	
67	Borge et al. (2008)	NOR	EU	47.5	40	37.7 ± 11.3	Residential cognitive therapy	45–90 min/1–4 time/10 wk	SPAI-SP
				50.0	40	37.2 ± 11.6	Residential interpersonal therapy	45–90 min/1–4 time/10 wk	
68	Rubin et al. (2022)	USA	NA	27.3	11	25.9 ± 15.4	Virtual reality exposure therapy + attention guidance training	45 min/1 time/1 wk	LSAS-SR
				50.0	10	19.2 ± 1.2	Standard virtual reality exposure therapy	45 min/1 time/1 wk	
69	Arai et al. (2023)	JPN	AS	NR	26	19.8 ± 0.8	Safety aid fading and eliminating intervention	120 min/1 time/1 wk	SIAS
				NR	33	19.8 ± 0.8	Health education and adaptive living	120 min/1 time/1 wk	
70	Koszycki et al. (2007)	CAN	NA	55.6	27	37.6 ± 11.1	Cognitive behavioral group therapy	150 min/1 time/12 wk	SPS
				38.5	26	38.9 ± 15.7	Mindfulness-based stress reduction	150 min/1 time/8 wk	
71	Koszycki et al. (2021)	CAN	NA	26.9	52	41.5 ± 13.6	Mindfulness-based interventions for social anxiety disorder	120 min/1 time/12 wk	LSAS
				48.9	45	40.0 ± 13.9	Cognitive behavioral group therapy	20 min/1 time/12 wk	
72	Schmidt et al. (2009)	USA	NA	66.7	18	22.0 ± 5.4	Attention training	20 min/2 time/4 wk	LSAS
				44.4	18	24.0 ± 12.1	PC	20 min/2 time/4 wk	
73	Teale Sapach and Carleton (2023)	CAN	NA	30.0	21	36.8 ± 13.5	Self-compassion training	70–110 min/1 time/6 wk	LSAS-SR
				42.1	21	32.2 ± 9.4	Applied relaxation training	60–90 min/1 time	
				25.0	21	33.9 ± 10.8	WL	–	
74	Abramowitz et al. (2009)	USA	NA	23.8	11	43.4 ± 10.8	Self-help cognitive behavioral therapy	15–30 min/5 times/8 wk	SIAS
					10	43.4 ± 10.8	WL	–	
75	Samantaray et al. (2021)	IND	AS	56.0	25	20.4 ± 1.9	Brief cognitive behavioral group therapy	120 min/1 time/6 wk	LSAS
				48.0	25	20.6 ± 1.8	Psychoeducational-supportive therapy	120 min/1 time/6 wk	
76	Singh and Samantaray (2022)	IND	AS	38.1	21	20.2 ± 0.8	Brief cognitive behavioral group therapy	140–150 min/1 time/6 wk	LSAS
				40.0	20	20.2 ± 1.0	Verbal exposure-enhanced cognitive behavioral therapy	140–150 min/1 time/6 wk	
77	Kan et al. (2025)	CHN	AS	12.0	31	22.3 ± 3.0	Smartphone-based self-administered virtual reality exposure therapy	25–29 min/1 time/2 wk	LSAS
				13.7	30	23.0 ± 2.8	WL	–	
78	Kocovski et al. (2019)	CAN	NA	26.3	93	23.9 ± 6.7	Mindfulness and acceptance-based self-help book	10 min/1 time/8 wk	LSAS
					59	23.9 ± 6.7	WL	–	
79	Titov et al. (2008a,b)	AUS	OC	44.0	50	37.5 ± 11.8	Clinician-assisted computerised cognitive behavioral therapy	35–60 min/1–2 time/10 wk	SIAS
				38.7	55	38.6 ± 12.6	WL	–	
80	Titov et al. (2008a,b)	AUS	OC	41.4	43	37.8 ± 10.7	Clinician-assisted computerised cognitive behavioral therapy	35–60 min/1–2 time/10 wk	SIAS
				32.5	45	35.7 ± 11.1	WL	–	
81	Cougle et al. (2020a,b)	USA	NA	14.6	48	33.0 ± 11.3	Safety behavioral reduction SMS intervention	5 min/1 time/2 days/4 wk	SPIN
				15.2	46	32.8 ± 11.8	Mindfulness-based present moment intervention	5 min/1 time/2 days/4 wk	
82	Stevenson et al. (2019)	AUS	OC	25.0	60	30.9 ± 12.3	Online self-compassion intervention	5–15 min/1 time/2 wk	SIAS
				22.0	59	27.1 ± 10.6	Online cognitive restructuring intervention	5–15 min/1 time/2 wk	
83	Khoramnia et al. (2020)	IND	AS	33.3	12	23.1 ± 1.0	Acceptance and commitment therapy	90 min/1 time/12 wk	SPIN
				25.0	12	21.1 ± 1.0	WL	–	
84	Stangier et al. (2011)	DEU	EU	55.3	38	34.6 ± 12.9	Cognitive therapy	60 min/1 time/20 wk	LSAS
				42.1	38	33.9 ± 9.5	Interpersonal psychotherapy	65 min/1 time/20 wk	
				36.6	41	38.1 ± 12.9	WL	–	
85	Maoz et al. (2013)	IL	AS	16.6	24	22.9 ± 1.9	Subthreshold attention bias correction training	10 min/2 time/2 wk	LSAS
				22.2	27	22.4 ± 1.3	Placebo attention task	10 min/2 time/2 wk	
86	Norton and Abbott (2016)	AUS	OC		20	20.8 ± 3.9	Imagery rescripting	40 min/1 time/1 wk	SIAS
				15.0	20	20.8 ± 3.9	Cognitive restructuring	40 min/1 time/1 wk	
					20	20.8 ± 3.9	Puzzle task	40 min/1 time/1 wk	
87	Morvaridi et al. (2018)	IRN	AS	0	12	23.8 ± 3.8	Group emotional schema therapy	120 min/1 time/10 wk	WATQ-SA
					12	24.0 ± 2.9	WL	–	
88	Beidel et al. (2014)	USA	NA	45.6	46	36.3 ± 13.9	Social effectiveness therapy	90 min/2 time/12 wk	SPAI
				56.1	41	36.3 ± 13.9	Exposure therapy	90 min/2 time/12 wk	
				36.8	19	36.3 ± 13.9	WL	–	
89	Jazaieri et al. (2018)	USA	NA	41.3	46	33.4 ± 7.6	Cognitive behavioral group therapy	150 min/1 time/12 wk	LSAS-SR
				42.5	40	32.7 ± 7.9	Group mindfulness-based stress reduction	150 min/1 time/12 wk	
90	Mersch (1995)	NLD	EU		12	35.6 ± 9.5	Exposure therapy	60 min/1 time/14 wk	PQ-SP
				67.6	12	35.6 ± 9.5	Integrated treatment	60 min/1 time/14 wk	
					10	35.6 ± 9.5	WL	–	
91	Andersson et al. (2012)	SWE	EU	22.5	102	38.1 ± 11.3	Internet-delivered cognitive behavioral therapy	Flex/1time/9 wk	LSAS-SR
				40.0	102	38.4 ± 10.9	Moderated online discussion forum	Flex/1 time/9 wk	
92	Titov et al. (2010)	AUS	OC	27.5	42	38.6 ± 12.0	Transdiagnostic internet-based cognitive behavioral therapy	Flex/1 time/8 time wk	SPSQ
				36.8	44	40.5 ± 14.1	WL	–	
93	Wen et al. (2024)	CHN	AS	35.0	80	28.1 ± 7.0	Conventional online cognitive behavioral therapy	120–180 min/1 time/8 wk	SIAS
				35.0	43	29.6 ± 8.3	WL	–	
94	Işık et al. (2025)	TUR	EU	32.8	58	19.7 ± 0.6	Motivational interviewing	60 min/1 time/5 wk	LSAS
				22.4	58	20.0 ± 0.8	AU	–	
95	Tillfors et al. (2008)	SWE	EU	16.7	19	30.4 ± 6.3	Online cognitive behavioral therapy + group exposure sessions	180 min/1 time/9 wk +135 min/1 time/5 wk	LSAS-SR
				21.1	19	32.3 ± 9.7	Online cognitive behavioral therapy	180 min/1 time/9 wk	
96	Rapee et al. (2007)	AUS	OC	41.0	56	36.5 ± 10.1	Pure self-help	Flex/1 time/12 wk	SIAS
				93.0	57	34.8 ± 10.1	Therapist-augmented self-help therapist	120 min/1 time/12 wk	
				49.0	59	34.8 ± 12.1	Standard group treatment	120 min/1 time/12 wk	
				56.0	52	36.2 ± 11.6	WL	–	
97	Carlbring et al. (2007)	SWE	EU	41.0	30	32.4 ± 9.1	Online cognitive behavioral therapy + weekly telephone support	Flex/1 time/9 wk	LSAS-SR
				29.0	30	32.9 ± 9.2	WL	–	
98	Levin et al. (2017)	USA	NA	34.0	40	20.5 ± 2.7	Web-based acceptance and commitment therapy	17 min/1–2 time/4 wk	CCAPS-34
					39		WL	–	
99	Himle et al. (2014)	USA	NA	69.0	29	44.9 ± 6.1	Work-related cognitive behavioral therapy	120 min/2 time/4 wk	LSAS
				65.5	29	42.2 ± 10.2	AU	–	
100	Himle et al. (2024)	USA	NA	60.8	120	46.2 ± 10.8	Work-related cognitive behavioral therapy + standard occupational services	120 min/2 time/4 wk	LSAS
				57.7	130	43.0 ± 10.9	AU	–	
101	Winter et al. (2025)	AUS	OC	27.0	39	40.5 ± 12.0	Video conference cognitive behavioral therapy	50 min/1 time/8 wk	SIAS-6
				41.0	39	37.7 ± 12.5	WL	–	
102	Safir et al. (2012)	ISR	AS	NR NR	28	27	Virtual reality cognitive behavioral therapy	60 min/1 time/12 wk	LSAS
					30		Cognitive behavior therapy	60 min/1 time/12 wk	
103	Chard et al. (2023)	UK	EU	69.2	13	32.0 ± 9.4	Virtual reality exposure therapy	20–30 min/1 time/3 wk	SPS
				58.3	12	39.0 ± 16.8	WL	–	
104	Ørskov et al. (2025)	DNK	EU	17.6	17	31.8 ± 11.4	Cognitive behavioral therapy based on virtual reality exposure	60 min/1 time/10 wk	SIAS
				31.1	16	36.8 ± 13.2	Cognitive behavioral therapy based on field exposure	60 min/1 time/10 wk	
				33.3	18	33.2 ± 9.8	Virtual reality relaxation therapy	15 min/0.5 time/10 wk	

Assessment of prespecified effect modifiers suggested that the network was broadly clinically coherent, although some imbalance across direct comparisons was evident. In particular, digital or self-help delivery formats were more frequently represented in READ and in a subset of CBT comparisons, whereas formally diagnosed SAD samples appeared more often in CBT-, PT-, and VRET-based comparisons. The number of sessions ranged from brief protocols to extended multi-week programs, and therapist qualifications varied from self-guided or no in-person therapist formats to trained psychologists or experienced therapists. These differences do not invalidate the network, but they support cautious interpretation of the transitivity assumption. Further details are provided in Supplementary Table S5.

### 3.3 Quality assessment

Two assessors evaluated risk of bias using RoB 2 across five domains. Given the nature of non-pharmacological interventions, blinding of participants and providers was frequently not feasible; this limitation was considered within the RoB 2 framework rather than being treated as an exclusion criterion. Overall, 17 studies were judged at low risk of bias, 58 raised some concerns, and 29 were judged at high risk.

Regarding the randomisation process, 104 studies described sequence generation and allocation concealment; 3 studies were rated as some concerns because they reported randomisation without sufficient methodological detail. For deviations from intended interventions, 13 studies did not clearly report the analytic approach (e.g., intention-to-treat vs. per-protocol), limiting assessment of bias related to non-adherence. For missing outcome data, 29 studies had high attrition (≥20%) and were judged at high risk. Most trials used psychometrically validated social anxiety scales; however, because outcomes were often self-reported and blinding was uncommon, judgements for outcome measurement and selective reporting were made conservatively when trial protocols/registrations were not available or were insufficiently described. Study-level RoB 2 judgements are provided in Supplementary S6.

### 3.4 Evidence network and consistency analysis

**Figure 2:**
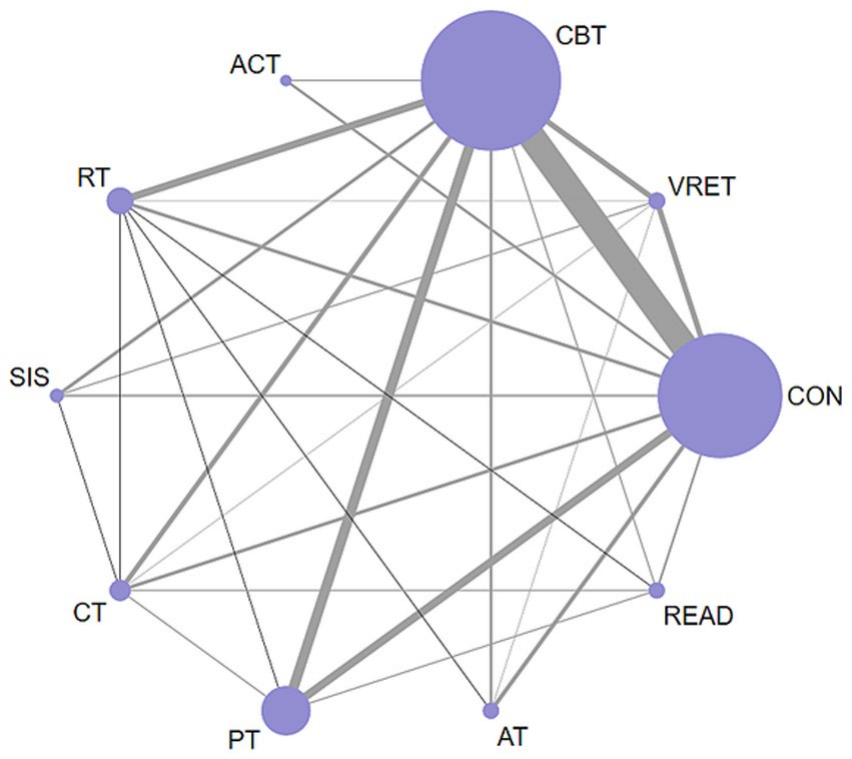
Network graph of eligible comparisons for non-pharmacological interventions in adults with social anxiety disorder.

Figure 2 illustrates the results of the network meta-analysis. In this diagram, each node stands for a specific intervention. The connecting lines represent direct comparisons between these interventions. Additionally, the thickness of each line corresponds to the number of studies involving that specific comparison.

Furthermore, we analyzed the network’s topological structure, evaluating internal consistency and inconsistency—particularly concerning closed-loop configurations. This involved conducting circular consistency tests, overall consistency tests, and local consistency tests. Results from these tests showed all *p*-values exceeded 0.05, indicating acceptable consistency in the findings. The common between-study variance for the overall network was estimated at *τ*^2^ = 0.12. Detailed outcomes of the inconsistency tests are presented in Supplementary S7.

### 3.5 The effect of non-pharmacological interventions on social anxiety in adults

**Figure 3:**
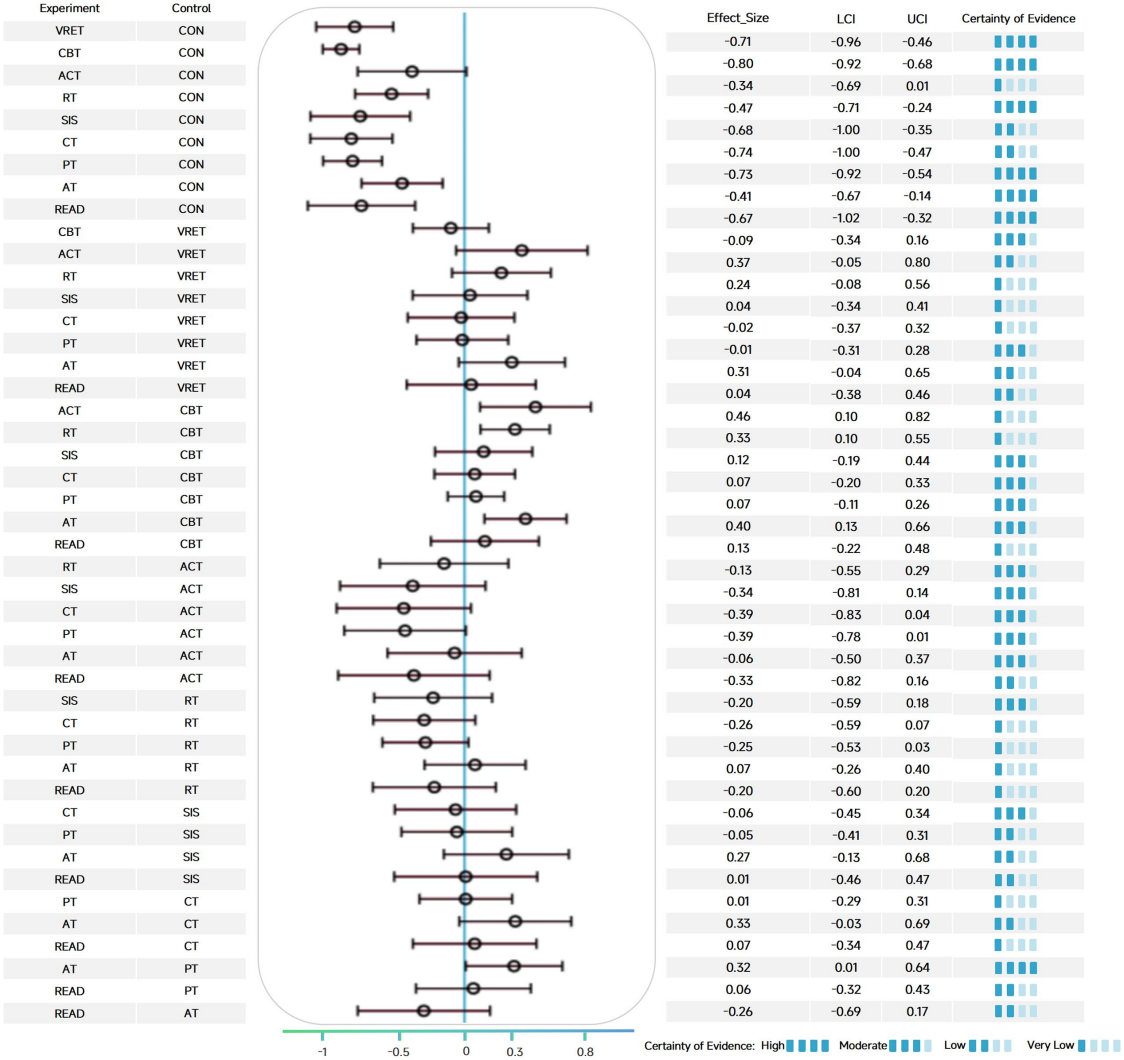
Forest plot and certainty evidence for social anxiety in adults.

This study, through a network meta-analysis of 104 randomized controlled trials, found that among the nine categories of non-pharmacological interventions assessed, most demonstrated significant potential in alleviating social anxiety levels in adults. Specifically, CBT (SMD = −0.80, 95% CI: −0.92 to −0.68), CT (SMD = −0.74, 95% CI: −1.00 to −0.47), PT (SMD = −0.73, 95% CI: −0.92 to −0.54), VRET (SMD = −0.71, 95% CI: −0.96 to −0.46), SIS (SMD = −0.68, 95% CI: −1.00 to −0.35), READ (SMD = −0.67, 95% CI: −1.02 to −0.32), RT (SMD = −0.47, 95% CI: −0.71 to −0.24), and AT (SMD = −0.41, 95% CI: −0.67 to −0.14) (Figure 3).

ACT did not reach statistical significance in the primary analysis (SMD = −0.34; 95% CI: −0.69 to 0.01), likely due to the relatively limited number of trials compared to other interventions.

Further head-to-head intergroup comparisons revealed that CBT exhibited significantly superior efficacy compared to ACT (SMD = −0.46, 95% CI: −0.82 to −0.10), AT (SMD = −0.40, 95% CI: −0.66 to −0.13), and RT (SMD = −0.33, 95% CI: −0.55 to −0.10). Additionally, PT demonstrated significantly greater efficacy than AT (SMD = −0.32, 95% CI: −0.64 to −0.01). The league table displaying the relative treatment effects for all pairwise comparisons is presented in Supplementary S8.

### 3.6 Possible ranking of effectiveness in reducing social anxiety in adults

**Figure 4:**
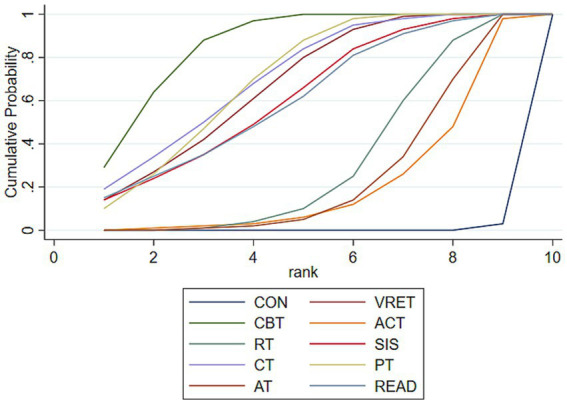
SUCRA probability sorting plot for adult social anxiety.

Probabilistic analysis based on the cumulative area under the ranking curve (SUCRA) showed that CBT had the highest SUCRA value (86.4%). The SUCRA values for all interventions, ranked from highest to lowest, are as follows: CBT (86.4%), CT (71.7%), PT (70.7%), VRET (68.2%), SIS (62.5%), READ (61.5%), RT (31.8%), AT (25.2%), and ACT (21.6%). These ranking results should be interpreted descriptively, because SUCRA values reflect both effect estimates and uncertainty and do not by themselves establish clear superiority when direct comparisons are not statistically significant (see Figure 4 for details). SUCRA scores and ranking results are detailed in Supplementary S9, S10.

### 3.7 Subgroup analysis

Given that intervention effects may differ according to key study or population characteristics, we performed pre-specified subgroup analyses to examine potential heterogeneity across relevant subgroups. Detailed graphical and tabular results for all subgroup analyses are provided in Supplementary S12.

#### 3.7.1 Subgroup analysis by country development level

Within the developed countries subgroup, CBT (SUCRA = 81.4%; SMD = −0.75; 95% CI:−0.86 to −0.63) > VRET (SUCRA = 77.5%; SMD = −0.74; 95% CI: −1.02 to −0.47) > CT (SUCRA = 71.7%; SMD = −0.71; 95% CI: −0.96 to −0.46) > PT (SUCRA = 68.2%; SMD = −0.69; 95% CI: −0.89 to −0.48), SIS (SUCRA = 63.0%; SMD = −0.65; 95% CI: −0.96 to −0.35), READ (SUCRA = 62.2%; SMD = −0.65; 95% CI:−0.97 to −0.32), RT (SUCRA = 31.0%; SMD = −0.44; 95% CI:−0.66 to −0.22) and AT (SUCRA = 22.2%; SMD = −0.35; 95% CI:−0.60 to−0.09) demonstrated significant superiority over control groups. Within the developing countries subgroup, CBT (SUCRA = 87.0%; SMD = −1.34; 95% CI: −1.93 to −0.75), PT (SUCRA = 53.8%; SMD = −0.98; 95% CI: −1.55 to −0.42), and VRET (SUCRA = 50.7%; SMD = −0.93; 95% CI: −1.68 to −0.17) demonstrated significant superiority over control groups. Across both country-development subgroups, CBT remained among the highest-ranked interventions.

#### 3.7.2 Subgroup analysis by intervention duration

Within the interventions lasting ≥ 8 weeks subgroup, AT (SUCRA = 75.9%; SMD = −0.99; 95% CI: −1.94 to −0.04) > VRET (SUCRA = 75.6%; SMD = −0.84; 95% CI: −1.15 to −0.53) > CT (SUCRA = 74.0%; SMD = −0.83; 95% CI: −1.11 to −0.55) > CBT (SUCRA = 71.0%; SMD = −0.79; 95% CI: −0.92 to −0.67) > READ (SUCRA = 57.9%; SMD = −0.72; 95% CI: −1.05 to −0.39) > SIS (SUCRA = 54.8%; SMD = −0.70; 95% CI: −1.01 to −0.40) > PT (SUCRA = 50.0%; SMD = −0.68; 95% CI: −0.89 to −0.46) > RT (SUCRA = 28.7%; SMD = −0.50; 95% CI: −0.74 to −0.25) demonstrated significant superiority over control groups. Within the interventions lasting < 8 weeks subgroup, CBT (SUCRA = 86.5%; SMD = −0.83; 95% CI: −1.12 to −0.54) > PT (SUCRA = 82.2%; SMD = −0.80; 95% CI: −1.20 to −0.40) > VRET (SUCRA = 55.4%; SMD = −0.53; 95% CI: −0.97 to −0.08) > ACT (SUCRA = 55.0%; SMD = −0.53; 95% CI: −1.02 to −0.03) > AT (SUCRA = 37.0%; SMD = −0.35; 95% CI: −0.70 to −0.01) demonstrated significant superiority over control groups. Ranking patterns differed by intervention duration, with AT ranking highest in the ≥ 8-week subgroup and CBT ranking highest in the < 8-week subgroup.

#### 3.7.3 Subgroup analysis by baseline severity

Because formal diagnostic status and baseline severity were strongly intertwined and inconsistently reported across trials, we used baseline severity as the most analyzable proxy for this source of clinical heterogeneity. A descriptive cross-tabulation of diagnostic status within each severity subgroup is provided in Supplementary Table S5. Formally diagnosed samples were concentrated predominantly in the Severe (65/71 studies) and Moderate to Severe (15/24 studies) strata, whereas screening-based samples were relatively more common in the milder strata. Because the Mild to Moderate subgroup contained only three studies, and the distribution of diagnostic approaches was sparse and uneven across severity strata, a separate diagnostic-status network analysis was not considered sufficiently robust. We successfully conducted full subgroup network meta-analyses for the remaining three subgroups. Within the subclinical severity subgroup, PT (SUCRA = 67.2%; SMD = −1.18; 95% CI: −2.19 to −0.18) > CBT (SUCRA = 56.8%; SMD = −1.05; 95% CI: −1.80 to −0.29) demonstrated significant superiority over control groups. Within the moderate to severe subgroup, CBT (SUCRA = 88.9%; SMD = −0.65; 95% CI: −0.81 to −0.49) > AT (SUCRA = 65.3%; SMD = −0.53; 95% CI: −0.81 to −0.25) > CT (SUCRA = 64.0%; SMD = −0.53; 95% CI: −0.98 to −0.08) > RT (SUCRA = 52.8%; SMD = −0.46; 95% CI: −0.75 to −0.17) > ACT (SUCRA = 47.1%; SMD = −0.42; 95% CI: −0.76 to −0.08) > PT (SUCRA = 43.5%; SMD = −0.39; 95% CI: −0.74 to −0.05) demonstrated significant superiority over control groups. Within the severe subgroup, CBT (SUCRA = 85.7%; SMD = −0.83; 95% CI: −0.98 to −0.68) > CT (SUCRA = 78.3%; SMD = −0.80; 95% CI: −1.11 to −0.49) > PT (SUCRA = 67.9%; SMD = −0.73; 95% CI: −0.96 to −0.51) > VRET (SUCRA = 66.2%; SMD = −0.72; 95% CI: −1.01 to −0.42) > SIS (SUCRA = 63.0%; SMD = −0.70; 95% CI: −1.03 to −0.36) > READ (SUCRA = 61.9%; SMD = −0.69; 95% CI: −1.05 to −0.32) > RT (SUCRA = 34.0%; SMD = −0.47; 95% CI: −0.75 to −0.18) > AT (SUCRA = 25.4%; SMD = −0.36; 95% CI: −0.68 to −0.03) demonstrated significant superiority over control groups. CBT and PT appear to be the most consistently effective interventions for reducing social anxiety across varying levels of baseline severity.

#### 3.7.4 Subgroup analysis by delivery format

Within the individual face-to-face format subgroup, CBT (SUCRA = 88.3%; SMD = −1.03; 95% CI: −1.27 to −0.80) > VRET (SUCRA = 80.5%; SMD = −0.97; 95% CI: −1.31 to −0.62) > AT (SUCRA = 79.6%; SMD = −1.00; 95% CI: −1.64 to −0.35) > PT (SUCRA = 62.8%; SMD = −0.77; 95% CI: −1.11 to −0.44) > SIS (SUCRA = 46.1%; SMD = −0.56; 95% CI: −1.01 to −0.12) demonstrated significant superiority over control groups. Within the group face-to-face format subgroup, CBT (SUCRA = 81.5%; SMD = −0.85; 95% CI: −1.15 to −0.55) > PT (SUCRA = 72.8%; SMD = −0.80; 95% CI: −1.19 to −0.42) > CT (SUCRA = 54.9%; SMD = −0.66; 95% CI: −1.21 to −0.11) > RT (SUCRA = 41.4%; SMD = −0.53; 95% CI: −1.05 to −0.02) demonstrated significant superiority over control groups. Within the online format subgroup, READ (SUCRA = 86.0%; SMD = −0.77; 95% CI: −1.21 to −0.32) > CBT (SUCRA = 84.2%; SMD = −0.70; 95% CI: −0.85 to −0.55) > CT (SUCRA = 63.8%; SMD = −0.55; 95% CI: −1.06 to −0.05) > PT (SUCRA = 56.1%; SMD = −0.48; 95% CI: −0.89 to −0.08) > ACT (SUCRA = 52.9%; SMD = −0.46; 95% CI: −0.84 to −0.07) > AT (SUCRA = 42.0%; SMD = −0.37; 95% CI: −0.63 to −0.12) demonstrated significant superiority over control groups. Within the mixed format subgroup, SIS (SUCRA = 81.5%; SMD = −0.86; 95% CI: −1.25 to −0.47) > CT (SUCRA = 76.1%; SMD = −0.81; 95% CI: −1.21 to −0.41) > VRET (SUCRA = 65.7%; SMD = −0.73; 95% CI: −1.08 to −0.38) > CBT (SUCRA = 63.3%; SMD = −0.72; 95% CI: −1.02 to −0.42) > READ (SUCRA = 44.3%; SMD = −0.53; 95% CI: −0.93 to −0.13) > PT (SUCRA = 41.1%; SMD = −0.50; 95% CI: −0.94 to −0.07) demonstrated significant superiority over control groups. Overall, CBT remained among the higher-ranked interventions across all delivery formats; however, the relative prominence of READ in online settings and SIS in mixed-format settings should be interpreted cautiously, as these subgroup patterns may reflect differences in evidence volume, comparator structure, and precision across format-specific networks.

## 4 Discussion

### 4.1 Principal findings and uncertainty

In this systematic review and network meta-analysis of 104 randomized controlled trials involving 10,708 adults across 27 countries and regions, most non-pharmacological interventions were associated with lower social anxiety severity than control conditions. Across the full network, cognitive behavioral therapy (CBT), combination therapy (CT), and psychotherapy (PT) ranked among the more effective approaches, while virtual reality exposure therapy (VRET), social and interpersonal skills training (SIS), reading therapy (READ), relaxation therapy (RT), and attention training (AT) also showed beneficial associations relative to control in the primary analysis.

However, these findings should not be interpreted as evidence that a single intervention is uniformly superior across all clinical contexts. Many head-to-head comparisons between active interventions were not statistically significant, confidence intervals often overlapped, and evidence certainty was frequently low. In addition, SUCRA values provide probabilistic rankings rather than definitive statements of clinical superiority. Accordingly, the overall pattern of evidence supports the conclusion that several non-pharmacological interventions appear beneficial, with CBT showing a relatively consistent pattern of benefit and high ranking across analyses.

### 4.2 Interpretation of CBT rankings and comparative efficacy

CBT ranked highly in the overall network and in several subgroup analyses. This pattern is clinically plausible, but it should be interpreted in the context of the broader evidence structure rather than as proof of large treatment differences. First, CBT has been studied more extensively than most other interventions. A larger evidence base can produce more precise estimates, which in turn can influence ranking probabilities. Second, CBT protocols are often manualized and supported by relatively well-established training and supervision procedures, which may improve treatment fidelity across trials. By contrast, reporting of therapist training, fidelity monitoring, and adherence is less consistent across several other intervention types, which may contribute to greater variability in estimated effects.

Third, the observed differences between active interventions were often modest. This may reflect the presence of overlapping therapeutic ingredients across approaches, such as exposure to feared situations, cognitive restructuring or reappraisal, social skills practice, and structured between-session exercises. It may also reflect trial design. Many studies compared active interventions with waitlist or usual care controls, which can yield larger effects than comparisons with credible active controls. When active treatments are compared directly, differences are often smaller and harder to detect unless studies are adequately powered and tightly controlled.

Fourth, intervention dose and implementation differed across trials. Some approaches may require longer practice to achieve stable symptom change, whereas others may show earlier effects. Variability in session number, treatment intensity, delivery format, and adherence may therefore attenuate apparent between-intervention differences. Finally, study populations also differed. Some trials enrolled formally diagnosed SAD samples, whereas others included participants with elevated symptoms identified through screening measures. Baseline severity, comorbidity, and treatment context may all influence both absolute improvement and comparative performance.

Taken together, the relatively favorable ranking of CBT likely reflects a combination of genuine therapeutic benefit, a larger and more mature evidence base, stronger treatment standardization, and study-design features. This interpretation favors caution over simple “best treatment” claims and supports treatment selection that is responsive to patient needs and local service capacity.

### 4.3 Subgroup findings as hypotheses

The subgroup analyses by baseline severity and delivery format offer potentially informative signals, but they should be interpreted as exploratory and hypothesis-generating. Because formal diagnostic status and baseline severity were strongly intertwined and inconsistently reported across trials, baseline severity was used as the most analyzable proxy rather than conducting a separate diagnostic-status network meta-analysis or meta-regression. This approach was driven by the marked imbalance in diagnostic approaches across severity strata and by sparse data in the milder subgroups. Accordingly, the baseline-severity analyses are best viewed as reflecting differences in overall clinical burden rather than a clean distinction between formally diagnosed SAD and screening-based social anxiety samples.

CBT remained highly ranked in both developed and developing settings. The larger estimated effect size in developing settings may reflect contextual differences rather than true effect modification. Trials may differ in baseline severity, access to concurrent care, control conditions, therapist training, delivery format, and adherence monitoring. In addition, smaller evidence bases and wider confidence intervals in some subgroup networks limit inference.

CBT ranked highly in shorter interventions, whereas AT ranked higher in longer-duration interventions. This pattern is plausible but should be interpreted cautiously. CBT targets maladaptive cognitions and avoidance behaviors that may shift within weeks (Wake et al., 2024; Staiger et al., 2014), whereas AT may depend more heavily on repeated practice (Badura-Brack et al., 2015; Nelson et al., 2015). However, the long-duration AT estimate was imprecise, and rankings in this subgroup were sensitive to sparse evidence.

A similar caution applies to the delivery-format analyses. Although online and mixed-format subgroup networks identified some interventions with favorable rankings, these findings should not be interpreted as definitive evidence of format-specific superiority. Rather, they suggest that delivery format may interact with intervention content, implementation conditions, and study design and therefore deserves more focused head-to-head evaluation. Overall, the subgroup analyses are useful for guiding future research and for informing provisional treatment planning, but they do not justify strong policy or superiority claims on the basis of the current evidence alone.

### 4.4 Implementation, clinical decision-making, and cautious theoretical considerations

The key practical question is how comparative evidence should inform clinical decision-making when several interventions appear beneficial and differences between active treatments are often small. Our findings support a stepped, preference-sensitive, and context-aware approach. CBT appears to be a reasonable first-line option for many adults because it shows a relatively consistent pattern of benefit across settings and ranks highly in the overall network. Available subgroup evidence also suggests that CBT can be delivered across multiple formats, including individual, group, and digital or blended models; however, these findings remain exploratory and should not be interpreted as proof that one delivery format is inherently superior or universally scalable.

CT and PT also ranked highly. CT may be especially relevant when the clinical goal is to address multiple maintenance factors simultaneously (Roy-Byrne, 2015), whereas PT may be particularly appropriate when interpersonal themes or relational distress are central to symptom presentation (Lübke et al., 2024; Altenstein et al., 2013). VRET may be useful when *in vivo* exposure is difficult to implement (Morina et al., 2023), and SIS may be relevant when social performance deficits are especially prominent (Qin et al., 2024). READ and RT may serve as more accessible or lower-intensity options in selected contexts (Fahy et al., 2023; Mészáros Crow et al., 2023).

Therefore, treatment selection should consider symptom severity, functional impairment, comorbidity, delivery feasibility, patient preference, and likely adherence, rather than relying on ranking metrics alone. Potential theoretical explanations for differences in relative performance remain speculative and should not be treated as findings of the present review. At most, the current pattern suggests that interventions ranking more favorably may offer broader or more structured ways of targeting maladaptive cognition, avoidance, interpersonal difficulties, or emotion regulation problems.

## 5 Certainty of evidence

We evaluated small-study effects and publication bias using comparison-adjusted funnel plots at the network level, supplemented by pairwise Egger’s regression tests and trim-and-fill analyses for comparisons including at least 10 studies. Comparison-adjusted funnel plots were used because they are appropriate for network meta-analysis and account for multiple treatment comparisons within the same evidence structure. Visual inspection suggested an overall broadly symmetrical distribution of study effects, although such inspection remains inherently subjective. Egger’s regression indicated potential small-study effects for CBT versus CON (*p* = 0.041) and RT versus CBT (*p* = 0.038), whereas no evidence of such effects was detected in the remaining tested comparisons. Trim-and-fill analyses did not impute additional studies in these comparisons. Taken together, these findings suggest that marked network-wide asymmetry was not apparent, but publication bias or small-study effects cannot be excluded for some pairwise contrasts. Detailed results are provided in Supplementary S11.

Using the GRADE framework, most direct comparisons were judged to provide very low to low certainty evidence, and no comparison reached high certainty. This pattern was driven primarily by two recurring limitations: risk of bias and imprecision. Many trials were affected by methodological limitations, including lack of blinding, incomplete reporting, or substantial attrition, and some direct and indirect estimates remained imprecise. As a result, the highest certainty level observed across the network was moderate. Detailed GRADE assessments for individual comparisons are provided in the Supplementary S13.

## 6 Limitations

Several limitations should be considered when interpreting these findings.

First, although publication bias and small-study effects were assessed using comparison-adjusted funnel plots and pairwise Egger’s regression tests rather than a single conventional funnel plot, reporting bias cannot be fully excluded. Potential small-study effects were identified in some comparisons, including CBT versus CON and RT versus CBT.

Second, most direct comparisons were rated as having low or very low certainty using GRADE, primarily because of risk of bias and imprecision. Many trials could not blind participants or therapists, and outcomes were often self-reported. Attrition was substantial in a subset of studies, and some trials did not clearly report analytic decisions related to adherence. These factors reduce confidence in the estimated effects.

Third, network estimates depend on the assumptions of transitivity and consistency. Although statistical tests did not identify material inconsistency, these tests may have limited power in sparse networks. Clinical and methodological heterogeneity was also considerable. Trials varied in diagnostic status, baseline severity, delivery format, treatment dose, and outcome measures. Because formal diagnostic status and baseline severity were strongly intertwined and inconsistently reported across trials, we used baseline severity as the most analyzable proxy in subgroup analyses rather than conducting a separate diagnostic-status network meta-analysis or meta-regression. This was a pragmatic methodological decision rather than a claim that the two constructs are equivalent. We also used standardized mean differences and the earliest post-intervention time point to improve comparability, but this approach may obscure clinically meaningful differences in timing and durability of response.

Fourth, SUCRA rankings are sensitive to network geometry and evidence precision. They should be interpreted as descriptive summaries of relative probability, not as definitive evidence that one intervention is clinically superior to all others.

Fifth, to preserve network connectivity and analytic feasibility, some interventions with overlapping clinical aims but partially distinct mechanisms were grouped into broader nodes. For example, the RT node combined mindfulness-based and somatic relaxation-oriented approaches. Although these interventions share a relaxation-oriented focus, they are not interchangeable and may affect symptoms through different pathways. Because we did not perform component-level sensitivity analyses separating these subtypes, the RT estimate should be interpreted as a broad class-level effect rather than as a precise estimate for any single relaxation modality.

## 7 Conclusion

In conclusion, several non-pharmacological interventions appear beneficial for reducing social anxiety in adults. CBT showed a relatively consistent pattern of benefit and high ranking across analyses, while CT and PT also ranked among the more favorable approaches. However, differences between active interventions were often modest, and confidence in comparative estimates was frequently limited by low to moderate certainty of evidence. These findings may inform shared decision-making, stepped care, and implementation planning, while also highlighting the need for stronger head-to-head trials, more consistent reporting, and more implementation-oriented comparative research.

## Data Availability

The original contributions presented in the study are included in the article/Supplementary material, further inquiries can be directed to the corresponding author.
