# Supplementary material for: Comparative effectiveness of non-pharmacological interventions for social anxiety disorder in adults: a systematic review and meta-analysis
Source: Front Psychol. 2026 Apr 21;17:1787151. doi: 10.3389/fpsyg.2026.1787151 (PMC13138971; doi:10.3389/fpsyg.2026.1787151)
Supplement: Supplementary file 1 [file Table_1.DOCX]

***Supplementary Material***

***Table of Contents***

**Supplementary S1 –** Database search strategy

**Supplementary S1.1 –** Database search strategy - Pubmed

**Supplementary S1.2 –** Database search strategy - Web of Science

**Supplementary S1.3 –** Database search strategy - EBSCO

**Supplementary S1.4 –** Database search strategy - Cochrane

**Supplementary S1.5 –** Database search strategy - Embase

**Supplementary** **S2 –** Conversion formula for standard deviation (SD)

**Supplementary S3 –** Definitions and Classification of Interventions and Controls

**Supplementary S4 –** Inclusion in the list of studies

**Supplementary S5 –** Summary of Key Effect Modifiers in the Network Meta-Analysis

**Supplementary S6 –** Risk of bias table of included studies

**Supplementary S7 –** Inconsistency Test

**Supplementary S7.1 –** Loop Inconsistency Test

**Supplementary S7.2 –** Global Inconsistency Test

**Supplementary S7.3 –** Local Inconsistency Test

**Supplementary S8 –** League table

**Supplementary S9** – Surface Under the Cumulative Ranking curve Score and Ranking

**Supplementary S10 –** **Ranking Probability for Social anxiety**

**Supplementary S11** – Assessment of Publication Bias and Small-Study Effects

**Supplementary S11.1 –** Comparison-adjusted funnel plot

**Supplementary S11.2** – Egger's regression test and trim-and-fill analysis results for eligible comparisons

**Supplementary S12 –** **Subgroup analysis**

**Supplementary S12.1 –** **Network evidence graph for developed country**

**Supplementary S12.2 –** **Network evidence graph for developing country**

**Supplementary S12.3 –** **Network evidence graph for Intervention duration of ≥ 8 weeks**

**Supplementary S12.4 –** **Network evidence graph for Intervention duration of ＜ 8 weeks**

**Supplementary S12.5 –** Sensitivity analysis for **developed country**

**Supplementary S12.6 –** Sensitivity analysis for **developing country**

**Supplementary S12.7 –** Sensitivity analysis for **Intervention duration of ≥ 8 weeks**

**Supplementary S12.8 –** Sensitivity analysis for **Intervention duration of ＜ 8 weeks**

**Supplementary S12.9 –** Network evidence graph for Subclinical baseline severity

**Supplementary S12.10 –** Network evidence graph for Moderate to Severe baseline severity

**Supplementary S12.11 –** Network evidence graph for Severe baseline severity

**Supplementary S12.12 –** Forest plot and certainty evidence for Subclinical baseline severity

**Supplementary S12.13 –** Forest plot and certainty evidence for Moderate to Severe baseline severity

**Supplementary S12.14 –** Forest plot and certainty evidence for Severe baseline severity

**Supplementary S12.15 –** Network evidence graph for Individual face-to-face format

**Supplementary S12.16 –** Network evidence graph for Group face-to-face format

**Supplementary S12.17 –** Network evidence graph for Online format

**Supplementary S12.18 –** Network evidence graph for Mixed format

**Supplementary S12.19 –** Forest plot and certainty evidence for Individual face-to-face format

**Supplementary S12.20 –** Forest plot and certainty evidence for Group face-to-face format

**Supplementary S12.21 –** Forest plot and certainty evidence for Online format

**Supplementary S12.22 –** Forest plot and certainty evidence for Mixed format

**Supplementary S12.23 –** Comparison-adjusted funnel plot for **developed country**

**Supplementary S12.24 –** Comparison-adjusted funnel plot for **developing country**

**Supplementary S12.25** – Comparison-adjusted funnel plot for Intervention duration of ≥ 8 weeks

**Supplementary S12.26 –** Comparison-adjusted funnel plot for **Intervention duration of ＜ 8 weeks**

**Supplementary S12.27 –** Comparison-adjusted funnel plot for Subclinical baseline severity

**Supplementary S12.28 –** Comparison-adjusted funnel plot for Moderate to Severe baseline severity

**Supplementary S12.29 –** Comparison-adjusted funnel plot for Severe baseline severity

**Supplementary S12.30 –** Comparison-adjusted funnel plot for Individual face-to-face format

**Supplementary S12.31 –** Comparison-adjusted funnel plot for Group face-to-face format

**Supplementary S12.32 –** Comparison-adjusted funnel plot for Online format

**Supplementary S12.33 –** Comparison-adjusted funnel plot for Mixed format

**Supplementary S12.34 –** League table for **developed country**

**Supplementary S12.35 –** League table for **developing country**

**Supplementary S12.36 –** League table for **Intervention duration of ≥ 8 weeks**

**Supplementary S12.37 –** League table for **Intervention duration of ＜ 8 weeks**

**Supplementary S12.38 –** League table for **Subclinical baseline severity**

**Supplementary S12.39 –** League table for **Moderate to Severe baseline severity**

**Supplementary S12.40 –** League table for S**evere baseline severity**

**Supplementary S12.41 –** League table for **Individual face-to-face format**

**Supplementary S12.42 –** League table for **Group face-to-face format**

**Supplementary S12.43 –** League table for Online **format**

**Supplementary S12.44 –** League table for Mixed **format**

**Supplementary S12.45** – Surface Under the Cumulative Ranking curve Score and Ranking for **developed country**

**Supplementary S12.46** – Surface Under the Cumulative Ranking curve Score and Ranking for **developing country**

**Supplementary S12.47** – Surface Under the Cumulative Ranking curve Score and Ranking for **Intervention duration of ≥ 8 weeks**

**Supplementary S12.48** – Surface Under the Cumulative Ranking curve Score and Ranking for **Intervention duration of ＜ 8 weeks**

**Supplementary S12.49** – Surface Under the Cumulative Ranking curve Score and Ranking for Subclinical baseline severity

**Supplementary S12.50** – Surface Under the Cumulative Ranking curve Score and Ranking for Moderate to Severe baseline severity

**Supplementary S12.51** – Surface Under the Cumulative Ranking curve Score and Ranking for Severe baseline severity

**Supplementary S12.52** – Surface Under the Cumulative Ranking curve Score and Ranking for Individual face-to-face format

**Supplementary S12.53** – Surface Under the Cumulative Ranking curve Score and Ranking for Group face-to-face format

**Supplementary S12.54** – Surface Under the Cumulative Ranking curve Score and Ranking for Online format

**Supplementary S12.55** – Surface Under the Cumulative Ranking curve Score and Ranking for Mixed format

**Supplementary S12.56** – Surface Under the Cumulative Ranking curve Probability Sorting Plot for d**eveloped country**

**Supplementary S12.57** – Surface Under the Cumulative Ranking curve Probability Sorting Plot for developing country

**Supplementary S12.58** – Surface Under the Cumulative Ranking curve Probability Sorting Plot for developing country

**Supplementary S12.59** – Surface Under the Cumulative Ranking curve Probability Sorting Plot for **Intervention duration of ＜ 8 weeks**

**Supplementary S12.60** – Surface Under the Cumulative Ranking curve Probability Sorting Plot for **Subclinical baseline severity**

**Supplementary S12.61** – Surface Under the Cumulative Ranking curve Probability Sorting Plot for **Moderate to Severe baseline severity**

**Supplementary S12.62** – Surface Under the Cumulative Ranking curve Probability Sorting Plot for **Severe baseline severity**

**Supplementary S12.63**– Surface Under the Cumulative Ranking curve Probability Sorting Plot for **Individual face-to-face format**

**Supplementary S12.64**– Surface Under the Cumulative Ranking curve Probability Sorting Plot for **Group face-to-face**

**format**

**Supplementary S12.65**– Surface Under the Cumulative Ranking curve Probability Sorting Plot for **Online format**

**Supplementary S12.66**– Surface Under the Cumulative Ranking curve Probability Sorting Plot for **Mixed format**

**Supplementary S13 –** GRADE assessment

**Supplementary S13.1** – The Grading of Recommendations Assessment, Development and Evaluation (GRADE) assessment for adult social anxiety

**Supplementary S13.2** – The Grading of Recommendations Assessment, Development and Evaluation (GRADE) assessment for developed country

**Supplementary S13.3** – The Grading of Recommendations Assessment, Development and Evaluation (GRADE) assessment for developing country

**Supplementary S13.4** – The Grading of Recommendations Assessment, Development and Evaluation (GRADE) assessment for Intervention duration of ≥ 8 weeks

**Supplementary S13.5** – The Grading of Recommendations Assessment, Development and Evaluation (GRADE) assessment for Intervention duration of ＜ 8 weeks

**Supplementary S13.6** – The Grading of Recommendations Assessment, Development and Evaluation (GRADE) assessment for Subclinical baseline severity

**Supplementary S13.7** – The Grading of Recommendations Assessment, Development and Evaluation (GRADE) assessment for Moderate to Severe baseline severity

**Supplementary S13.8** – The Grading of Recommendations Assessment, Development and Evaluation (GRADE) assessment for Severe baseline severity

**Supplementary S13.9** – The Grading of Recommendations Assessment, Development and Evaluation (GRADE) assessment for Individual face-to-face format

**Supplementary S13.10** – The Grading of Recommendations Assessment, Development and Evaluation (GRADE) assessment for Group face-to-face format

**Supplementary S13.11** – The Grading of Recommendations Assessment, Development and Evaluation (GRADE) assessment for Online format

**Supplementary S13.12** – The Grading of Recommendations Assessment, Development and Evaluation (GRADE) assessment for Mixed format

**Supplementary S1 –** Database search strategy

**Supplementary S1.1** Database search strategy - Pubmed

| **Index** | **Search strategy** | **Results** |
| --- | --- | --- |
| #1 | ((((((((((((((((((((((((((("Exercise"[Mesh]) OR "Sports"[Mesh]) OR "Therapeutics"[Mesh]) OR "Mindfulness"[Mesh]) OR "Relaxation"[Mesh]) OR "Relaxation Therapy"[Mesh]) OR "Yoga"[Mesh]) OR "Music"[Mesh]) OR "Music Therapy"[Mesh]) OR "Education"[Mesh]) OR "Qigong"[Mesh]) OR "Art"[Mesh]) OR "Art Therapy"[Mesh]) OR "Dancing"[Mesh]) OR "Massage"[Mesh]) OR "Walking"[Mesh]) OR "Acupuncture"[Mesh]) OR "Acupuncture Therapy"[Mesh]) OR "Internet"[Mesh]) OR "Exercise Movement Techniques"[Mesh]) OR "Swimming"[Mesh]) OR "Hydrotherapy"[Mesh]) OR "Acupressure"[Mesh]) OR "Musculoskeletal Manipulations"[Mesh]) OR "Movement"[Mesh]) OR "Animal Assisted Therapy"[Mesh]) OR "Disease Management"[Mesh]) OR "Telemedicine"[Mesh] | 7,231,035 |
| #2 | "exercise*"[Title/Abstract] OR "sports"[Title/Abstract] OR "Therapeutics"[Title/Abstract] OR "therap*"[Title/Abstract] OR "mindfulness"[Title/Abstract] OR "relaxation"[Title/Abstract] OR "yoga"[Title/Abstract] OR ("music music"[All Fields] AND "threapy"[Title/Abstract]) OR "education"[Title/Abstract] OR "Qigong"[Title/Abstract] OR ("art art"[All Fields] AND "threapy"[Title/Abstract]) OR "Dancing"[Title/Abstract] OR "physical activit*"[Title/Abstract] OR "training*"[Title/Abstract] OR "ch i kung"[Title/Abstract] OR "walk"[Title/Abstract] OR "CBT"[Title/Abstract] OR "Game"[Title/Abstract] OR "massage"[Title/Abstract] OR "acupuncture"[Title/Abstract] OR "behavioral activation"[Title/Abstract] OR "acceptance"[Title/Abstract] OR "ACT"[Title/Abstract] OR "MCT"[Title/Abstract] OR "CFT"[Title/Abstract] OR "internet"[Title/Abstract] OR "VR"[Title/Abstract] OR "HIIT"[Title/Abstract] OR "pilates"[Title/Abstract] OR "core stabiliz*"[Title/Abstract] OR "swim"[Title/Abstract] OR "hydrotherapy"[Title/Abstract] OR "tuina"[Title/Abstract] OR "acupressure"[Title/Abstract] OR "reflexology"[Title/Abstract] OR "essential oil*"[Title/Abstract] OR "movement"[Title/Abstract] OR "animal-assisted"[Title/Abstract] OR "management"[Title/Abstract] OR "support*"[Title/Abstract] OR "self-help"[Title/Abstract] OR "mobile health"[Title/Abstract] OR "mHealth"[Title/Abstract] OR "app-based"[Title/Abstract] | 9,494,651 |
| #3 | #1 OR #2 | 13,749,062 |
| #4 | "Phobia, Social"[Mesh] | 1,595 |
| #5 | Phobia, Social[Title/Abstract] OR Social Phobia*[Title/Abstract] OR Social Anxiet*[Title/Abstract] OR Anxiet* Disorder, Social[Title/Abstract] OR Social Evaluation Phobia[Title/Abstract] OR Sociophobia*[Title/Abstract] OR Fear of Social Evaluation[Title/Abstract] OR Evaluation Fear, Social[Title/Abstract] OR Social Evaluation Fear[Title/Abstract] OR Phobia*, Social Evaluation[Title/Abstract] OR Social Evaluation Phobia*[Title/Abstract] OR Anxiet*, Social[Title/Abstract] OR SAD[Title/Abstract] OR liebowitz social anxiety scale[Title/Abstract] OR LSAS[Title/Abstract] OR social interaction anxiety[Title/Abstract] OR social avoidance and distress[Title/Abstract] OR fear of negative evaluation[Title/Abstract] OR FNE[Title/Abstract] OR fear of positive evaluation[Title/Abstract] OR FPE[Title/Abstract] | 27,107 |
| #6 | #4 OR #5 | 27,207 |
| #7 | "Randomized Controlled Trial" [Publication Type] | 650,827 |
| #8 | randomized controlled trial*[Title/Abstract] OR randomized clinical trial*[Title/Abstract] OR randomized controlled clinical trial*[Title/Abstract] OR RCT[Title/Abstract] OR random control test*[Title/Abstract] OR randomized controlled test*[Title/Abstract] OR randomized comparison stud*[Title/Abstract] OR randomized experiment*[Title/Abstract] OR placebo*[Title/Abstract] OR random allocation[Title/Abstract] OR randomized[Title/Abstract] | 939,414 |
| #9 | #7 OR #8 | 1,171,301 |
| #10 | #3 AND #6 AND #9 | 2,103 |

**Search date: November 3, 2025.**

**Supplementary S1.2** Database search strategy - Web of Science

| **Index** | | **Search strategy** | **Results** | |
| --- | --- | --- | --- | --- |
| #1 | exercise* OR sports OR Therapeutics OR therap* OR mindfulness OR relaxation OR yoga OR music OR education OR Qigong OR art OR Dancing OR Physical Activit* OR training* OR Chi Kung OR walk OR CBT OR Game OR massage OR acupuncture OR behavioral activation OR acceptance OR ACT OR MCT OR CFT OR internet OR VR OR HIIT OR pilates OR core stabiliz* OR swim OR hydrotherapy OR tuina OR acupressure OR reflexology OR essential oil* OR movement OR animal-assisted OR management OR support* OR self-help OR mobile health OR mHealth OR app-based (Abstract) | | | 14,544,191 |
| #2 | Phobia, Social OR Social Phobia* OR Social Anxiet* OR Anxiet* Disorder, Social OR Social Evaluation Phobia OR Sociophobia* OR Fear of Social Evaluation OR Evaluation Fear, Social OR Social Evaluation Fear OR Phobia*, Social Evaluation OR Social Evaluation Phobia* OR Anxiet*, Social OR SAD OR liebowitz social anxiety scale OR LSAS OR social interaction anxiety OR social avoidance and distress OR fear of negative evaluation OR FNE OR fear of positive evaluation OR FPE (Abstract) | | | 92,011 |
| #3 | randomized controlled trial* OR randomized clinical trial* OR randomized controlled clinical trial* OR RCT OR random control test* OR randomized controlled test* OR randomized comparison stud* OR randomized experiment* OR placebo* OR random allocation OR randomized(Abstract) | | | 779,746 |
| #4 | #3 AND #2 AND #1 | | | 4,121 |

**Search date: November 3, 2025.**

**Supplementary S1.3** Database Search strategy - EBSCO (All Database )

| **Index** | **Search strategy** | **Results** | |
| --- | --- | --- | --- |
| #1 | AB (exercise* OR sports OR Therapeutics OR therap* OR mindfulness OR relaxation OR yoga OR music OR education OR Qigong OR art OR Dancing OR Physical Activit* OR training* OR Chi Kung OR walk OR CBT OR Game OR massage OR acupuncture OR behavioral activation OR acceptance OR ACT OR MCT OR CFT OR internet OR VR OR HIIT OR pilates OR core stabiliz* OR swim OR hydrotherapy OR tuina OR acupressure OR reflexology OR essential oil* OR movement OR animal-assisted OR management OR support* OR self-help OR mobile health OR mHealth OR app-based) | | 24,036,362 |
| #2 | AB (Phobia, Social OR Social Phobia* OR Social Anxiet* OR Anxiet* Disorder, Social OR Social Evaluation Phobia OR Sociophobia* OR Fear of Social Evaluation OR Evaluation Fear, Social OR Social Evaluation Fear OR Phobia*, Social Evaluation OR Social Evaluation Phobia* OR Anxiet*, Social OR SAD OR liebowitz social anxiety scale OR LSAS OR social interaction anxiety OR social avoidance and distress OR fear of negative evaluation OR FNE OR fear of positive evaluation OR FPE) | | 92,557 |
| #3 | AB (randomized controlled trial* OR randomized clinical trial* OR randomized controlled clinical trial* OR RCT OR random control test* OR randomized controlled test* OR randomized comparison stud* OR randomized experiment* OR placebo* OR random allocation OR randomized) | | 1,575,928 |
| #4 | #3 AND #2 AND #1 | | 3,411 |

**Search date: November 3, 2025.**

**Supplementary S1.4** Database search strategy - Cochrane

| **Index** | | **search strategy** | **Results** | |
| --- | --- | --- | --- | --- |
| #1 | MeSH descriptor: [Exercise] explode all trees | | | 41,063 |
| #2 | MeSH descriptor: [Sports] explode all trees | | | 23,134 |
| #3 | MeSH descriptor: [Therapeutics] explode all trees | | | 437,465 |
| #4 | MeSH descriptor: [Mindfulness] explode all trees | | | 2,635 |
| #5 | MeSH descriptor: [Relaxation] explode all trees | | | 2,209 |
| #6 | MeSH descriptor: [Yoga] explode all trees | | | 1,209 |
| #7 | MeSH descriptor: [Music] explode all trees | | | 1,281 |
| #8 | MeSH descriptor: [Education] explode all trees | | | 47,878 |
| #9 | MeSH descriptor: [Qigong] explode all trees | | | 188 |
| #10 | MeSH descriptor: [Art] explode all trees | | | 448 |
| #11 | MeSH descriptor: [Dancing] explode all trees | | | 319 |
| #12 | MeSH descriptor: [Walking] explode all trees | | | 8,593 |
| #13 | MeSH descriptor: [Massage] explode all trees | | | 1,777 |
| #14 | MeSH descriptor: [Acupuncture] explode all trees | | | 216 |
| #15 | MeSH descriptor: [Internet] explode all trees | | | 7,016 |
| #16 | MeSH descriptor: [Swimming] explode all trees | | | 670 |
| #17 | MeSH descriptor: [Hydrotherapy] explode all trees | | | 1,944 |
| #18 | MeSH descriptor: [Acupressure] explode all trees | | | 631 |
| #19 | MeSH descriptor: [Musculoskeletal Manipulations] explode all trees | | | 4,531 |
| #20 | MeSH descriptor: [Movement] explode all trees | | | 51,231 |
| #21 | MeSH descriptor: [Telemedicine] explode all trees | | | 5,810 |
| #22 | (exercise* OR sports OR Therapeutics OR therap* OR mindfulness OR relaxation OR yoga OR music OR education OR Qigong OR art OR Dancing OR Physical Activit* OR training* OR Chi Kung OR walk OR CBT OR Game OR massage OR acupuncture OR behavioral activation OR acceptance OR ACT OR MCT OR CFT OR internet OR VR OR HIIT OR pilates OR core stabiliz* OR swim OR hydrotherapy OR tuina OR acupressure OR reflexology OR essential oil* OR movement OR animal-assisted OR management OR support* OR self-help OR mobile health OR mHealth OR app-based):ti,kw | | | 1,463,736 |
| #23 | #1 OR #2 OR #3 # OR #4 OR #5 OR #6 OR #7 OR #8 OR #9 OR #10 OR #11 OR #12 OR #13 OR #14 OR #15 OR #16 OR #17 OR #18 OR #19 OR #20 OR #21 OR #22 | | | 1,543,925 |
| #24 | MeSH descriptor: [Phobia, Social] explode all trees | | | 479 |
| #25 | (Phobia, Social OR Social Phobia* OR Social Anxiet* OR Anxiet* Disorder, Social OR Social Evaluation Phobia OR Sociophobia* OR Fear of Social Evaluation OR Evaluation Fear, Social OR Social Evaluation Fear OR Phobia*, Social Evaluation OR Social Evaluation Phobia* OR Anxiet*, Social OR SAD OR liebowitz social anxiety scale OR LSAS OR social interaction anxiety OR social avoidance and distress OR fear of negative evaluation OR FNE OR fear of positive evaluation OR FPE):ti,ab | | | 20,725 |
| #26 | #24 OR #25 | | | 20,725 |
| #27 | #23AND #26 | | | 17,694 |

**Search date: November 3, 2025.**

**Supplementary S1.5** Database search strategy - Embase

| **Index** | **search strategy** | **Results** |
| --- | --- | --- |
| **#1** | 'exercise'/exp OR 'sport'/exp OR 'therapy'/exp OR 'mindfulness'/exp OR 'leisure'/exp OR 'yoga'/exp OR 'music'/exp OR 'education'/exp OR 'qigong'/exp OR 'art'/exp OR 'dancing'/exp OR 'walking'/exp OR 'cognitive behavioral therapy'/exp OR 'massage'/exp OR 'game'/exp OR 'acupuncture'/exp OR 'behavioral activation'/exp OR 'internet'/exp OR 'pilates'/exp OR 'swimming'/exp OR 'hydrotherapy'/exp OR 'acupressure'/exp OR 'reflexology'/exp OR 'movement (physiology)'/exp OR 'animal assisted therapy'/exp OR 'management'/exp OR 'mobile health application'/exp | 21,839,765 |
| #2 | 'exercise*':ab,ti OR 'sports':ab,ti OR 'therapeutics':ab,ti OR 'therap*':ab,ti OR 'mindfulness':ab,ti OR 'relaxation':ab,ti OR 'yoga':ab,ti OR 'music':ab,ti OR 'education':ab,ti OR 'qigong':ab,ti OR 'art':ab,ti OR 'dancing':ab,ti OR 'physical activit*':ab,ti OR 'training*':ab,ti OR 'chi kung':ab,ti OR 'walk':ab,ti OR 'cbt':ab,ti OR 'game':ab,ti OR 'massage':ab,ti OR 'acupuncture':ab,ti OR 'behavioral activation':ab,ti OR 'acceptance':ab,ti OR 'act':ab,ti OR 'mct':ab,ti OR 'cft':ab,ti OR 'internet':ab,ti OR 'vr':ab,ti OR 'hiit':ab,ti OR 'pilates':ab,ti OR 'core stabiliz*':ab,ti OR 'swim':ab,ti OR 'hydrotherapy':ab,ti OR 'tuina':ab,ti OR 'acupressure':ab,ti OR 'reflexology':ab,ti OR 'essential oil*':ab,ti OR 'movement':ab,ti OR 'animal-assisted':ab,ti OR 'management':ab,ti OR 'support*':ab,ti OR 'self-help':ab,ti OR 'mobile health':ab,ti OR 'mhealth':ab,ti OR 'app-based':ab,ti | 12,859,861 |
| #3 | #1 OR #2 | 24,650,941 |
| #4 | 'social phobia'/exp | 16,847 |
| **#5** | 'Phobia, Social':ab,ti OR 'Social Phobia*':ab,ti OR 'Social Anxiet*':ab,ti OR 'Anxiet* Disorder, Social':ab,ti OR 'Social Evaluation Phobia':ab,ti OR 'Sociophobia*':ab,ti OR 'Fear of Social Evaluation':ab,ti OR 'Evaluation Fear, Social':ab,ti OR 'Social Evaluation Fear':ab,ti OR 'Phobia*, Social Evaluation':ab,ti OR 'Social Evaluation Phobia*':ab,ti OR 'Anxiet*, Social':ab,ti OR 'SAD':ab,ti OR 'liebowitz social anxiety scale':ab,ti OR 'LSAS':ab,ti OR 'social interaction anxiety':ab,ti OR 'social avoidance and distress':ab,ti OR 'fear of negative evaluation':ab,ti OR 'FNE':ab,ti OR 'fear of positive evaluation':ab,ti OR 'FPE':ab,ti | 38,053 |
| #6 | #4 OR #5 | 45,058 |
| #7 | 'randomized controlled trial'/exp | 1,122,990 |
| #8 | 'randomized controlled trial*':ab,ti OR 'randomized clinical trial*':ab,ti OR 'randomized controlled clinical trial*':ab,ti OR 'rct':ab,ti OR 'random control test*':ab,ti OR 'randomized controlled test*':ab,ti OR 'randomized comparison stud*':ab,ti OR 'randomized experiment*':ab,ti OR 'placebo*':ab,ti OR 'random allocation':ab,ti OR 'randomized':ab,ti | 1,556,451 |
| #9 | #7 OR #8 | 1,875,935 |
| #10 | #3 AND #6 AND #9 | 5,438 |

**Search date: November 3, 2025.**

**Supplementary S2 –** Conversion formula for standard deviation (SD).

**Standard Error to Standard Deviation:**

SD=SE×$\sqrt{N}$ where SE is the standard error and N is the sample size.

**95% Confidence Interval to Standard Deviation:**

**1** If the sample size of the test and control groups is greater than or equal to 100: SD=$\sqrt{N}$×(Upper limit of credible intervals - Lower limit of credible intervals)/3.92

**2** If the sample size of the test and control groups is less than or equal to 60: SD=$\sqrt{N}$×(Upper limit of credible intervals - Lower limit of credible intervals)/ tinv (1-0.95,n-1)

**3** For studies with sample sizes between 60 and 100 in each group, both of the above methods can be used.

**Range** **converted SD:**

SD= (Upper limit - lower limit)/4

**Quartile** **converted SD:**

SD= (Upper limit - lower limit)/1.35

Tinv: Represent probabilities, degrees of freedom in excel sheet

**Supplementary S3 –** Definitions and Classification of Interventions and Controls

| **Group** | **Abbreviation** | **Definition / Mechanism** | **Included Interventions** |
| --- | --- | --- | --- |
| **Control Group** | **CON** | No direct therapeutic effect; serves solely as a benchmark to control for placebo effects. | Includes waiting list (WL), placebo/sham control, treatment as usual (TAU), and routine care. |
| **Virtual Reality Exposure Therapy** | **VRET** | Desensitisation through repeated exposure to virtual or real anxiety-provoking scenarios to diminish fear responses. | VRET and exposure-based programmes delivered primarily via VR technology. |
| **Cognitive Behavioral Therapy** | **CBT** | Primary approach centres on "cognitive restructuring + behavioural training" to modify maladaptive cognitions. | CBT programmes incorporating cognitive restructuring and behavioural/exposure elements. |
| **Acceptance and Commitment Therapy** | **ACT** | Enhances psychological flexibility to embrace anxiety; distinct from symptom-elimination logic. | ACT delivered in-person or online, including VR-assisted ACT as the core component. |
| **Relaxation Therapy** | **RT** | Focuses on cultivating non-judgmental, present-moment awareness. It operates through attentional regulation, cognitive decentering (observing thoughts and emotions without engaging or reacting), and emotional acceptance, differing fundamentally from pure somatic de-arousal. | Mindfulness-Based Stress Reduction (MBSR). |
|  |  | Focuses on physiological de-arousal and reducing somatic symptoms of anxiety. It works primarily via parasympathetic nervous system activation to alleviate physical tension, without directly targeting or restructuring cognitive or attentional processes. | Applied Relaxation (AR), and relaxation protocols. |
| **Social and Interpersonal Skills** | **SIS** | Addresses social deficits through training to enhance interaction abilities. | Social efficacy therapy, cognitive social skills training, and skills-oriented programmes. |
| **Combination therapy** | **CT** | Integrates two or more core mechanisms for synergistic enhancement without a single dominant component. | Explicit combinations (e.g., CBT + Attention Bias Modification; Emotional Writing + Skills Training). |
| **Psychotherapy** | **PT** | Focuses on psychodynamic or interpersonal regulation to improve underlying mechanisms. | Includes psychodynamic therapy, interpersonal psychotherapy (IPT), and bona fide psychotherapies. |
| **Attention training** | **AT** | Cognitive-targeted intervention adjusting attentional allocation to reduce focus on social threats. | Includes Attentional Bias Modification (ABM) and Interpretation Bias Modification. |
| **Reading therapy** | **READ** | Delivers coping strategies through therapeutic reading (Bibliotherapy). | Guided self-help books/manuals and text-based reading programmes. |

**Notes: CON:**Control group **VRET:**Virtual Reality Exposure Therapy **CBT:**Cognitive behavioral therapy **ACT:**Acceptance and Commitment Therapy **RT:**Relaxation therapy **SIS:**Social and Interpersonal Skills **CT**:Combination therapy **PT:**Psychotherapy **AT:**Attention training **READ:**Reading therapy

**Supplementary S4 –** Inclusion in the list of studies.

Bouchard, S., Dumoulin, S., Robillard, G., Guitard, T., Klinger, É., Forget, H., Loranger, C., & Roucaut, F. X. (2017). Virtual reality compared with in vivo exposure in the treatment of social anxiety disorder: a three-arm randomised controlled trial. *The British journal of psychiatry : the journal of mental science, 210*(4), 276–283. [https://doi.org/10.1192/bjp.bp.116.184234](Bouchard, S., Dumoulin, S., Robillard, G., Guitard, T., Klinger, É., Forget, H., Loranger, C., & Roucaut, F. X. (2017). Virtual reality compared with in vivo exposure in the treatment of social anxiety disorder: a three-arm randomised controlled trial. The)

Gorinelli, S., Gallego, A., Lappalainen, P., & Lappalainen, R. (2023). Virtual reality acceptance and commitment therapy intervention for social and public speaking anxiety: A randomized controlled trial. *Journal of Contextual Behavioral Science, 28*, 289–299. <https://doi.org/10.1016/j.jcbs.2023.05.004>

Olthuis, J. V., Watt, M. C., Mackinnon, S. P., & Stewart, S. H. (2014). Telephone-delivered cognitive behavioral therapy for high anxiety sensitivity: a randomized controlled trial. *Journal of consulting and clinical psychology, 82*(6), 1005–1022. <https://doi.org/10.1037/a0037027>

Recabarren, R. E., Gaillard, C., Guillod, M., & Martin-Soelch, C. (2019). Short-Term Effects of a Multidimensional Stress Prevention Program on Quality of Life, Well-Being and Psychological Resources. A Randomized Controlled Trial. *Frontiers in psychiatry, 10*, 88. <https://doi.org/10.3389/fpsyt.2019.00088>

Piet, J., Hougaard, E., Hecksher, M. S., & Rosenberg, N. K. (2010). A randomized pilot study of mindfulness-based cognitive therapy and group cognitive-behavioral therapy for young adults with social phobia. *Scandinavian journal of psychology, 51*(5), 403–410. <https://doi.org/10.1111/j.1467-9450.2009.00801.x>

Knijnik, D. Z., Kapczinski, F., Chachamovich, E., Margis, R., & Eizirik, C. L. (2004). Psicoterapia psicodinâmica em grupo para fobia social generalizada [Psychodynamic group treatment for generalized social phobia]. *Revista brasileira de psiquiatria (Sao Paulo, Brazil : 1999), 26*(2), 77–81. <https://doi.org/10.1590/s1516-44462004000200003>

Amir, N., & Taylor, C. T. (2012). Interpretation training in individuals with generalized social anxiety disorder: a randomized controlled trial. *Journal of consulting and clinical psychology, 80*(3), 497–511. <https://doi.org/10.1037/a0026928>

Nordmo, M., Sinding, A. I., Carlbring, P., Andersson, G., Havik, O. E., & Nordgreen, T. (2015). Internet-delivered cognitive behavioural therapy with and without an initial face-to-face psychoeducation session for social anxiety disorder: A pilot randomized controlled trial. *Internet Interventions, 2*(4), 429–436. <https://doi.org/10.1016/j.invent.2015.10.003>

Berger, T., Hohl, E., & Caspar, F. (2009). Internet-based treatment for social phobia: a randomized controlled trial. *Journal of clinical psychology, 65*(10), 1021–1035. <https://doi.org/10.1002/jclp.20603>

Andersson, G., Carlbring, P., Holmström, A., Sparthan, E., Furmark, T., Nilsson-Ihrfelt, E., Buhrman, M., & Ekselius, L. (2006). Internet-based self-help with therapist feedback and in vivo group exposure for social phobia: a randomized controlled trial. *Journal of consulting and clinical psychology, 74*(4), 677–686. <https://doi.org/10.1037/0022-006X.74.4.677>

Rapee, R. M., MacLeod, C., Carpenter, L., Gaston, J. E., Frei, J., Peters, L., & Baillie, A. J. (2013). Integrating cognitive bias modification into a standard cognitive behavioural treatment package for social phobia: a randomized controlled trial. *Behavioral research and therapy, 51*(4-5), 207–215. <https://doi.org/10.1016/j.brat.2013.01.005>

Blanco, C., Heimberg, R. G., Schneier, F. R., Fresco, D. M., Chen, H., Turk, C. L., Vermes, D., Erwin, B. A., Schmidt, A. B., Juster, H. R., Campeas, R., & Liebowitz, M. R. (2010). A placebo-controlled trial of phenelzine, cognitive behavioral group therapy, and their combination for social anxiety disorder. *Archives of general psychiatry, 67*(3), 286–295. <https://doi.org/10.1001/archgenpsychiatry.2010.11>

Handley, A. K., Egan, S. J., Kane, R. T., & Rees, C. S. (2015). A randomised controlled trial of group cognitive behavioural therapy for perfectionism. *Behavioral research and therapy, 68*, 37–47. <https://doi.org/10.1016/j.brat.2015.02.006>

de Rutte, J., Myruski, S., Davis, E., Findley, A., & Dennis-Tiwary, T. A. (2025). A randomized clinical trial investigating the clinical impact of a game-based digital therapeutic for social anxiety disorder. *Journal of anxiety disorders, 111*, 103000. <https://doi.org/10.1016/j.janxdis.2025.103000>

Gharraee, B., Zahedi Tajrishi, K., Ramezani Farani, A., Bolhari, J., & Farahani, H. (2018). A randomized controlled trial of compassion focused therapy for social anxiety disorder. *Iranian Journal of Psychiatry and Behavioral Sciences, 12*(4). <https://doi.org/10.5812/ijpbs.80945>

Ezenwaji, C. O., Aloh, H. E., Okeke, P. M. D., Osilike, C. C., Ekwealor, N. E., Koledoye, U. L., Ilechukwu, L. C., & Onwuadi, C. C. (2021). Managing social anxiety among undergraduate adult education and extra-mural studies students: An intervention study. *Medicine, 100*(42), e27596. <https://doi.org/10.1097/MD.0000000000027596>

Bunnell, B. E., Beidel, D. C., & Mesa, F. (2013). A randomized trial of attention training for generalized social phobia: does attention training change social behavior?. *Behavior therapy, 44*(4), 662–673. <https://doi.org/10.1016/j.beth.2013.04.010>

Krafft, J., Twohig, M. P., & Levin, M. E. (2020). A randomized trial of acceptance and commitment therapy and traditional cognitive-behavioral therapy self-help books for social anxiety. *Cognitive Therapy and Research, 44*, 954–966. <https://doi.org/10.1007/s10608-020-10114-3>

Amir, N., Beard, C., Taylor, C. T., Klumpp, H., Elias, J., Burns, M., & Chen, X. (2009). Attention training in individuals with generalized social phobia: A randomized controlled trial. *Journal of consulting and clinical psychology, 77*(5), 961–973. <https://doi.org/10.1037/a0016685>

Vally, Z., Shah, H., Varga, S. I., Hassan, W., Kashakesh, M., Albreiki, W., & Helmy, M. (2024). An internet-delivered acceptance and commitment therapy program for anxious affect, depression, and wellbeing: A randomized, parallel, two-group, waitlist-controlled trial in a Middle Eastern sample of college students. *PloS one, 19*(12), e0313243. <https://doi.org/10.1371/journal.pone.0313243>

Lazarov, A., Marom, S., Yahalom, N., Pine, D. S., Hermesh, H., & Bar-Haim, Y. (2018). Attention bias modification augments cognitive-behavioral group therapy for social anxiety disorder: a randomized controlled trial. *Psychological medicine, 48*(13), 2177–2185. <https://doi.org/10.1017/S003329171700366X>

Bjornsson, A. S., Bidwell, L. C., Brosse, A. L., Carey, G., Hauser, M., Mackiewicz Seghete, K. L., Schulz-Heik, R. J., Weatherley, D., Erwin, B. A., & Craighead, W. E. (2011). Cognitive-behavioral group therapy versus group psychotherapy for social anxiety disorder among college students: a randomized controlled trial. *Depression and anxiety, 28*(11), 1034–1042. <https://doi.org/10.1002/da.20877>

Mueller, N. E., & Cougle, J. R. (2023). Building Closer Friendships in social anxiety disorder: A randomized control trial of an internet-based intervention. *Journal of behavior therapy and experimental psychiatry, 78*, 101799. <https://doi.org/10.1016/j.jbtep.2022.101799>

Clark, D. M., Ehlers, A., McManus, F., Hackmann, A., Fennell, M., Campbell, H., Flower, T., Davenport, C., & Louis, B. (2003). Cognitive therapy versus fluoxetine in generalized social phobia: a randomized placebo-controlled trial. *Journal of consulting and clinical psychology, 71*(6), 1058–1067. <https://doi.org/10.1037/0022-006X.71.6.1058>

D'El Rey G.J.F., Lacava J.P.L., Cejkinski A., Mello S.L. (2008). Cognitive-behavioral group treatment in social phobia: 12-Week outcome. *Revista de Psiquiatria Clinica, 35*(2), 79-83. <http://dx.doi.org/10.1590/S0101-60832008000200006>

Dagöö, J., Asplund, R. P., Bsenko, H. A., Hjerling, S., Holmberg, A., Westh, S., Öberg, L., Ljótsson, B., Carlbring, P., Furmark, T., & Andersson, G. (2014). Cognitive behavior therapy versus interpersonal psychotherapy for social anxiety disorder delivered via smartphone and computer: a randomized controlled trial. *Journal of anxiety disorders, 28*(4), 410–417. <https://doi.org/10.1016/j.janxdis.2014.02.003>

Cottraux, J., Note, I., Albuisson, E., Yao, S. N., Note, B., Mollard, E., Bonasse, F., Jalenques, I., Guérin, J., & Coudert, A. J. (2000). Cognitive behavior therapy versus supportive therapy in social phobia: a randomized controlled trial. *Psychotherapy and psychosomatics, 69*(3), 137–146. <https://doi.org/10.1159/000012382>

Heimberg, R. G., Liebowitz, M. R., Hope, D. A., Schneier, F. R., Holt, C. S., Welkowitz, L. A., Juster, H. R., Campeas, R., Bruch, M. A., Cloitre, M., Fallon, B., & Klein, D. F. (1998). Cognitive behavioral group therapy vs phenelzine therapy for social phobia: 12-week outcome. *Archives of general psychiatry, 55*(12), 1133–1141. <https://doi.org/10.1001/archpsyc.55.12.1133>

Yoshinaga, N., Matsuki, S., Niitsu, T., Sato, Y., Tanaka, M., Ibuki, H., Takanashi, R., Ohshiro, K., Ohshima, F., Asano, K., Kobori, O., Yoshimura, K., Hirano, Y., Sawaguchi, K., Koshizaka, M., Hanaoka, H., Nakagawa, A., Nakazato, M., Iyo, M., & Shimizu, E. (2016). Cognitive Behavioral Therapy for Patients with Social Anxiety Disorder Who Remain Symptomatic following Antidepressant Treatment: A Randomized, Assessor-Blinded, Controlled Trial. *Psychotherapy and psychosomatics, 85*(4), 208–217. <https://doi.org/10.1159/000444221>

Kählke, F., Buntrock, C., Smit, F., Berger, T., Baumeister, H., & Ebert, D. D. (2023). Long-Term Outcomes and Cost-Effectiveness of an Internet-Based Self-Help Intervention for Social Anxiety Disorder in University Students: Results of a Randomized Controlled Trial. *Depression and anxiety*, 2023, 7912017. <https://doi.org/10.1155/2023/7912017>

Clark, D. M., Ehlers, A., Hackmann, A., McManus, F., Fennell, M., Grey, N., Waddington, L., & Wild, J. (2006). Cognitive therapy versus exposure and applied relaxation in social phobia: A randomized controlled trial. *Journal of consulting and clinical psychology, 74*(3), 568–578. <https://doi.org/10.1037/0022-006X.74.3.568>

Boettcher, J., Hasselrot, J., Sund, E., Andersson, G., & Carlbring, P. (2014). Combining attention training with internet-based cognitive-behavioural self-help for social anxiety: a randomised controlled trial. *Cognitive behaviour therapy, 43*(1), 34–48. <https://doi.org/10.1080/16506073.2013.809141>

Borgeat, F., Stankovic, M., Khazaal, Y., Rouget, B. W., Baumann, M. C., Riquier, F., O'Connor, K., Jermann, F., Zullino, D., & Bondolfi, G. (2009). Does the form or the amount of exposure make a difference in the cognitive-behavioral therapy treatment of social phobia?. *The Journal of nervous and mental disease, 197*(7), 507–513. <https://doi.org/10.1097/NMD.0b013e3181aacc08>

Powell, J., Williams, V., Atherton, H., Bennett, K., Yang, Y., Davoudianfar, M., Hellsing, A., Martin, A., Mollison, J., Shanyinde, M., Yu, L. M., & Griffiths, K. M. (2020). Effectiveness and Cost-Effectiveness of a Self-Guided Internet Intervention for Social Anxiety Symptoms in a General Population Sample: Randomized Controlled Trial. *Journal of medical Internet research, 22*(1), e16804. <https://doi.org/10.2196/16804>

Abeditehrani, H., Dijk, C., Dehghani Neyshabouri, M., & Arntz, A. (2024). Effectiveness of cognitive behavioral group therapy, psychodrama, and their integration for treatment of social anxiety disorder: A randomized controlled trial. *Journal of behavior therapy and experimental psychiatry, 82*, 101908. <https://doi.org/10.1016/j.jbtep.2023.101908>

Bell, C. J., Colhoun, H. C., Carter, F. A., & Frampton, C. M. (2012). Effectiveness of computerised cognitive behaviour therapy for anxiety disorders in secondary care. *The Australian and New Zealand journal of psychiatry, 46*(7), 630–640. <https://doi.org/10.1177/0004867412437345>

Kählke, F., Berger, T., Schulz, A., Baumeister, H., Berking, M., Auerbach, R. P., Bruffaerts, R., Cuijpers, P., Kessler, R. C., & Ebert, D. D. (2019). Efficacy of an unguided internet-based self-help intervention for social anxiety disorder in university students: A randomized controlled trial. *International journal of methods in psychiatric research, 28*(2), e1766. <https://doi.org/10.1002/mpr.1766>

de Oliveira, I. R., Powell, V. B., Wenzel, A., Caldas, M., Seixas, C., Almeida, C., Bonfim, T., Grangeon, M. C., Castro, M., Galvão, A., de Oliveira Moraes, R., & Sudak, D. (2012). Efficacy of the trial-based thought record, a new cognitive therapy strategy designed to change core beliefs, in social phobia. *Journal of clinical pharmacy and therapeutics, 37*(3), 328–334. <https://doi.org/10.1111/j.1365-2710.2011.01299.x>

Garcia-Lopez, L. J., Olivares, J., Beidel, D., Albano, A. M., Turner, S., & Rosa, A. I. (2006). Efficacy of three treatment protocols for adolescents with social anxiety disorder: a 5-year follow-up assessment. *Journal of anxiety disorders, 20*(2), 175–191. <https://doi.org/10.1016/j.janxdis.2005.01.003>

Enock P.M., Hofmann S.G., McNally R.J. (2014). Attention bias modification training via smartphone to reduce social anxiety: A randomized, controlled multi-session experiment. *Cognitive Therapy and Research, 38*(2), 200-216. <http://dx.doi.org/10.1007/s10608-014-9606-z>

McCall, H. C., Richardson, C. G., Helgadottir, F. D., & Chen, F. S. (2018). Evaluating a Web-Based Social Anxiety Intervention Among University Students: Randomized Controlled Trial. *Journal of medical Internet research, 20*(3), e91. <https://doi.org/10.2196/jmir.8630>

Mall, A. K., Mehl, A., Kiko, S., Kleindienst, N., Salize, H. J., Hermann, C., Hoffmann, T., Bohus, M., & Steil, R. (2011). Evaluation of a DVD-based self-help program in highly socially anxious individuals--pilot study. *Behavior therapy, 42*(3), 439–448. <https://doi.org/10.1016/j.beth.2010.11.007>

Mattick, R. P., Peters, L., & Clarke, J. C. (1989). Exposure and cognitive restructuring for social phobia: A controlled study. *Behavior Therapy, 20*(1), 3–23. <https://doi.org/10.1016/S0005-7894(89)80115-7>

Goldin, P. R., Morrison, A., Jazaieri, H., Brozovich, F., Heimberg, R., & Gross, J. J. (2016). Group CBT versus MBSR for social anxiety disorder: A randomized controlled trial. *Journal of consulting and clinical psychology, 84*(5), 427–437. <https://doi.org/10.1037/ccp0000092>

Furmark, T., Carlbring, P., Hedman, E., Sonnenstein, A., Clevberger, P., Bohman, B., Eriksson, A., Hållén, A., Frykman, M., Holmström, A., Sparthan, E., Tillfors, M., Ihrfelt, E. N., Spak, M., Eriksson, A., Ekselius, L., & Andersson, G. (2009). Guided and unguided self-help for social anxiety disorder: randomised controlled trial. *The British journal of psychiatry : the journal of mental science, 195*(5), 440–447. <https://doi.org/10.1192/bjp.bp.108.060996>

Nilsson, J. E., Lundh, L. G., & Viborg, G. (2012). Imagery rescripting of early memories in social anxiety disorder: an experimental study. *Behavioral research and therapy, 50*(6), 387–392. <https://doi.org/10.1016/j.brat.2012.03.004>

Hyett, M. P., Bank, S. R., Lipp, O. V., Erceg-Hurn, D. M., Alvares, G. A., Maclaine, E., Puckridge, E., Hayes, S., & McEvoy, P. M. (2018). Attenuated Psychophysiological Reactivity following Single-Session Group Imagery Rescripting versus Verbal Restructuring in Social Anxiety Disorder: Results from a Randomized Controlled Trial. *Psychotherapy and psychosomatics, 87*(6), 340–349. <https://doi.org/10.1159/000493897>

McEvoy, P. M., Hyett, M. P., Bank, S. R., Erceg-Hurn, D. M., Johnson, A. R., Kyron, M. J., Saulsman, L. M., Moulds, M. L., Grisham, J. R., Holmes, E. A., Moscovitch, D. A., Lipp, O. V., Campbell, B. N. C., & Rapee, R. M. (2022). Imagery-enhanced v. verbally-based group cognitive behavior therapy for social anxiety disorder: a randomized clinical trial. *Psychological medicine, 52*(7), 1277–1286. <https://doi.org/10.1017/S0033291720003001>

Sigurðardóttir, S., Helgadóttir, F. D., Menzies, R. E., Sighvatsson, M. B., & Menzies, R. G. (2022). Improving adherence to a web-based cognitive-behavioural therapy program for social anxiety with group sessions: A randomised control trial. *Internet interventions, 28*, 100535. <https://doi.org/10.1016/j.invent.2022.100535>

Johansson, R., Hesslow, T., Ljótsson, B., Jansson, A., Jonsson, L., Färdig, S., Karlsson, J., Hesser, H., Frederick, R. J., Lilliengren, P., Carlbring, P., & Andersson, G. (2017). Internet-based affect-focused psychodynamic therapy for social anxiety disorder: A randomized controlled trial with 2-year follow-up. *Psychotherapy (Chicago, Ill.), 54*(4), 351–360. <https://doi.org/10.1037/pst0000147>

Carlbring, P., Apelstrand, M., Sehlin, H., Amir, N., Rousseau, A., Hofmann, S. G., & Andersson, G. (2012). Internet-delivered attention bias modification training in individuals with social anxiety disorder--a double blind randomized controlled trial. *BMC psychiatry, 12*, 66. <https://doi.org/10.1186/1471-244X-12-66>

Thew, G. R., Kwok, A. P. L., Chan, M. H. L., Powell, C. L. Y. M., Wild, J., Leung, P. W. L., & Clark, D. M. (2022). Internet-delivered cognitive therapy for social anxiety disorder in Hong Kong: A randomized controlled trial. *Internet Interventions, 28*. <https://doi.org/10.1016/j.invent.2022.100539>

Tulbure, B. T., Szentagotai, A., David, O., Ștefan, S., Månsson, K. N., David, D., & Andersson, G. (2015). Internet-delivered cognitive-behavioral therapy for social anxiety disorder in Romania: a randomized controlled trial. *PloS one, 10*(5), e0123997. <https://doi.org/10.1371/journal.pone.0123997>

Cougle, J. R., Wilver, N. L., Day, T. N., Summers, B. J., Okey, S. A., & Carlton, C. N. (2020). Interpretation Bias Modification Versus Progressive Muscle Relaxation for Social Anxiety Disorder: A Web-Based Controlled Trial. *Behavior therapy, 51*(1), 99–112. <https://doi.org/10.1016/j.beth.2019.05.009>

Lin, X. B., Lee, T. S., Cheung, Y. B., Ling, J., Poon, S. H., Lim, L., Zhang, H. H., Chin, Z. Y., Wang, C. C., Krishnan, R., & Guan, C. (2019). Exposure Therapy With Personalized Real-Time Arousal Detection and Feedback to Alleviate Social Anxiety Symptoms in an Analogue Adult Sample: Pilot Proof-of-Concept Randomized Controlled Trial. *JMIR mental health, 6*(6), e13869. <https://doi.org/10.2196/13869>

Salaberría, K., & Echeburúa, E. (1998). Long-term outcome of cognitive therapy's contribution to self-exposure in vivo to the treatment of generalized social phobia. *Behavior modification, 22*(3), 262–284. <https://doi.org/10.1177/01454455980223003>

Furmark, T., Carlbring, P., Hedman, E., Sonnenstein, A., Clevberger, P., Bohman, B., Eriksson, A., Hållén, A., Frykman, M., Holmström, A., Sparthan, E., Tillfors, M., Ihrfelt, E. N., Spak, M., Eriksson, A., Ekselius, L., & Andersson, G. (2009). Guided and unguided self-help for social anxiety disorder: randomised controlled trial. *The British journal of psychiatry : the journal of mental science, 195*(5), 440–447. <https://doi.org/10.1192/bjp.bp.108.060996>

Olivares-Olivares P.J., Olivares J., Macia D., Macia A., Montesinos L. (2016). Community versus clinical cognitive-behavioral intervention in young-adult Spanish population with generalized social phobia. *Terapia Psicologica, 34*(1), 23-30. <http://dx.doi.org/10.4067/S0718-48082016000100003>

Bautista, C. L., Ralston, A. L., Brock, R. L., & Hope, D. A. (2022). Peer coach support in internet-based cognitive behavioral therapy for college students with social anxiety disorder: efficacy and acceptability. *Cogent Psychology, 9*(1). <https://doi.org/10.1080/23311908.2022.2040160>

Kocovski, N. L., Fleming, J. E., Hawley, L. L., Huta, V., & Antony, M. M. (2013). Mindfulness and acceptance-based group therapy versus traditional cognitive behavioral group therapy for social anxiety disorder: a randomized controlled trial. *Behavioral research and therapy, 51*(12), 889–898. <https://doi.org/10.1016/j.brat.2013.10.007>

Kushner, M. G., Krueger, R. F., Wall, M. M., Maurer, E. W., Menk, J. S., & Menary, K. R. (2013). Modeling and treating internalizing psychopathology in a clinical trial: a latent variable structural equation modeling approach. *Psychological medicine, 43*(8), 1611–1623. <https://doi.org/10.1017/S0033291712002772>

Leichsenring, F., Salzer, S., Beutel, M. E., Herpertz, S., Hiller, W., Hoyer, J., Huesing, J., Joraschky, P., Nolting, B., Poehlmann, K., Ritter, V., Stangier, U., Strauss, B., Stuhldreher, N., Tefikow, S., Teismann, T., Willutzki, U., Wiltink, J., & Leibing, E. (2013). Psychodynamic therapy and cognitive-behavioral therapy in social anxiety disorder: a multicenter randomized controlled trial. *The American journal of psychiatry, 170*(7), 759–767. <https://doi.org/10.1176/appi.ajp.2013.12081125>

Herbert, J. D., Forman, E. M., Kaye, J. L., Gershkovich, M., Goetter, E., Yuen, E. K., Glassman, L., Goldstein, S., Hitchcock, P., Tronieri, J. S., Berkowitz, S., & Marando-Blanck, S. (2018). Randomized controlled trial of acceptance and commitment therapy versus traditional cognitive behavior therapy for social anxiety disorder: Symptomatic and behavioral outcomes. *Journal of Contextual Behavioral Science, 9*, 88–96. <https://doi.org/10.1016/j.jcbs.2018.07.008>

Schmidt, N. B., Buckner, J. D., Pusser, A., Woolaway-Bickel, K., Preston, J. L., & Norr, A. (2012). Randomized controlled trial of false safety behavior elimination therapy: a unified cognitive behavioral treatment for anxiety psychopathology. *Behavior therapy, 43*(3), 518–532. <https://doi.org/10.1016/j.beth.2012.02.004>

Zainal, N. H., Chan, W. W., Saxena, A. P., Taylor, C. B., & Newman, M. G. (2021). Pilot randomized trial of self-guided virtual reality exposure therapy for social anxiety disorder. *Behavioral research and therapy, 147*, 103984. <https://doi.org/10.1016/j.brat.2021.103984>

Alden, L. E., & Taylor, C. T. (2011). Relational treatment strategies increase social approach behaviors in patients with Generalized Social Anxiety Disorder. *Journal of anxiety disorders, 25*(3), 309–318. <https://doi.org/10.1016/j.janxdis.2010.10.003>

Borge, F. M., Hoffart, A., Sexton, H., Clark, D. M., Markowitz, J. C., & McManus, F. (2008). Residential cognitive therapy versus residential interpersonal therapy for social phobia: a randomized clinical trial. *Journal of anxiety disorders, 22*(6), 991–1010. <https://doi.org/10.1016/j.janxdis.2007.10.002>

Rubin, M., Muller, K., Hayhoe, M. M., & Telch, M. J. (2022). Attention guidance augmentation of virtual reality exposure therapy for social anxiety disorder: a pilot randomized controlled trial. *Cognitive behaviour therapy, 51*(5), 371–387. <https://doi.org/10.1080/16506073.2022.2053882>

Arai, H., Ishikawa, S., & Okawa, S. et al. (2023). Safety aid elimination as a brief, preventative intervention for social anxiety: A randomized controlled trial in university students. *Current Psychology, 42*, 20362–20373. <https://doi.org/10.1007/s12144-022-02981-8>

Koszycki, D., Benger, M., Shlik, J., & Bradwejn, J. (2007). Randomized trial of a meditation-based stress reduction program and cognitive behavior therapy in generalized social anxiety disorder. *Behavioral research and therapy, 45*(10), 2518–2526. <https://doi.org/10.1016/j.brat.2007.04.011>

Koszycki, D., Guérin, E., DiMillo, J., & Bradwejn, J. (2021). Randomized trial of cognitive behaviour group therapy and a mindfulness-based intervention for social anxiety disorder: Preliminary findings. *Clinical psychology & psychotherapy, 28*(1), 200–218. <https://doi.org/10.1002/cpp.2502>

Schmidt, N. B., Richey, J. A., Buckner, J. D., & Timpano, K. R. (2009). Attention training for generalized social anxiety disorder. *Journal of abnormal psychology, 118*(1), 5–14. https://doi.org/10.1037/a0013643

Teale Sapach, M. J. N., & Carleton, R. N. (2023). Self-compassion training for individuals with social anxiety disorder: a preliminary randomized controlled trial. *Cognitive behaviour therapy, 52*(1), 18–37. <https://doi.org/10.1080/16506073.2022.2130820>

Abramowitz, J. S., Moore, E. L., Braddock, A. E., & Harrington, D. L. (2009). Self-help cognitive-behavioral therapy with minimal therapist contact for social phobia: a controlled trial. *Journal of behavior therapy and experimental psychiatry, 40*(1), 98–105. <https://doi.org/10.1016/j.jbtep.2008.04.004>

Samantaray, N. N., Nath, B., Behera, N., Mishra, A., Singh, P., & Sudhir, P. (2021). Brief cognitive behavior group therapy for social anxiety among medical students: A randomized placebo-controlled trial. *Asian journal of psychiatry, 55*, 102526. <https://doi.org/10.1016/j.ajp.2020.102526>

Singh, P., & Samantaray, N. N. (2022). Brief Cognitive Behavioral Group Therapy and Verbal-Exposure-Augmented Cognitive Behavioral Therapy for Social Anxiety Disorder in University Students: A Randomized Controlled Feasibility Trial. *Indian journal of psychological medicine, 44*(6), 552–557. <https://doi.org/10.1177/02537176211026250>

Kan, C., Wang, Y., Hu, R., et al. (2025). Smartphone-based self-help virtual reality exposure therapy for college students’ social anxiety: A randomized controlled study. *Virtual Reality, 29*, Article 113. <https://doi.org/10.1007/s10055-025-01195-0>

Kocovski, N. L., Fleming, J. E., Blackie, R. A., MacKenzie, M. B., & Rose, A. L. (2019). Self-Help for Social Anxiety: Randomized Controlled Trial Comparing a Mindfulness and Acceptance-Based Approach With a Control Group. *Behavior therapy, 50*(4), 696–709. <https://doi.org/10.1016/j.beth.2018.10.007>

Titov, N., Andrews, G., Schwencke, G., Drobny, J., & Einstein, D. (2008). Shyness 1: distance treatment of social phobia over the Internet. *The Australian and New Zealand journal of psychiatry, 42*(7), 585–594. <https://doi.org/10.1080/00048670802119762>

Titov, N., Andrews, G., & Schwencke, G. (2008). Shyness 2: treating social phobia online: replication and extension. *The Australian and New Zealand journal of psychiatry, 42*(7), 595–605. <https://doi.org/10.1080/00048670802119820>

Cougle, J. R., Mueller, N. E., McDermott, K. A., Wilver, N. L., Carlton, C. N., & Okey, S. A. (2020). Text message safety behavior reduction for social anxiety: A randomized controlled trial. *Journal of consulting and clinical psychology, 88*(5), 445–454. <https://doi.org/10.1037/ccp0000494>

Stevenson, J., Mattiske, J. K., & Nixon, R. D. V. (2019). The effect of a brief online self-compassion versus cognitive restructuring intervention on trait social anxiety. *Behavioral research and therapy, 123*, 103492. <https://doi.org/10.1016/j.brat.2019.103492>

Khoramnia, S., Bavafa, A., Jaberghaderi, N., Parvizifard, A., Foroughi, A., Ahmadi, M., & Amiri, S. (2020). The effectiveness of acceptance and commitment therapy for social anxiety disorder: a randomized clinical trial. *Trends in psychiatry and psychotherapy, 42*(1), 30–38. <https://doi.org/10.1590/2237-6089-2019-0003>

Stangier, U., Schramm, E., Heidenreich, T., Berger, M., & Clark, D. M. (2011). Cognitive therapy vs interpersonal psychotherapy in social anxiety disorder: a randomized controlled trial. *Archives of general psychiatry, 68*(7), 692–700. <https://doi.org/10.1001/archgenpsychiatry.2011.67>

Maoz, K., Abend, R., Fox, N. A., Pine, D. S., & Bar-Haim, Y. (2013). Subliminal attention bias modification training in socially anxious individuals. *Frontiers in human neuroscience, 7*, 389. <https://doi.org/10.3389/fnhum.2013.00389>

Norton, A. R., & Abbott, M. J. (2016). The efficacy of imagery rescripting compared to cognitive restructuring for social anxiety disorder. *Journal of anxiety disorders, 40*, 18–28. <https://doi.org/10.1016/j.janxdis.2016.03.009>

Morvaridi, M., Mashhadi, A., & Shamloo, Z. S. (2019). The effectiveness of group emotional schema therapy on emotional regulation and social anxiety symptoms. *Journal of Cognitive Therapy, 12*(1), 16–24. <https://doi.org/10.1007/s41811-018-0037-6>

Beidel, D. C., Alfano, C. A., Kofler, M. J., Rao, P. A., Scharfstein, L., & Wong Sarver, N. (2014). The impact of social skills training for social anxiety disorder: a randomized controlled trial. *Journal of anxiety disorders, 28*(8), 908–918. <https://doi.org/10.1016/j.janxdis.2014.09.016>

Jazaieri, H., Goldin, P. R., & Gross, J. J. (2018). The role of working alliance in CBT and MBSR for social anxiety disorder. *Mindfulness, 9*, 1381–1389. <https://doi.org/10.1007/s12671-017-0877-9>

Mersch P.P.A. (1995). The treatment of social phobia: The differential effectiveness of exposure in vivo and an integration of exposure in vivo, rational emotive therapy and social skills training. *Behavioral Research and Therapy, 33*(3), 259-269. <http://dx.doi.org/10.1016/0005-7967(94)00038-L>

Andersson, G., Carlbring, P., Furmark, T., & S. O. F. I. E. Research Group (2012). Therapist experience and knowledge acquisition in internet-delivered CBT for social anxiety disorder: a randomized controlled trial. *PloS one, 7*(5), e37411. <https://doi.org/10.1371/journal.pone.0037411>

Titov, N., Andrews, G., Johnston, L., Robinson, E., & Spence, J. (2010). Transdiagnostic Internet treatment for anxiety disorders: A randomized controlled trial. *Behavioral research and therapy, 48*(9), 890–899. <https://doi.org/10.1016/j.brat.2010.05.014>

Wen, X., Gou, M., Chen, H., Kishimoto, T., Qian, M., Margraf, J., & Berger, T. (2024). The Efficacy of Web-Based Cognitive Behavioral Therapy With a Shame-Specific Intervention for Social Anxiety Disorder: Randomized Controlled Trial. *JMIR mental health, 11*, e50535. <https://doi.org/10.2196/50535>

Işık, M., Akbaş, P., & Özkan Şat, S. (2025). The impact of motivational interviewing on social anxiety, self-compassion, and dating violence among nursing students with social anxiety: A randomized controlled trial. *Current Psychology, 44*, 14738–14749. <https://doi.org/10.1007/s12144-025-08197-w>

Tillfors, M., Carlbring, P., Furmark, T., Lewenhaupt, S., Spak, M., Eriksson, A., Westling, B. E., & Andersson, G. (2008). Treating university students with social phobia and public speaking fears: Internet delivered self-help with or without live group exposure sessions. *Depression and anxiety, 25*(8), 708–717. <https://doi.org/10.1002/da.20416>

Rapee, R. M., Abbott, M. J., Baillie, A. J., & Gaston, J. E. (2007). Treatment of social phobia through pure self-help and therapist-augmented self-help. *The British journal of psychiatry : the journal of mental science, 191*, 246–252. <https://doi.org/10.1192/bjp.bp.106.028167>

Carlbring, P., Gunnarsdóttir, M., Hedensjö, L., Andersson, G., Ekselius, L., & Furmark, T. (2007). Treatment of social phobia: randomised trial of internet-delivered cognitive-behavioural therapy with telephone support. *The British journal of psychiatry : the journal of mental science, 190*, 123–128. <https://doi.org/10.1192/bjp.bp.105.020107>

Levin, M. E., Haeger, J. A., Pierce, B. G., & Twohig, M. P. (2017). Web-Based Acceptance and Commitment Therapy for Mental Health Problems in College Students: A Randomized Controlled Trial. *Behavior modification, 41*(1), 141–162. <https://doi.org/10.1177/0145445516659645>

Himle, J. A., Bybee, D., Steinberger, E., Laviolette, W. T., Weaver, A., Vlnka, S., Golenberg, Z., Levine, D. S., Heimberg, R. G., & O'Donnell, L. A. (2014). Work-related CBT versus vocational services as usual for unemployed persons with social anxiety disorder: A randomized controlled pilot trial. *Behavioral research and therapy, 63*, 169–176. <https://doi.org/10.1016/j.brat.2014.10.005>

Himle, J. A., LeBeau, R. T., Jester, J. M., Kilbourne, A. M., Weaver, A., Brydon, D. M., Tucker, K. M., Hamameh, N., Castriotta, N., & Craske, M. G. (2024). Work-Related Cognitive Behavioral Therapy for racially and economically diverse unemployed persons with social anxiety: A randomized clinical trial. *Journal of anxiety disorders, 104*, 102875. <https://doi.org/10.1016/j.janxdis.2024.102875>

Winter, H. R., Norton, A. R., & Wootton, B. M. (2025). Videoconferencing-delivered cognitive behavioural therapy for social anxiety disorder: a randomised controlled trial. Cognitive behaviour therapy, 1–17. *Advance online publication*. <https://doi.org/10.1080/16506073.2025.2540916>

Safir, M. P., Wallach, H. S., & Bar-Zvi, M. (2012). Virtual reality cognitive-behavior therapy for public speaking anxiety: one-year follow-up. *Behavior modification, 36*(2), 235–246. <https://doi.org/10.1177/0145445511429999>

Chard, I., Van Zalk, N., & Picinali, L. (2023). Virtual reality exposure therapy for reducing social anxiety in stuttering: A randomized controlled pilot trial. *Frontiers in digital health, 5*, 1061323. <https://doi.org/10.3389/fdgth.2023.1061323>

Ørskov, P. T., Runge, E., Sainte-Marie, T. T. H., Ernst, M. T., Clemmensen, L., Dalsgaard, C. H., Lichtenstein, M. B., & Bouchard, S. (2025). Virtual reality-based exposure with 360° video as part of cognitive behavioral therapy for social anxiety disorder: A three-arm randomized controlled trial. *Frontiers in Virtual Reality, 6*. <https://doi.org/10.3389/frvir.2025.1588181>

**Supplementary S5 –** Summary of Key Effect Modifiers in the Network Meta-Analysis

| **Study ID** | **Direct comparison Intervention** | **Baseline severity** | **Diagnostic method** | **Delivery mode** | **Number of sessions** | **Therapist qualification** |
| --- | --- | --- | --- | --- | --- | --- |
| Bouchard 2017 | CBT + Virtual Reality Exposure Therapy vs. CBT + Reality Exposure Therapy vs. Waitlist | Severe | DSM-5 | Individual face-to-face | 14 sessions | Graduate students |
| Gorinelli 2023 | Virtual Reality Acceptance and Commitment Therapy vs. Waiting List Control | Severe | Self-report | Individual face-to-face | 3 sessions | No in-person therapist |
| Olthuis 2014 | Telephone-delivered CBT vs. Waiting list control | Severe | DSM-IV | Online | 8 sessions | Registered psychologists and senior clinical psychology PhD students |
| Recabarren 2019 | Multidimensional stress prevention program vs. Wait-list control | Mild to Moderate | M.I.N.I. | Group face-to-face | 8 sessions | Trained clinical psychologists |
| Piet 2010 | Mindfulness-Based Cognitive Therapy vs. Group Cognitive-Behavioral Therapy | Moderate to Severe | DSM-IV | Group face-to-face | 8 sessions | Experienced therapist |
| Knijnik 2004 | Psychodynamic Group Therapy vs. Credible Placebo Control | Severe | DSM-IV | Group face-to-face | 12 sessions | Psychiatrist and psychotherapist |
| Amir 2012 | Interpretation Modification Program vs. Interpretation Control Condition | Severe | DSM-IV | Online | 6 sessions | Independent evaluators were trained graduate students and postdoctoral fellows |
| Nordmo 2015 | Internet-delivered CBT with an initial face-to-face psychoeducation session vs. Internet-delivered CBT alone vs. Waitlist | Moderate to Severe | DSM-IV | Mixed | 9 sessions | Clinical psychologists and clinical psychology students in their final year of study |
| Berger 2009 | Internet-based cognitive-behavioral treatment vs. Waiting-list control | Moderate to Severe | DSM-IV | Online | 10 sessions | Psychologists |
| Andersson 2006 | Internet-delivered CBT vs. Waiting list control | Severe | DSM-IV | Mixed | 9 sessions | Licensed clinical psychologists |
| Rapee 2013 | Cognitive-Behavioral Therapy + Attention Bias Modification Procedure vs. Cognitive-Behavioral Therapy + Placebo Training | Moderate to Severe | DSM-IV | Group face-to-face | 12 sessions | Clinical psychologists and graduate psychology students |
| Blanco 2010 | Cognitive Behavioral Group Therapy vs. Phenelzine vs. Combined vs. Pill Placebo | Severe | DSM-IV | Group face-to-face | 12 sessions | Masters- or doctoral-level therapists |
| Handley 2015 | Cognitive Behavioral Group Therapy for perfectionism vs. Waitlist control | Subclinical | M.I.N.I. | Group face-to-face | 8 sessions | Clinical Psychologist Registrar and Master of Clinical Psychology students |
| de Rutte 2025 | Active game-based Attention Bias Modification vs. Sham Control | Severe | Self-report | Online | 16 sessions | No therapist (Automated mobile game) |
| Gharraee 2018 | Compassion Focused Therapy vs. Waiting list | Severe | DSM-IV | Individual face-to-face | 12 sessions | Ph.D. student in clinical psychology |
| Ezenwaji 2021 | Group rational-emotive behavioral education intervention vs. No-contact control group | Severe | Self-report | Group face-to-face | 12 sessions | Researchers |
| Bunnell 2013 | Attention Training vs. Attention Control | Severe | DSM-IV | Online | 8 sessions | Senior doctoral students in clinical psychology |
| Krafft 2020 | Acceptance and Commitment Therapy self-help book vs. traditional Cognitive Behavior Therapy self-help book | Moderate to Severe | Self-report | Online | 8 sessions | No therapist |
| Amir 2009 | Attention Modification Program vs. Attention Control Condition | Severe | DSM-IV | Online | 8 sessions | No therapist |
| Vally 2024 | Internet-delivered Acceptance and Commitment Therapy vs. Waitlist control | Mild to Moderate | Self-report | Online | 4 sessions | No therapist |
| Lazarov 2017 | Cognitive-Behavioral Group Therapy + Attention Bias Modification vs. Cognitive-Behavioral Group Therapy + Placebo | Severe | DSM-IV | Mixed | 18 sessions | Senior clinical psychologist and co-therapist |
| Bjornsson 2011 | Cognitive-Behavioral Group Therapy vs. Group Psychotherapy | Severe | DSM-IV | Group face-to-face | 8 sessions | Advanced clinical psychology graduate students and a licensed clinical psychologist |
| Mueller 2023 | Building Closer Friendships (BCF) internet-based intervention vs. Waitlist control | Severe | DSM-5 | Online | 4 sessions | No therapist |
| Clark 2003 | Cognitive Therapy vs. Fluoxetine + Self-exposure vs. Placebo + Self-exposure | Severe | DSM-IV | Individual face-to-face | 16 sessions | Clinical psychologists |
| D'El Rey 2008 | Cognitive-behavioral group treatment vs. Waiting list control | Severe | DSM-IV | Group face-to-face | 12 sessions | Psychologist with experience in cognitive-behavioral treatment model for social phobia |
| Dagöö 2014 | Smartphone and computer delivered Cognitive Behavior Therapy vs. Smartphone and computer delivered Interpersonal Psychotherapy | Severe | DSM-IV | Online | 9 sessions | Clinical psychology MSc students in their final semester |
| Cottraux 2000 | Cognitive Behavior Therapy vs. Supportive Therapy | Severe | DSM-IV | Mixed | 14 sessions | Psychiatrists or clinical psychologists trained |
| Heimberg 1998 | Cognitive Behavioral Group Therapy vs. Phenelzine vs. Pill placebo vs. Educational-supportive group therapy | Severe | DSM-III | Group face-to-face | 12 sessions | Psychologist and cotherapist |
| Yoshinaga 2016 | Cognitive Behavioral Therapy + Usual Care vs. Usual Care | Severe | DSM-IV | Individual face-to-face | 16 sessions | Clinical psychologists, psychiatrist, nurse, or psychiatric social worker |
| Kählke 2023 | Internet- and mobile-based intervention vs. Waitlist control | Moderate to Severe | DSM-IV | Online | 10 sessions | No therapist |
| Clark 2006 | Cognitive Therapy vs. Exposure plus Applied Relaxation vs. Wait-list | Severe | DSM-IV | Individual face-to-face | 14 sessions | Clinical psychologists |
| Boettcher 2014 | Attention Bias Modification vs. Internet-Based Cognitive-Behavioral Self-Help | Moderate to Severe | DSM-IV | Online | 11 sessions | MSc clinical psychology students |
| Borgeat 2009 | Self-Focused Exposure Therapy vs. Standard Cognitive-Behavioral Therapy | Severe | DSM-IV | Group face-to-face | 8 sessions | Psychiatrists and clinical psychologists |
| Powell 2020 | Self-guided internet intervention vs. Waiting list control | Mild to Moderate | Self-report | Online | 6 sessions | No therapist |
| Abeditehrani 2024 | Cognitive Behavioral Group Therapy vs. Psychodrama vs. Cognitive Behavioral Psychodrama Therapy vs. Waitlist control | Severe | DSM-IV | Group face-to-face | 12 sessions | Therapists trained and experienced in both CBGT and PD |
| Bell 2012 | Computerised cognitive behaviour therapy vs. Wait list control | Severe | DSM-IV | Online | 6 sessions | No therapist |
| Kählke 2019 | Unsupervised Internet-based Self-Help Intervention Based on the Cognitive Behavioral Therapy Model vs. Waitlist control | Moderate to Severe | DSM-IV | Online | 10 sessions | No therapist |
| de Oliveira 2012 | Trial-based thought record vs. Conventional cognitive therapy | Severe | DSM-IV | Individual face-to-face | 12 sessions | Psychologists who had attended a two-year cognitive therapy specialization course and had at least 1 year of experience in private practice |
| Garcia-Lopez 2006 | Cognitive Behavioral Group Therapy for Adolescents vs. Social Effectiveness Therapy for Adolescents-Spanish version vs. Intervención en Adolescentes con Fobia Social vs. Control | Severe | DSM-IV | Mixed | Range (12 to 29 sessions) | Unreported in this specific follow-up study |
| Enock 2014 | Attention bias modification training vs. Control training vs. Waitlist | Moderate to Severe | Self-report | Online | 83 sessions | No therapist |
| McCall 2018 | Web-based Cognitive Behavioral Therapy vs. Wait-list control | Moderate to Severe | Self-report | Online | 7 sessions | No therapist |
| Mall 2011 | DVD-based self-help program vs. Wait-list control | Subclinical | SCID-I and SCID-II | Mixed | 8 sessions | Master's-level student in psychology |
| Mattick 1989 | Exposure vs. Cognitive restructuring without exposure vs. Combined vs. Wait-list control | Severe | DSM-III | Group face-to-face | 6 sessions | Clinical psychologists |
| Goldin 2016 | Cognitive-behavioral group therapy vs. Mindfulness-based stress reduction vs. Waitlist | Severe | DSM-IV | Group face-to-face | 12 sessions | Doctoral clinical psychologists |
| Furmark 2009 | Internet-delivered cognitive-behavioural therapy vs. Pure bibliotherapy vs. Bibliotherapy with discussion group vs. Internet-delivered applied relaxation vs. Waiting-list control | Severe | DSM-IV | Online | 9 sessions | Licensed clinical psychologists and clinical psychology students |
| Nilsson 2012 | Imagery rescripting vs. Reading task | Severe | DSM-IV | Individual face-to-face | 1 session | Unreported |
| Hyett 2018 | Single-session group imagery rescripting vs. Single-session group verbal restructuring vs. Waitlist control | Severe | DSM-IV | Group face-to-face | 1 session | Clinical psychologist registrar and a clinical psychologist in advanced training |
| McEvoy 2020 | Imagery-enhanced group cognitive behavior therapy vs. Verbally-based group cognitive behavior therapy | Severe | DSM-5 | Group face-to-face | 12 sessions | Masters or doctoral level clinical psychologists |
| Sigurðardóttir 2022 | Web-based cognitive-behavioural therap + online group psychoeducation vs. Web-based cognitive-behavioural therapT + online PMR group sessions vs. Web-based cognitive-behavioural therap alone | Moderate to Severe | Self-report | Mixed | 16 sessions | Therapist-led for group sessions |
| Johansson 2017 | Internet-based affect-focused psychodynamic therapy vs. Wait-list control | Severe | DSM-IV | Online | 9 sessions | Master's level students in their final year of a 5-year clinical psychologist program |
| Carlbring 2012 | Internet-delivered attention bias modification vs. Placebo training | Severe | DSM-IV | Online | 8 sessions | No therapist |
| Thew 2022 | Internet-delivered cognitive therapy vs. Waitlist control | Severe | DSM-5 | Online | 14 sessions | Clinical psychologists |
| Tulbure 2015 | Internet-based cognitive-behavioral therapy vs. Wait-list control group | Subclinical | DSM-IV | Online | 9 sessions | Online psychologists |
| Cougle 2020 | Interpretation bias modification vs. Progressive muscle relaxation | Severe | DSM-IV | Online | 8 sessions | No therapist |
| Lin 2019 | Arousal feedback-based exposure therapy vs. Waitlist control | Moderate to Severe | Self-report | Mixed | 4 sessions | No therapist |
| Salaberría 1998 | Self-exposure in vivo vs. Self-exposure in vivo with cognitive therapy vs. Waiting-list control | Severe | DSM-III | Group face-to-face | 8 sessions | Clinical psychologist |
| Furmark 2009 | Internet-delivered cognitive-behavioural therapy vs. Pure bibliotherapy vs. Bibliotherapy with discussion group vs. Internet-delivered applied relaxation vs. Waiting-list control | Severe | DSM-IV | Online | 9 sessions | Licensed clinical psychologists and clinical psychology students |
| Olivares 2016 | Social Effectiveness Therapy vs. Cognitive Behavioral Group Therapy vs. Waiting-list control group | Severe | DSM-IV | Mixed | Range (12 to 29 sessions) | Clinical psychologists |
| Bautista 2022 | Internet-based cognitive-behavioural therapy with peer coach support vs. Wait-list control | Moderate to Severe | Self-report | Mixed | 6 sessions | Undergraduate students |
| Kocovski 2013 | Mindfulness and acceptance-based group therapy vs. Cognitive behavioral group therapy vs. Waitlist control | Severe | DSM-IV | Group face-to-face | 12 sessions | Experienced therapists |
| Kushner 2013 | Cognitive-behavioral therapy vs. Progressive muscle relaxation training | Moderate to Severe | DSM-IV | Unreported | 6 sessions | Unreported |
| Leichsenring 2013 | Cognitive-Behavioral Therapy vs. Psychodynamic Therapy vs. Waiting list | Severe | DSM-IV | Individual face-to-face | Up to 25 sessions | Clinical psychologists or physicians |
| Herbert 2018 | Acceptance and Commitment Therapy vs. Traditional Cognitive Behavior Therapy | Severe | DSM-IV | Individual face-to-face | 12 sessions | Clinical psychology doctoral or master's students |
| Schmidt 2012 | False safety behavior elimination therapy vs. Wait-list control | Severe | DSM-IV | Group face-to-face | Unreported | Relatively inexperienced clinicians / Psychologists and master's-level therapists |
| Zainal 2021 | Self-guided virtual reality exposure therapy vs. Waitlist | Severe | DSM-5 | Mixed | Range (4 to 8 sessions) | No human therapist |
| Alden 2011 | Integrated interpersonal cognitive-behavioral group treatment vs. Wait list condition | Severe | DSM-IV | Group face-to-face | 12 sessions | Senior doctoral students in psychology, a psychology intern, and a psychiatrist |
| Borge 2008 | Residential cognitive therapy vs. Residential interpersonal therapy | Severe | DSM-IV | Mixed | 50 sessions | Clinical psychologists, a clinical social worker, and a psychiatrist resident |
| Rubin 2022 | Virtual reality exposure therapy + attention guidance training vs. Standard virtual reality exposure therapy | Severe | DSM-5 | Individual face-to-face | 2 sessions | Graduate student clinician |
| Arai 2023 | Safety Aid Elimination Intervention vs. Health Education and Adaptive Living control group | Subclinical | Self-report | Group face-to-face | 1 session | Clinical psychologist |
| Koszycki 2007 | Mindfulness-based stress reduction vs. Cognitive behavioral group therapy | Moderate to Severe | DSM-IV | Group face-to-face | Range (8 to 12 sessions) | Experienced therapist |
| Koszycki 2020 | Mindfulness-based intervention for SAD vs. Cognitive behaviour group therapy | Moderate to Severe | DSM-5 | Group face-to-face | 12 sessions | Master's level clinical social workers, doctoral-level psychotherapists, senior doctoral-level clinical psychology students, and doctoral-level psychologists |
| Schmidt 2009 | Attention Training vs. Placebo control | Severe | DSM-IV | Mixed | 8 sessions | No therapist |
| Teale Sapach 2022 | Self-guided self-compassion training vs. Self-guided applied relaxation training vs. Waitlist control | Severe | DSM-5 | Online | 6 sessions | No therapist |
| Abramowitz 2009 | Self-directed cognitive-behavioral treatment with minimal therapist contact vs. Wait-list | Moderate to Severe | DSM-IV | Mixed | 8 sessions | Unreported |
| Samantaray 2021 | Brief cognitive behavior group therapy vs. Psychoeducational-supportive therapy | Moderate to Severe | DSM-IV | Group face-to-face | 6 sessions | Clinical psychologist |
| Singh 2021 | Brief cognitive-behavioral group therapy vs. Verbal exposure augmented cognitive behavioral therapy | Moderate to Severe | DSM-5 | Group face-to-face | 6 sessions | Registered clinical psychologist |
| Kan 2025 | Smartphone-based self-help virtual reality exposure therapy vs. Waitlist control group | Severe | Self-report | Mixed | 14 sessions | No therapist |
| Kocovski 2018 | Mindfulness and acceptance-based self-help book vs. Wait-list control condition | Severe | Self-report | Mixed | 8 sessions | No therapist |
| Titov 2008a | Clinician-assisted computerized cognitive behavioural therapy vs. Waitlist control | Severe | DSM-IV | Online | 6 sessions | Clinical psychologist |
| Titov 2008b | Clinician-assisted computerized cognitive behavioural therapy vs. Waitlist control | Severe | DSM-IV | Online | 6 sessions | Clinical psychologist |
| Cougle 2020 | Text message safety behavior reduction vs. Text message self-monitoring control | Severe | DSM-5 | Online | 8 sessions | No therapist |
| Stevenson 2019 | Online self-compassion vs. Online cognitive restructuring | Moderate to Severe | DSM-5 | Online | 14 sessions | No therapist |
| Khoramnia 2020 | Acceptance and commitment therapy vs. Waiting list control | Severe | DSM-IV | Face-to-face | 12 sessions | Unreported |
| Stangier 2011 | Cognitive Therapy vs. Interpersonal Psychotherapy vs. Waiting-list control | Severe | DSM-IV | Individual face-to-face | 16 sessions | Clinical psychologists and psychiatrists |
| Maoz 2013 | Subliminal attention bias modification vs. Placebo control | Moderate to Severe | Self-report | Online | 4 sessions | No therapist |
| Norton 2016 | Imagery rescripting vs. Cognitive restructuring vs. Control procedure | Severe | DSM-IV | Individual face-to-face | 1 session | Registered psychologist and postgraduate clinical psychology student |
| Morvaridi 2018 | Group Emotional Schema Therapy vs. Waiting list control | Severe | Self-report | Group face-to-face | 10 sessions | Unreported |
| Beidel 2014 | Exposure therapy vs. Social Effectiveness Therapy vs. Wait list control | Severe | DSM-IV | Mixed | 24 sessions | Doctoral level psychologists or doctoral students in clinical psychology |
| Jazaieri 2018 | Cognitive behavioral therapy vs. Mindfulness-based stress reduction | Severe | DSM-IV | Group face-to-face | 12 sessions | PhD-level clinical psychologists |
| Mersch 1995 | Exposure in vivo vs. Integrated Treatment vs. Waiting-list control | Severe | DSM-III | Individual face-to-face | 14 sessions | Advanced Mental Health Sciences students |
| Andersson 2012 | Guided Internet-delivered cognitive behavior therapy vs. Moderated online discussion group | Severe | DSM-IV | Online | 9 sessions | Psychology students at MSc level |
| Titov 2010 | Transdiagnostic Internet-based cognitive behavioural treatment vs. Waitlist control group | Severe | DSM-IV | Online | 6 sessions | Clinical psychologist |
| Wen 2024 | Conventional Online Cognitive Behavioral Therapy vs. Waiting group | Severe | DSM-IV | Online | 8 sessions | Unreported |
| Işık 2025 | Motivational interviewing vs. Control group | Subclinical | Self-report | Unreported | 5 sessions | Unreported |
| Tillfors 2008 | Internet-delivered Cognitive behavioral therapy combined with group exposure vs. Internet-delivered Cognitive behavioral therapy alone | Severe | DSM-IV | Mixed | 9 sessions | Licensed clinical psychologists and clinical psychology students in their last semester |
| Rapee 2007 | Standard group treatment vs. 'Pure' self-help vs. Self-help augmented by therapist assistance vs. Waiting list | Severe | DSM-IV | Mixed | 10 sessions | Graduate psychology students |
| Carlbring 2007 | Internet-delivered cognitive-behavioural therapy with telephone support vs. Waiting-list control group | Severe | DSM-IV | Online | 9 sessions | Master's degree psychology students |
| Levin 2016 | Web-based Acceptance and Commitment Therapy vs. Waitlist condition | Moderate to Severe | Self-report | Online | 6 sessions | No therapist |
| Himle 2014 | Work-related cognitive-behavioral therapy + Vocational services as usual vs. Vocational services as usual alone | Severe | DSM-IV | Group face-to-face | 8 sessions | Vocational services employees |
| Himle 2024 | Work-Related Cognitive Behavioral Therapy + Vocational services as usual vs. Vocational services as usual alone | Severe | DSM-5 | Group face-to-face | 8 sessions | Vocational service professionals |
| Winter 2025 | Videoconferencing-delivered cognitive behaviour therapy vs. Waitlist control | Moderate to Severe | DSM-5 | Online | 8 sessions | Provisionally registered or fully registered psychologists |
| Safir 2012 | Virtual reality cognitive-behavioral therapy vs. Cognitive-behavior therapy vs. Wait-list control | Subclinical | Self-report | Individual face-to-face | 12 sessions | Unreported |
| Chard 2023 | Virtual reality exposure therapy vs. Waitlist | Moderate to Severe | Self-report | Mixed | 3 sessions | No therapist |
| Ørskov 2025 | cognitive-behavioral therapy with VR-based exposure vs. cognitive-behavioral therapy with in vivo exposure vs. VR relaxation | Severe | ICD-10 | Individual face-to-face | 10 sessions | Trained psychologists |

**Supplementary S6 –** Risk of bias table of included studies


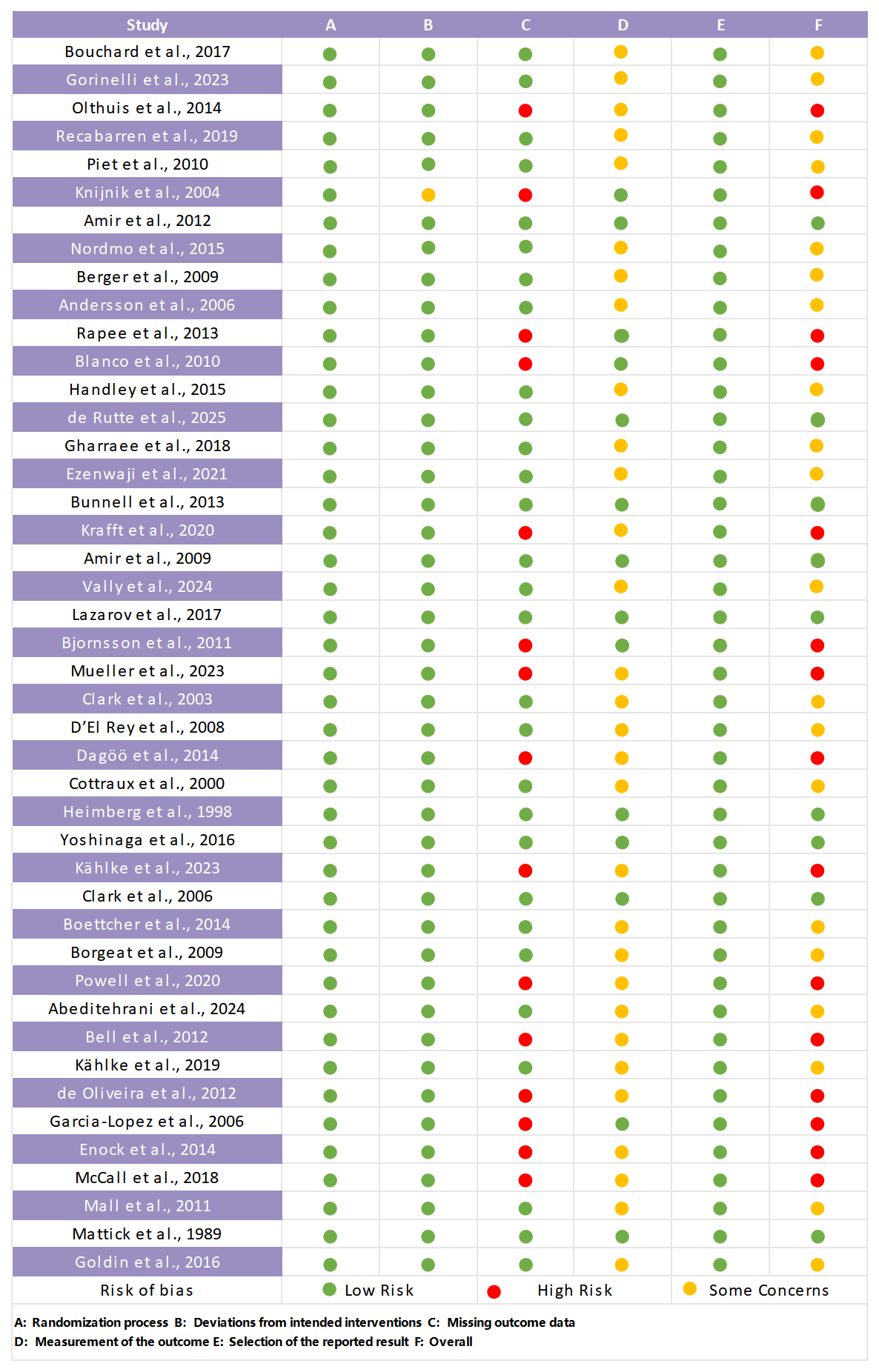


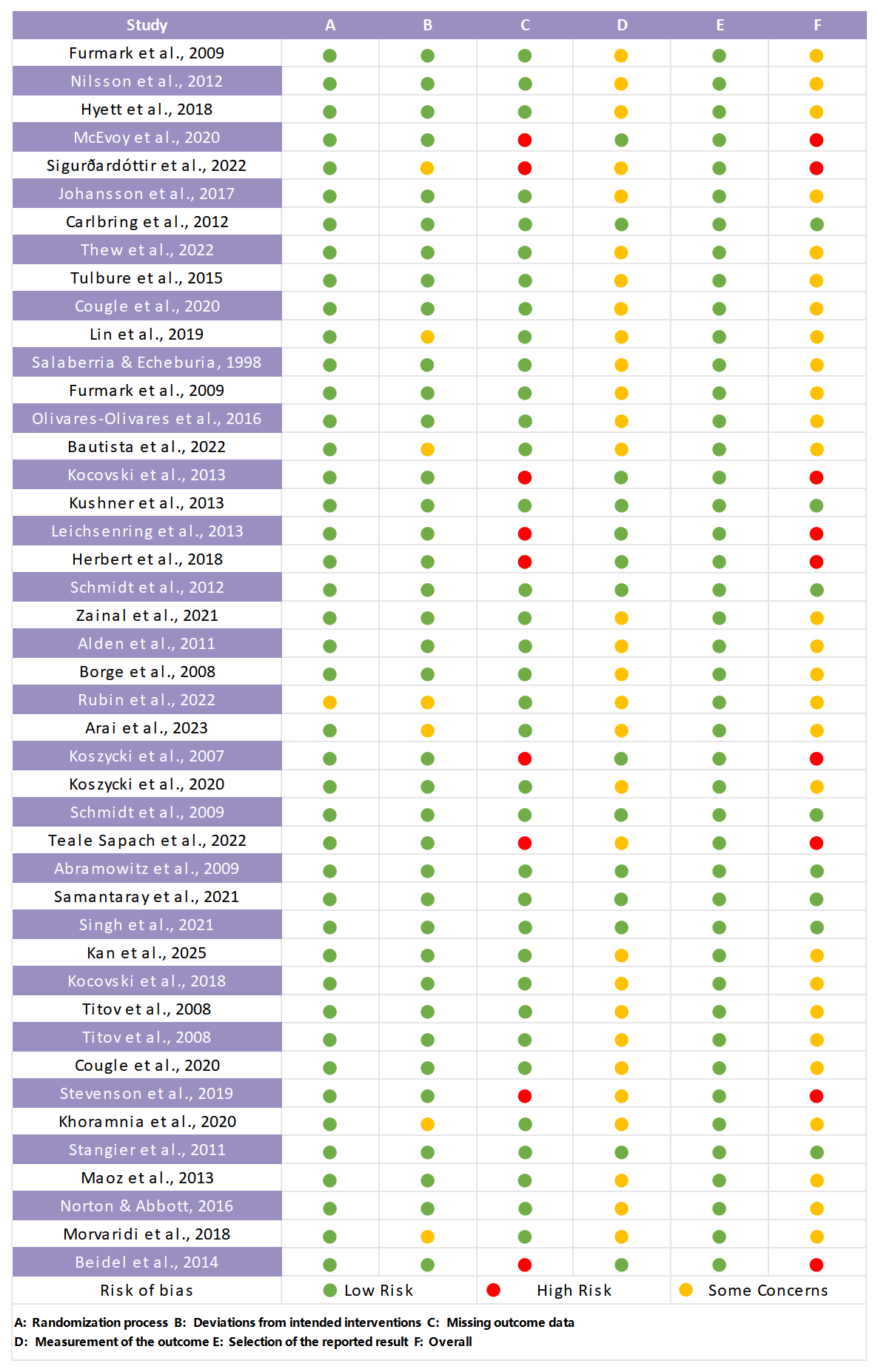


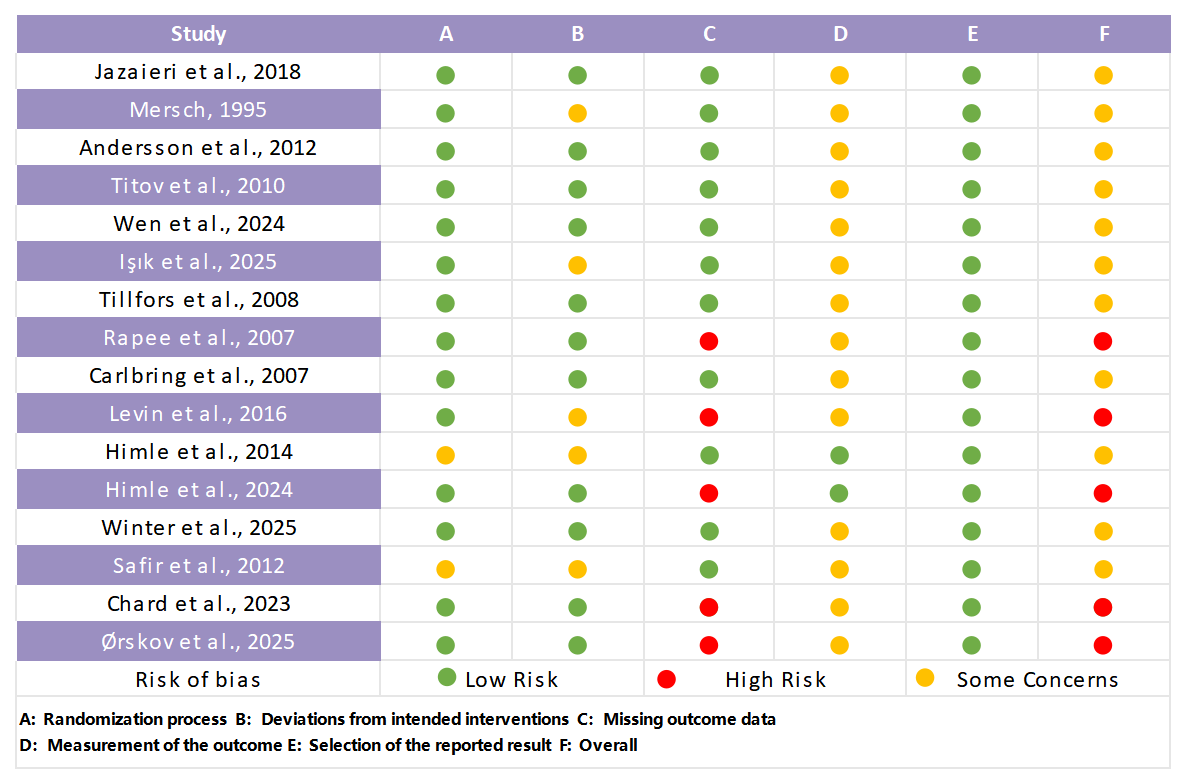


**Supplementary S7 –** Inconsistency Test

**Supplementary S7.1 –** Loop Inconsistency Test

| **Social Anxiety** |  |  |
| --- | --- | --- |
| Type | P value | Loop Heterogeneity (τ²) |
| CON-VRET-RT | 0.41 | 0.20 |
| VRET-RT-AT | 0.32 | 0.00 |
| CON-RT-AT | 0.16 | 0.06 |
| CON-RT-READ | 0.06 | 0.00 |
| CON-RT-CT | 0.42 | 0.26 |
| CON-VRET-AT | 0.57 | 0.24 |
| CT-PT-READ | 0.15 | 0.02 |
| CBT-PT-READ | 0.10 | 0.41 |
| CON-CT-PT | 0.51 | 0.00 |
| VRET-SIS-CT | 0.42 | 0.04 |
| VRET-CBT-RT | 0.27 | 0.40 |
| CON-SIS-CT | 0.63 | 0.00 |
| RT-PT-READ | 0.36 | 0.12 |
| CON-CBT-RT | 0.11 | 0.01 |
| VRET-CBT-CT | 0.42 | 0.00 |
| CON-CBT-AT | 0.40 | 0.16 |
| CON-CBT-PT | 0.23 | 0.15 |
| CON-CBT-CT | 0.38 | 0.15 |
| CON-CBT-READ | 0.56 | 0.15 |
| CON-VRET-CT | 0.84 | 0.40 |
| CON-VRET-SIS | 0.83 | 0.34 |
| RT-CT-READ | 0.71 | 0.00 |
| CON-CT-READ | 0.83 | 0.27 |
| VRET-CBT-AT | 0.82 | 0.09 |
| CBT-CT-PT | 0.59 | 0.02 |
| CON-CBT-SIS | 0.75 | 0.16 |
| CBT-CT-READ | 0.72 | 0.00 |
| VRET-RT-CT | 0.89 | 0.00 |
| CON-VRET-CBT | 0.82 | 0.16 |
| CBT-RT-AT | 0.88 | 0.08 |
| RT-CT-PT | 0.89 | 0.00 |
| CBT-RT-PT | 0.91 | 0.03 |
| CON-RT-PT | 0.96 | 0.29 |
| CBT-RT-READ | 0.92 | 0.05 |
| CBT-RT-CT | 0.91 | 0.04 |
| CON-CBT-ACT | 0.94 | 0.16 |
| CON-PT-READ | 0.97 | 0.30 |
| VRET-CBT-SIS | 0.97 | 0.07 |
| CBT-SIS-CT | 0.99 | 0.07 |

**Notes: CON:**Control group **VRET:**Virtual Reality Exposure Therapy **CBT:**Cognitive behavioral therapy **ACT:**Acceptance and Commitment Therapy **RT:**Relaxation therapy **SIS:**Social and Interpersonal Skills **CT**:Combination therapy **PT:**Psychotherapy **AT:**Attention training **READ:**Reading therapy

**Supplementary S7.2 –** Global Inconsistency Test

| **Social Anxiety** | |
| --- | --- |
|  |  |
| Type | |
| Chi2 | 25.70 |
| Pro ＞ Chi2 | 0.8178 |

**Supplementary S7.3 –** Local Inconsistency Test

| **Social Anxiety** |  |
| --- | --- |
| Type | P value |
| A B | 0.98 |
| A C | 0.96 |
| A D | 0.93 |
| A E | 0.07 |
| A F | 0.66 |
| A G | 0.28 |
| A H | 0.11 |
| A I | 0.23 |
| A J | 0.82 |
| B C | 0.63 |
| B E | 0.38 |
| B F | 0.95 |
| B G | 0.67 |
| B I | 0.53 |
| C D | 0.93 |
| C E | 0.47 |
| C F | 0.73 |
| C G | 0.37 |
| C H | 0.12 |
| C I | 0.43 |
| C J | 0.37 |
| E G | 0.82 |
| E H | 0.90 |
| E I | 0.75 |
| E J | 0.59 |
| F G | 0.72 |
| G H | 0.66 |
| G J | 0.89 |
| H J | 0.42 |

**Notes: A:**CON **B:**VRET **C:**CBT **D:**ACT **E:**RT **F:**SIS **G:**CT **H:**PT **I:**AT **J:**READ

**Supplementary S8 –** League table for adult social anxiety

| CON | -0.71(-0.96,-0.46) | -0.80 (-0.92,-0.68) | -0.34(-0.69,0.01) | -0.47(-0.71,-0.24) | -0.68(-1.00,-0.35) | -0.74(-1.00,-0.47) | -0.73 (-0.92,-0.54) | -0.41(-0.67,-0.14) | -0.67(-1.02,-0.32) |
| --- | --- | --- | --- | --- | --- | --- | --- | --- | --- |
| 0.71 (0.46,0.96) | VRET | -0.09 (-0.34,0.16) | 0.37 (-0.05,0.80) | 0.24 (-0.08,0.56) | 0.04 (-0.34,0.41) | -0.02 (-0.37,0.32) | -0.01 (-0.31,0.28) | 0.31 (-0.04,0.65) | 0.04 (-0.38,0.46) |
| 0.80 (0.68,0.92) | 0.09 (-0.16,0.34) | CBT | 0.46 (0.10,0.82) | 0.33 (0.10,0.55) | 0.12 (-0.19,0.44) | 0.07 (-0.20,0.33) | 0.07 (-0.11,0.26) | 0.40 (0.13,0.66) | 0.13 (-0.22,0.48) |
| 0.34 (-0.01,0.69) | -0.37 (-0.80,0.05) | -0.46 (-0.82,-0.10) | ACT | -0.13 (-0.55,0.29) | -0.34 (-0.81,0.14) | -0.39 (-0.83,0.04) | -0.39 (-0.78,0.01) | -0.06 (-0.50,0.37) | -0.33 (-0.82,0.16) |
| 0.47 (0.24,0.71) | -0.24 (-0.56,0.08) | -0.33 (-0.55,-0.10) | 0.13 (-0.29,0.55) | RT | -0.20 (-0.59,0.18) | -0.26 (-0.59,0.07) | -0.25 (-0.53,0.03) | 0.07 (-0.26,0.40) | -0.20 (-0.60,0.20) |
| 0.68 (0.35,1.00) | -0.04 (-0.41,0.34) | -0.12 (-0.44,0.19) | 0.34 (-0.14,0.81) | 0.20 (-0.18,0.59) | SIS | -0.06 (-0.45,0.34) | -0.05 (-0.41,0.31) | 0.27 (-0.13,0.68) | 0.01 (-0.46,0.47) |
| 0.74 (0.47,1.00) | 0.02 (-0.32,0.37) | -0.07 (-0.33,0.20) | 0.39 (-0.04,0.83) | 0.26 (-0.07,0.59) | 0.06 (-0.34,0.45) | CT | 0.01 (-0.29,0.31) | 0.33 (-0.03,0.69) | 0.07 (-0.34,0.47) |
| 0.73 (0.54,0.92) | 0.01 (-0.28,0.31) | -0.07 (-0.26,0.11) | 0.39 (-0.01,0.78) | 0.25 (-0.03,0.53) | 0.05 (-0.31,0.41) | -0.01 (-0.31,0.29) | PT | 0.32 (0.01,0.64) | 0.06 (-0.32,0.43) |
| 0.41 (0.14,0.67) | -0.31 (-0.65,0.04) | -0.40 (-0.66,-0.13) | 0.06 (-0.37,0.50) | -0.07 (-0.40,0.26) | -0.27 (-0.68,0.13) | -0.33 (-0.69,0.03) | -0.32 (-0.64,-0.01) | AT | -0.26 (-0.69,0.17) |
| 0.67 (0.32,1.02) | -0.04 (-0.46,0.38) | -0.13 (-0.48,0.22) | 0.33 (-0.16,0.82) | 0.20 (-0.20,0.60) | -0.01 (-0.47,0.46) | -0.07 (-0.47,0.34) | -0.06 (-0.43,0.32) | 0.26 (-0.17,0.69) | READ |

**Notes: CON:**Control group **VRET:**Virtual Reality Exposure Therapy **CBT:**Cognitive behavioral therapy **ACT:**Acceptance and Commitment Therapy **RT:**Relaxation therapy **SIS:**Social and Interpersonal Skills **CT**:Combination therapy **PT:**Psychotherapy **AT:**Attention training **READ:**Reading therapy

**Supplementary S9** – Surface Under the Cumulative Ranking curve Score and Ranking

| **Social Anxiety** | | | |
| --- | --- | --- | --- |
| Treatment | SUCRA | PrBest | Mean Rank |
| CON | 0.3 | 0 | 10 |
| VRET | 68.2 | 13.7 | 3.9 |
| CBT | 86.4 | 28.6 | 2.2 |
| ACT | 21.6 | 0.4 | 8.1 |
| RT | 31.8 | 0.1 | 7.1 |
| SIS | 62.5 | 13.8 | 4.4 |
| CT | 71.7 | 18.5 | 3.5 |
| PT | 70.7 | 9.8 | 3.6 |
| AT | 25.2 | 0.1 | 7.7 |
| READ | 61.5 | 15.1 | 4.5 |

**Notes: CON:**Control group **VRET:**Virtual Reality Exposure Therapy **CBT:**Cognitive behavioral therapy **ACT:**Acceptance and Commitment Therapy **RT:**Relaxation therapy **SIS:**Social and Interpersonal Skills **CT**:Combination therapy **PT:**Psychotherapy **AT:**Attention training **READ:**Reading therapy

**Supplementary S10 –** **Ranking Probability for Social anxiety**

| **Rank** | **Intervention** | | | | | | | |  |  |
| --- | --- | --- | --- | --- | --- | --- | --- | --- | --- | --- |
|  | **CON** | **VRET** | **CBT** | **ACT** | **RT** | **SIS** | **CT** | **PT** | **AT** | **READ** |
| Best | 0.0 | 13.7 | 28.6 | 0.4 | 0.1 | 13.8 | 18.5 | 9.8 | 0.1 | 15.1 |
| 2nd | 0.0 | 13.3 | 35.6 | 0.3 | 0.3 | 9.7 | 15.2 | 16.0 | 0.2 | 9.5 |
| 3rd | 0.0 | 15.3 | 23.8 | 0.8 | 0.9 | 11.7 | 15.9 | 21.1 | 0.4 | 10.3 |
| 4th | 0.0 | 18.2 | 9.3 | 1.5 | 2.4 | 13.7 | 18.1 | 22.8 | 1.3 | 12.8 |
| 5th | 0.0 | 19.0 | 2.5 | 2.9 | 6.2 | 17.3 | 16.1 | 18.1 | 3.3 | 14.6 |
| 6th | 0.0 | 13.8 | 0.3 | 6.3 | 14.6 | 17.7 | 10.7 | 9.7 | 8.6 | 18.4 |
| 7th | 0.0 | 5.3 | 0.0 | 13.7 | 35.1 | 9.3 | 3.9 | 2.0 | 20.4 | 10.3 |
| 8th | 0.0 | 1.3 | 0.0 | 21.7 | 28.1 | 5.1 | 1.3 | 0.5 | 36 | 6.2 |
| 9th | 2.5 | 0.3 | 0.0 | 50.1 | 12.4 | 1.8 | 0.4 | 0.0 | 29.7 | 2.8 |
| Worst | 97.5 | 0.0 | 0.0 | 2.4 | 0.0 | 0.0 | 0.0 | 0.0 | 0.1 | 0.0 |

**Notes: CON:**Control group **VRET:**Virtual Reality Exposure Therapy **CBT:**Cognitive behavioral therapy **ACT:**Acceptance and Commitment Therapy **RT:**Relaxation therapy **SIS:**Social and Interpersonal Skills **CT**:Combination therapy **PT:**Psychotherapy **AT:**Attention training **READ:**Reading therapy

**Supplementary S11** – Assessment of Publication Bias and Small-Study Effects

**Supplementary S11.1 –** Comparison-adjusted funnel plot


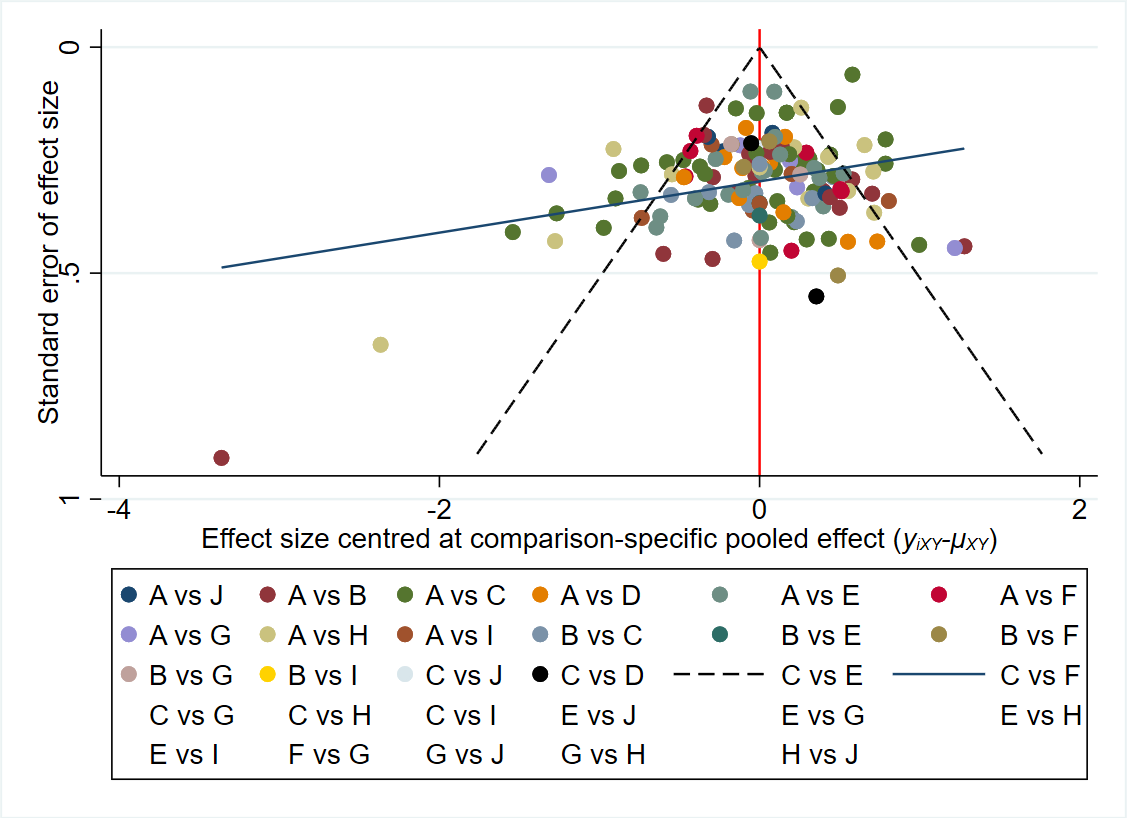


**Notes: A:**CON **B:**VRET **C:**CBT **D:**ACT **E:**RT **F:**SIS **G:**CT **H:**PT **I:**AT **J:**READ

**Notes: CON:**Control group **VRET:**Virtual Reality Exposure Therapy **CBT:**Cognitive behavioral therapy **ACT:**Acceptance and Commitment Therapy **RT:**Relaxation therapy **SIS:**Social and Interpersonal Skills **CT**:Combination therapy **PT:**Psychotherapy **AT:**Attention training **READ:**Reading therapy

**Supplementary S11.2** –Egger's regression test and trim-and-fill analysis results for eligible comparisons

| **Comparison** | **Egger's Intercept (Bias)** | **95% CI for Intercept** | **t-value** | **p-value** |
| --- | --- | --- | --- | --- |
| CBT vs CON | −1.72 | −3.36 to -0.08 | −2.12 | 0.041 |
| PT vs CBT | −0.53 | −1.96 to 0.90 | −0.79 | 0.442 |
| PT vs CON | −2.52 | −6.88 to 1.84 | −1.29 | 0.227 |
| RT vs CBT | 2.54 | 0.17 to 4.90 | 2.43 | 0.038 |

**Supplementary S12 –** **Subgroup analysis**

**Supplementary S12.1 –** **Network evidence graph for developed country**


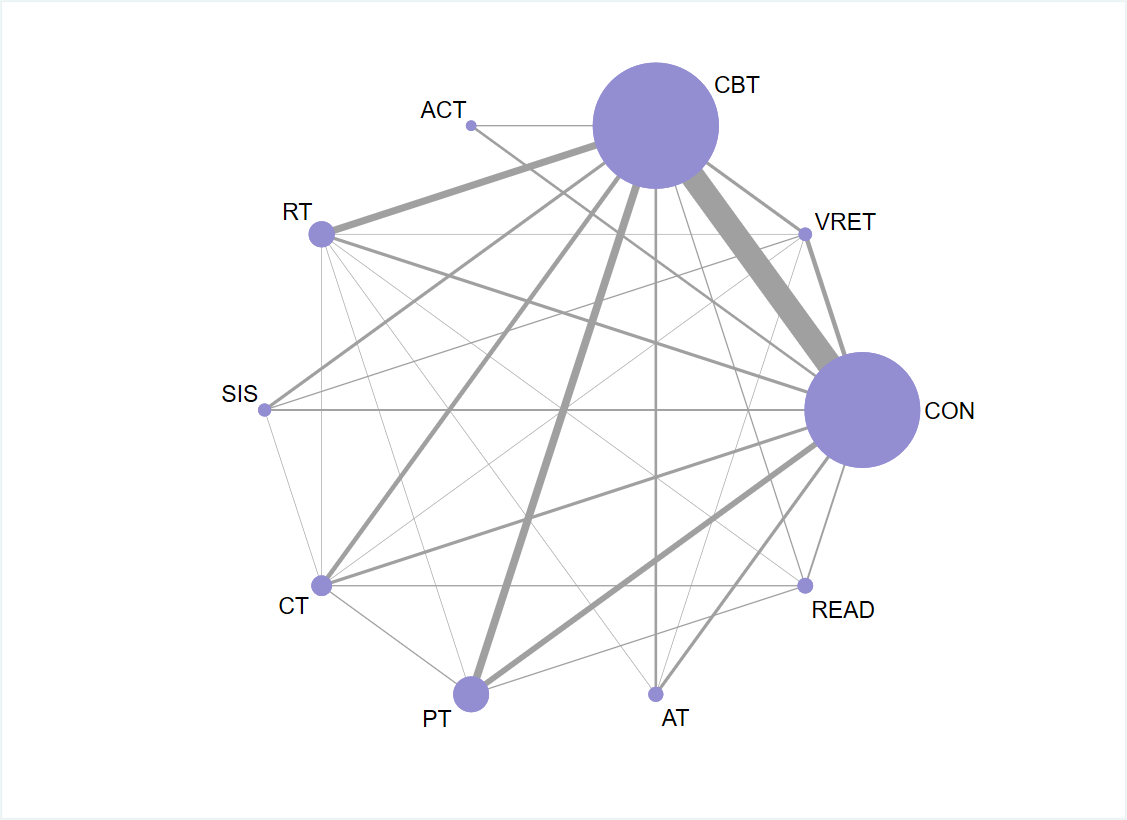


**Notes: CON:**Control group **VRET:**Virtual Reality Exposure Therapy **CBT:**Cognitive behavioral therapy **ACT:**Acceptance and Commitment Therapy **RT:**Relaxation therapy **SIS:**Social and Interpersonal Skills **CT**:Combination therapy **PT:**Psychotherapy **AT:**Attention training **READ:**Reading therapy

**Supplementary S12.2 –** **Network evidence graph for developing country**


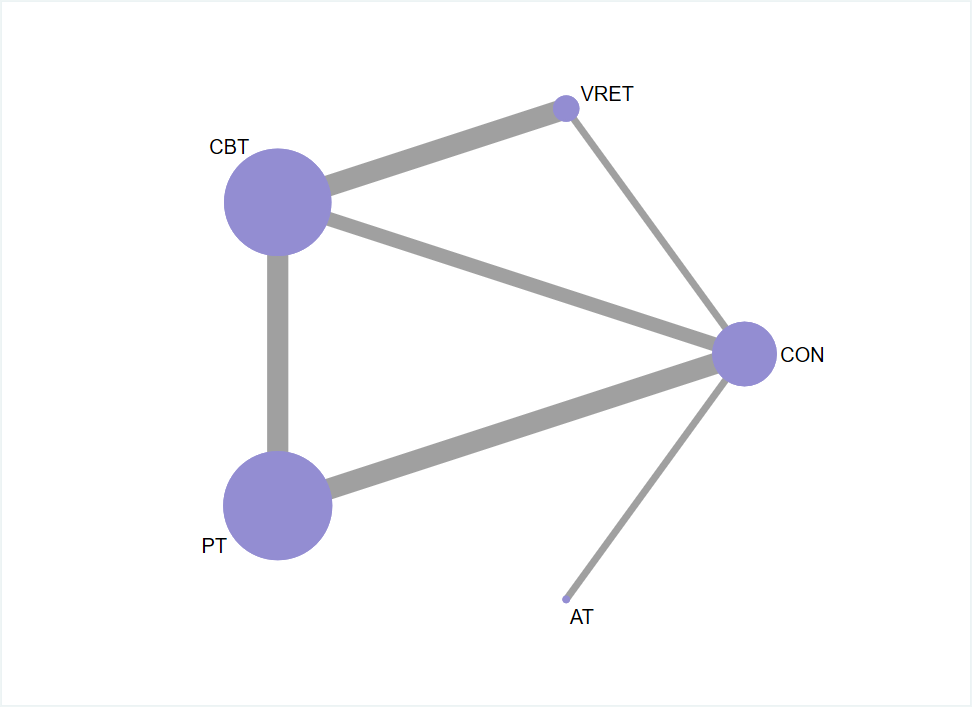


**Notes: CON:**Control group **VRET:**Virtual Reality Exposure Therapy **CBT:**Cognitive behavioral therapy **PT:**Psychotherapy **AT:**Attention training

**Supplementary S12.3 –** Forest plot and certainty evidence for developed country


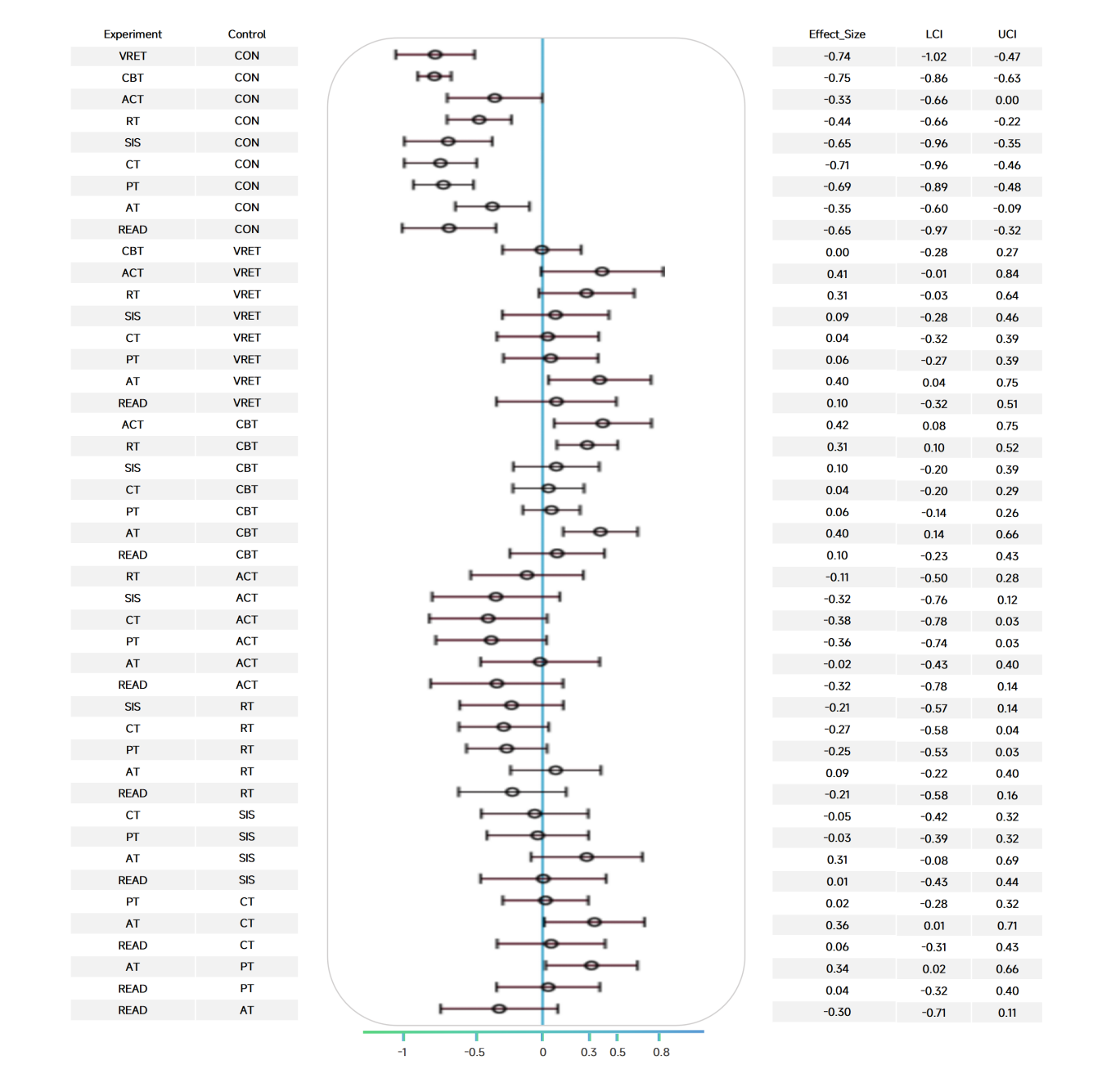


**Notes: CON:**Control group **VRET:**Virtual Reality Exposure Therapy **CBT:**Cognitive behavioral therapy **ACT:**Acceptance and Commitment Therapy **RT:**Relaxation therapy **SIS:**Social and Interpersonal Skills **CT**:Combination therapy **PT:**Psychotherapy **AT:**Attention training **READ:**Reading therapy

**Supplementary S12.4 –** Forest plot and certainty evidence for developing country


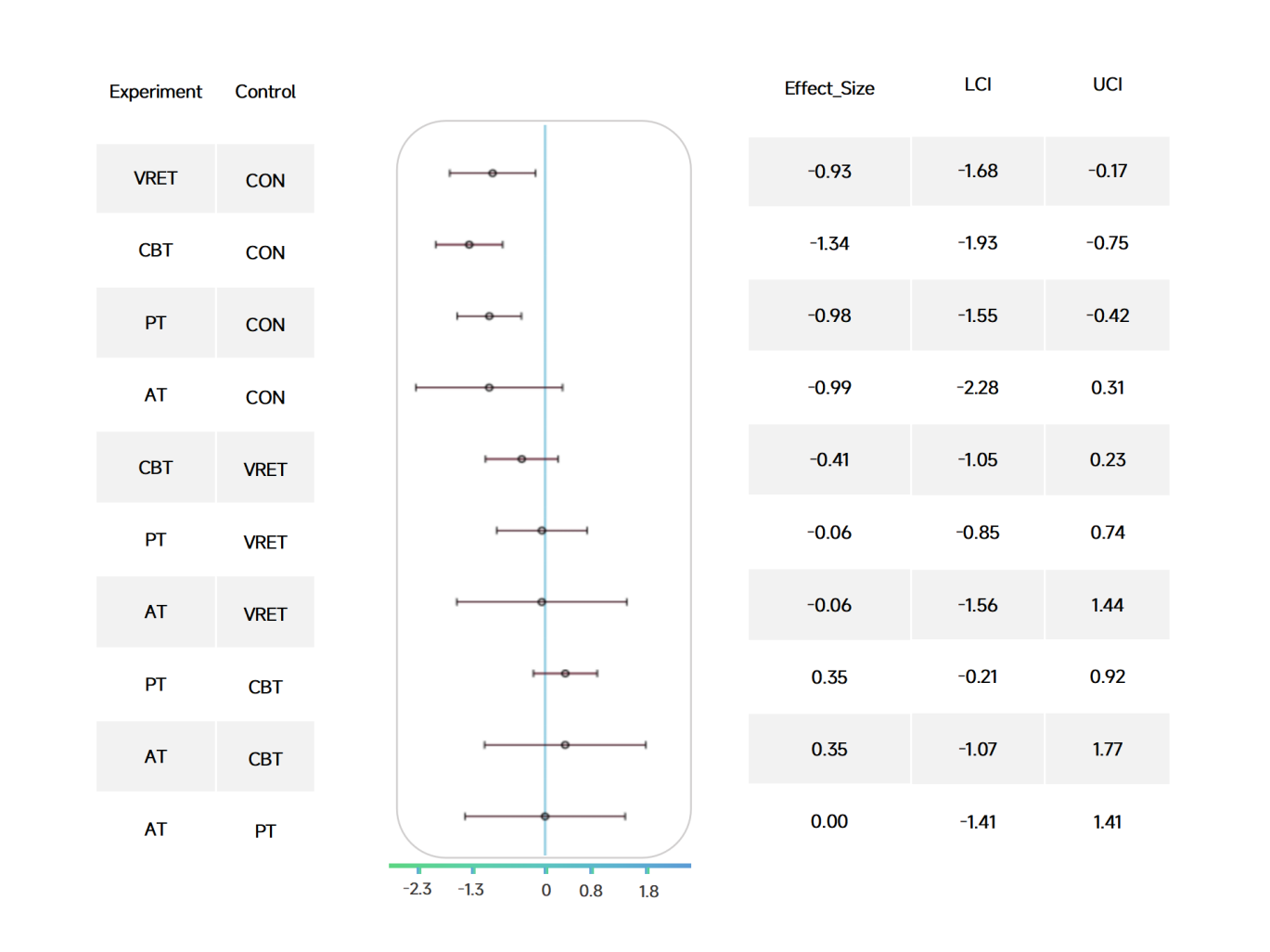


**Notes: CON:**Control group **VRET:**Virtual Reality Exposure Therapy **CBT:**Cognitive behavioral therapy **PT:**Psychotherapy **AT:**Attention training

**Supplementary S12.5 –** **Network evidence graph for Intervention duration of ≥ 8 weeks**


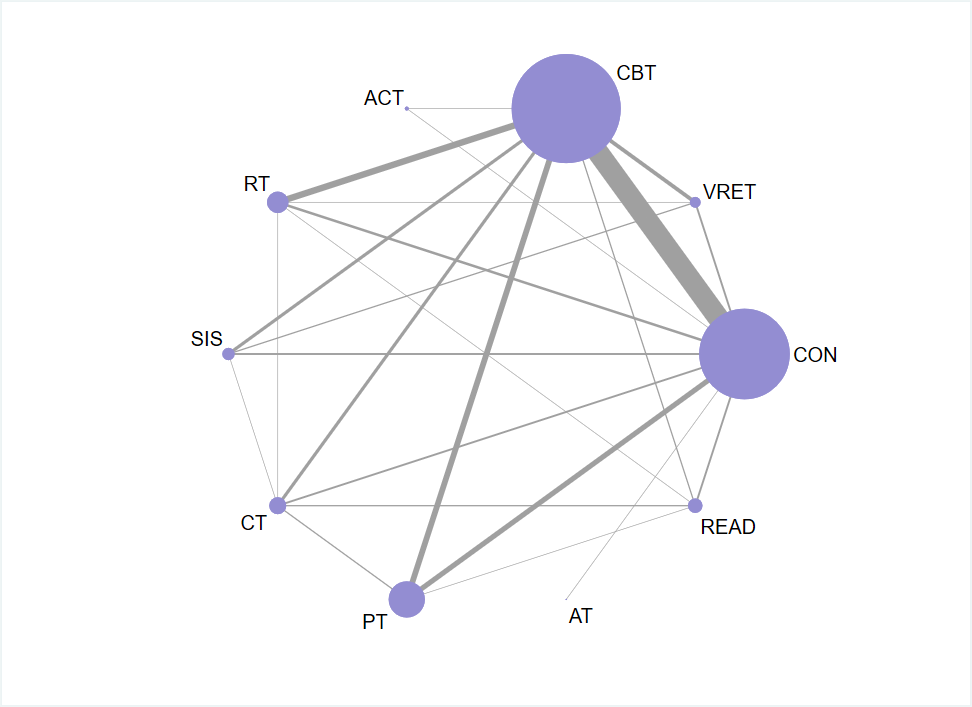


**Notes: CON:**Control group **VRET:**Virtual Reality Exposure Therapy **CBT:**Cognitive behavioral therapy **ACT:**Acceptance and Commitment Therapy **RT:**Relaxation therapy **SIS:**Social and Interpersonal Skills **CT**:Combination therapy **PT:**Psychotherapy **AT:**Attention training **READ:**Reading therapy

**Supplementary S12.6 –** **Network evidence graph for Intervention duration of ＜ 8 weeks**


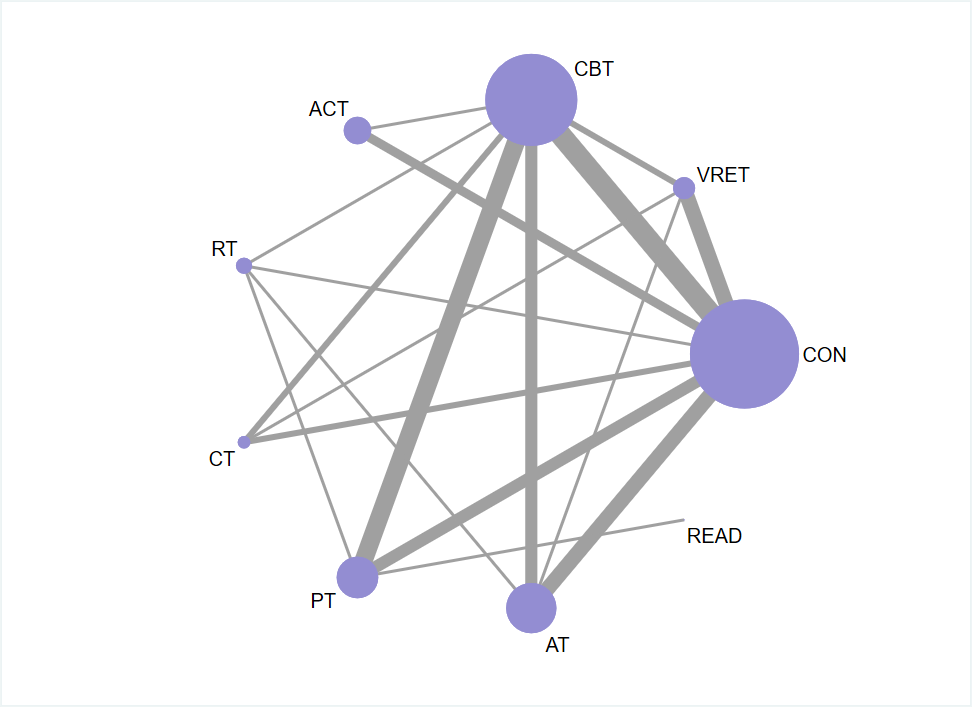


**Notes: CON:**Control group **VRET:**Virtual Reality Exposure Therapy **CBT:**Cognitive behavioral therapy **ACT:**Acceptance and Commitment Therapy **RT:**Relaxation therapy **CT**:Combination therapy **PT:**Psychotherapy **AT:**Attention training **READ:**Reading therapy

**Supplementary S12.7 –** Forest plot and certainty evidence for intervention duration of ≥ 8 weeks

**
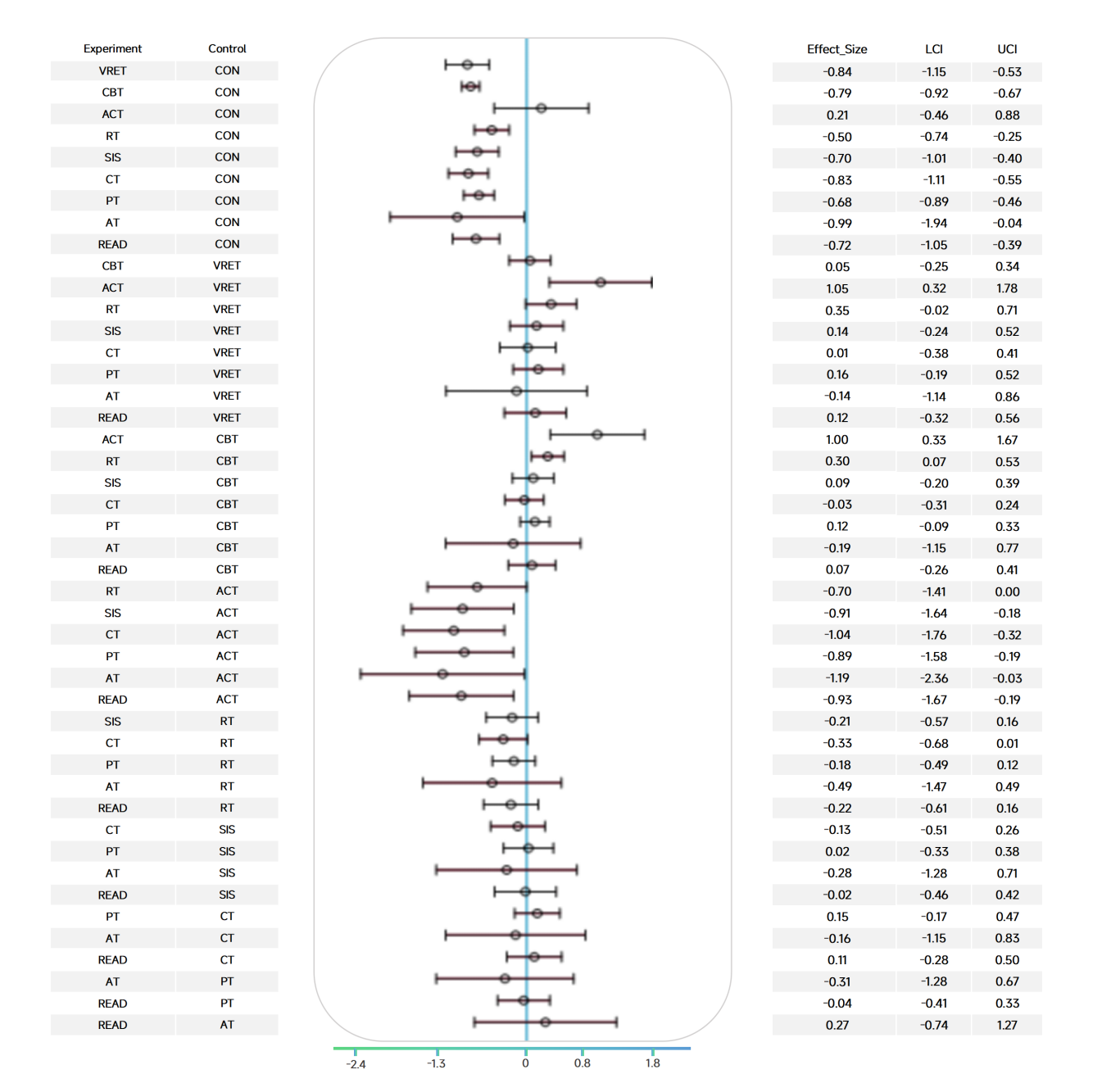
**

**Notes: CON:**Control group **VRET:**Virtual Reality Exposure Therapy **CBT:**Cognitive behavioral therapy **ACT:**Acceptance and Commitment Therapy **RT:**Relaxation therapy **SIS:**Social and Interpersonal Skills **CT**:Combination therapy **PT:**Psychotherapy **AT:**Attention training **READ:**Reading therapy

**Supplementary S12.8 –** Forest plot and certainty evidence for Intervention duration of ＜ 8 weeks

**
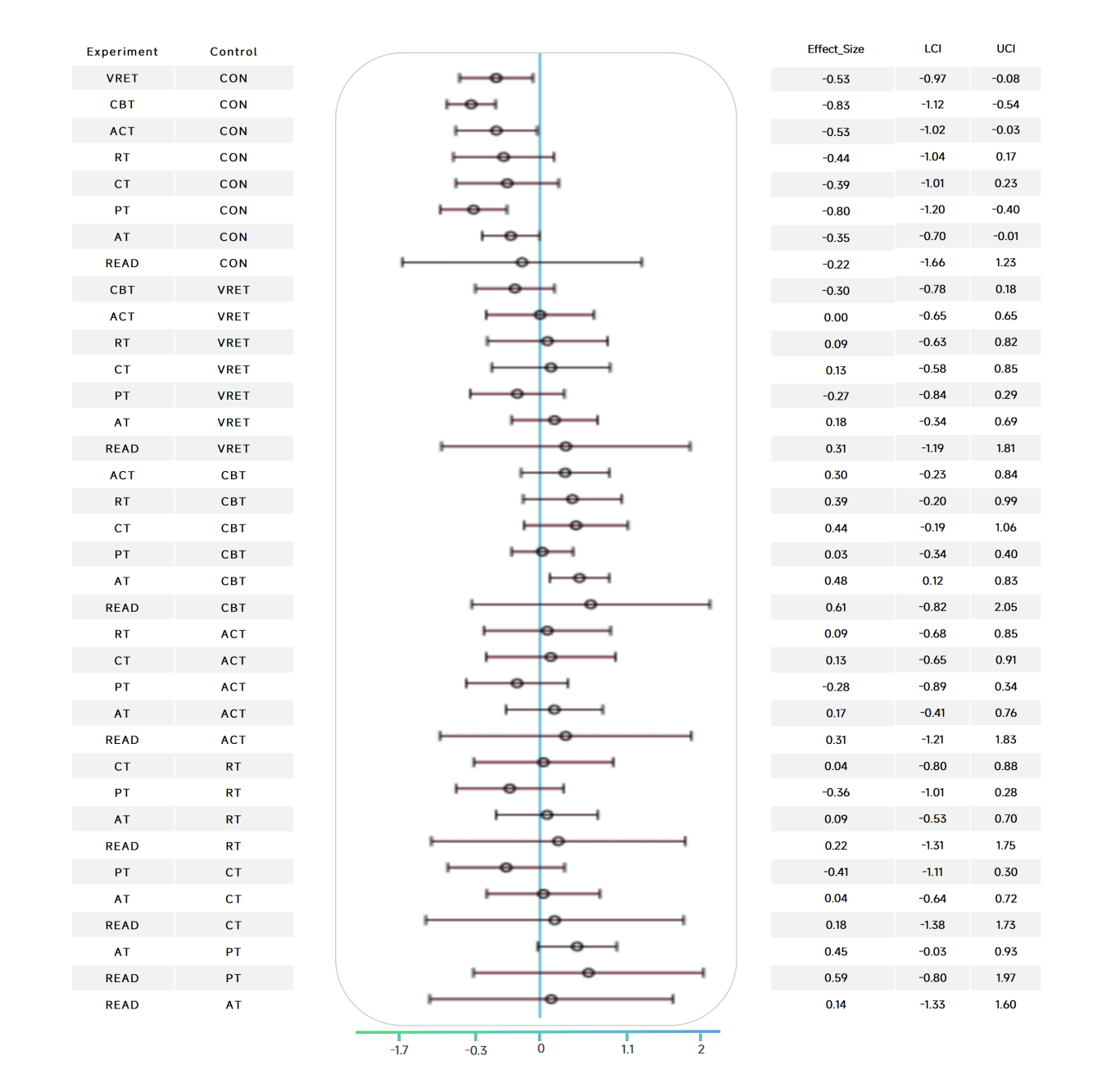
**

**Notes: CON:**Control group **VRET:**Virtual Reality Exposure Therapy **CBT:**Cognitive behavioral therapy **ACT:**Acceptance and Commitment Therapy **RT:**Relaxation therapy **CT**:Combination therapy **PT:**Psychotherapy **AT:**Attention training **READ:**Reading therapy

**Supplementary S12.9 –** Network evidence graph for Subclinical baseline severity

**
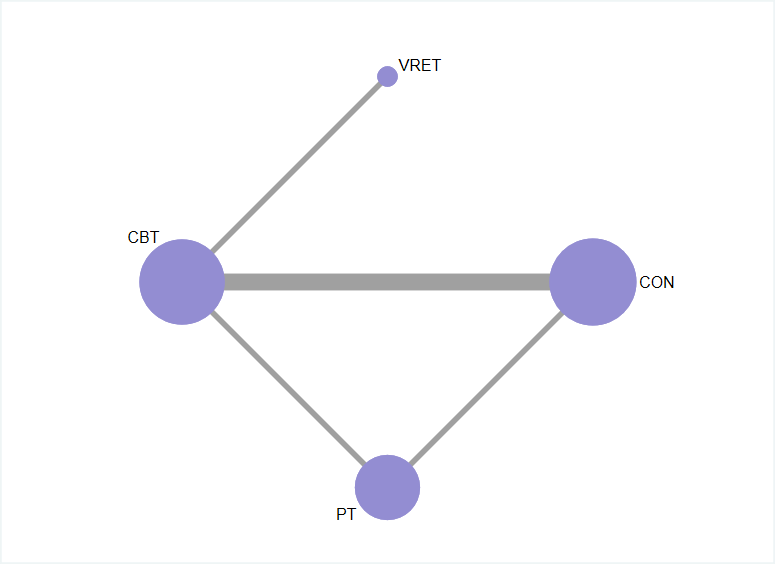
**

**Notes: CON:**Control group **VRET:**Virtual Reality Exposure Therapy **CBT:**Cognitive behavioral therapy **PT:**Psychotherapy

**Supplementary S12.10 –** Network evidence graph for Moderate to Severe baseline severity

**
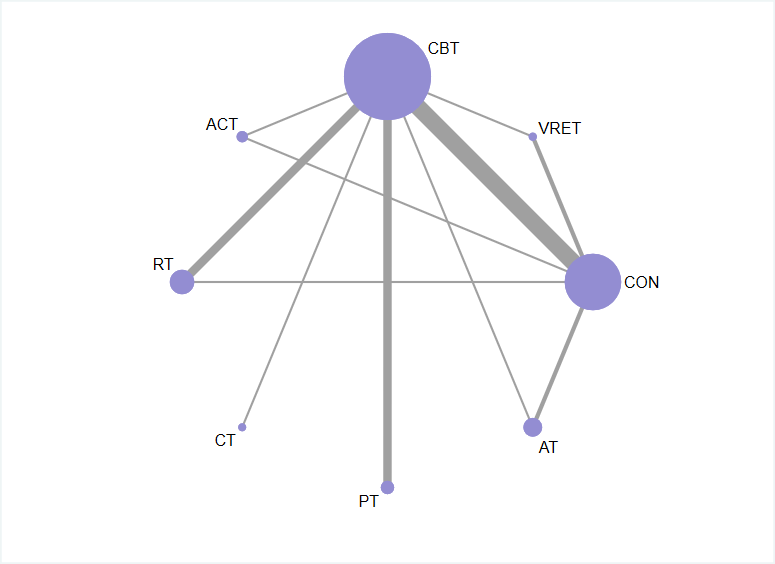
**

**Notes: CON:**Control group **VRET:**Virtual Reality Exposure Therapy **CBT:**Cognitive behavioral therapy **ACT:**Acceptance and Commitment Therapy **RT:**Relaxation therapy **CT**:Combination therapy **PT:**Psychotherapy **AT:**Attention training

**Supplementary S12.11 –** Network evidence graph for Severe baseline severity

**
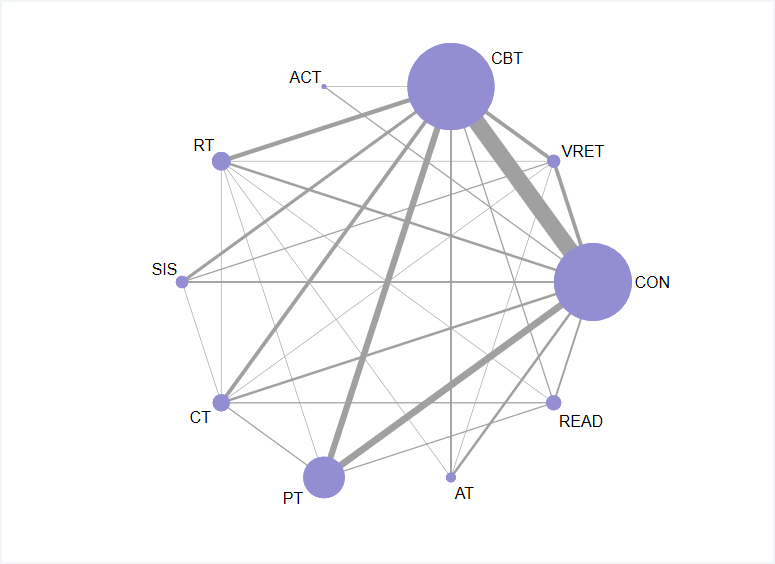
**

**Notes: CON:**Control group **VRET:**Virtual Reality Exposure Therapy **CBT:**Cognitive behavioral therapy **ACT:**Acceptance and Commitment Therapy **RT:**Relaxation therapy **SIS:**Social and Interpersonal Skills **CT**:Combination therapy **PT:**Psychotherapy **AT:**Attention training **READ:**Reading therapy

**Supplementary S12.12 –** Forest plot and certainty evidence for Subclinical baseline severity

**
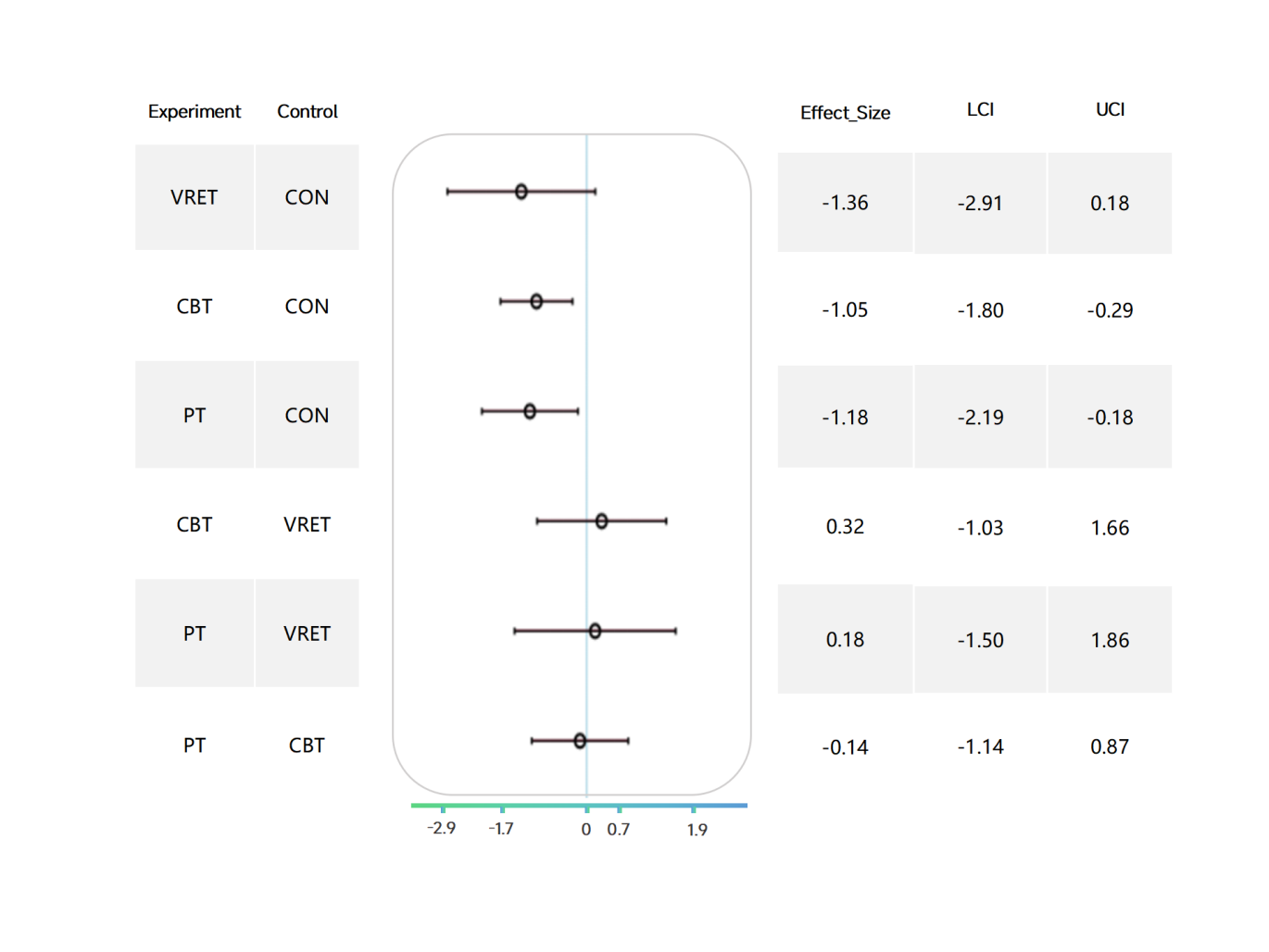
**

**Notes: CON:**Control group **VRET:**Virtual Reality Exposure Therapy **CBT:**Cognitive behavioral therapy **PT:**Psychotherapy

**Supplementary S12.13 –** Forest plot and certainty evidence for Moderate to Severe baseline severity

**
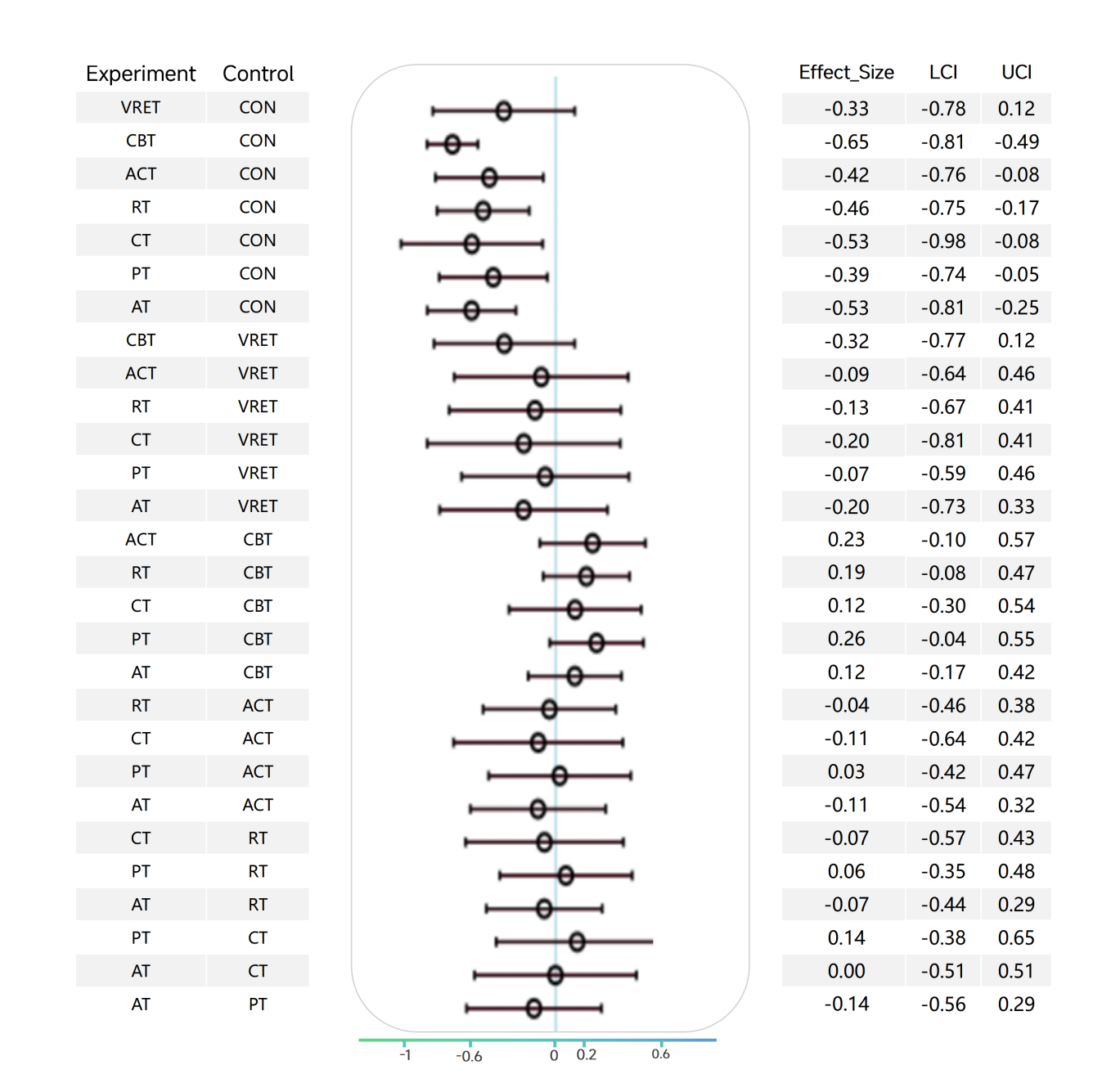
**

**Notes: CON:**Control group **VRET:**Virtual Reality Exposure Therapy **CBT:**Cognitive behavioral therapy **ACT:**Acceptance and Commitment Therapy **RT:**Relaxation therapy **CT**:Combination therapy **PT:**Psychotherapy **AT:**Attention training

**Supplementary S12.14 –** Forest plot and certainty evidence for Severe baseline severity

**
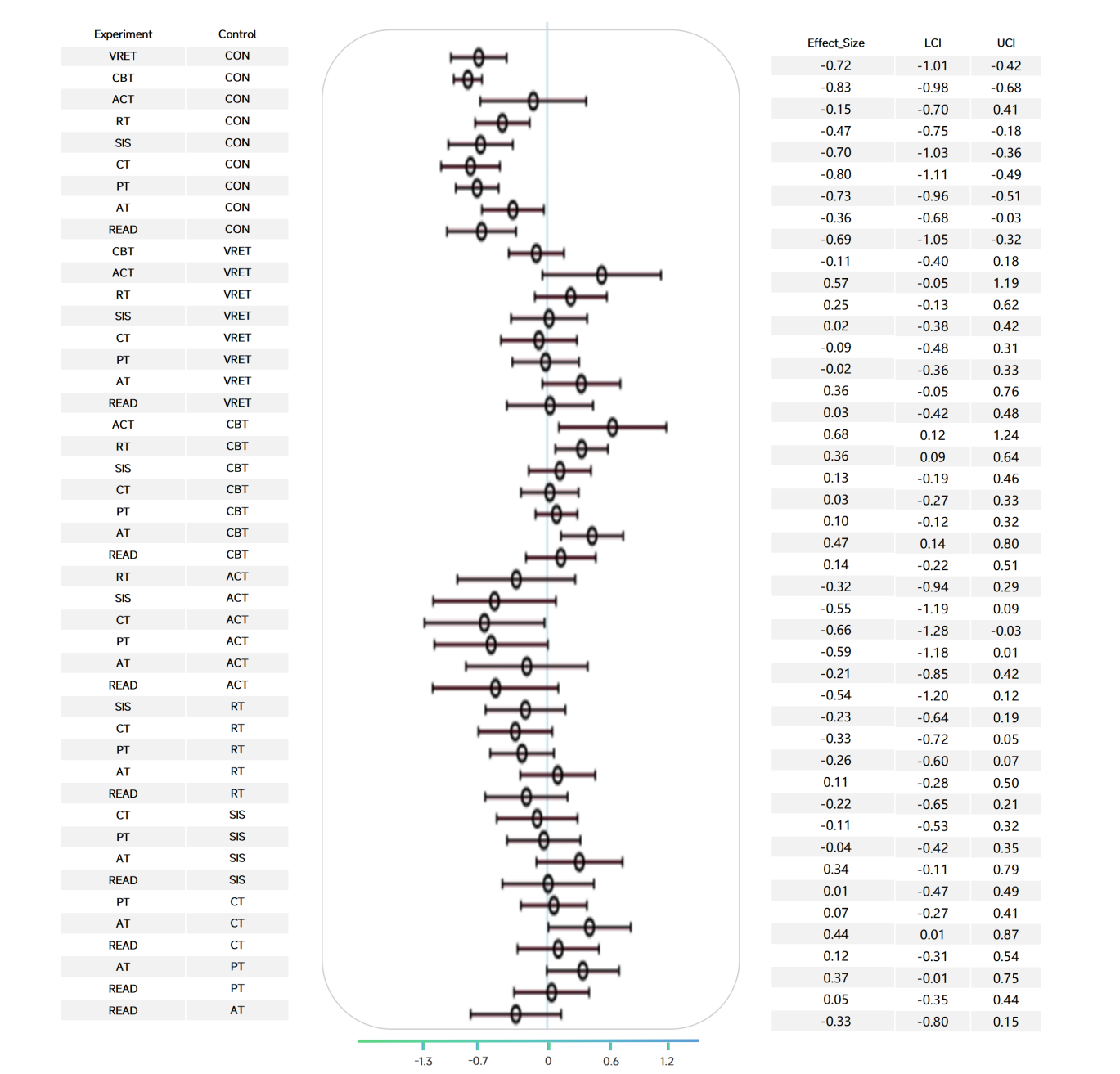
**

**Notes: CON:**Control group **VRET:**Virtual Reality Exposure Therapy **CBT:**Cognitive behavioral therapy **ACT:**Acceptance and Commitment Therapy **RT:**Relaxation therapy **SIS:**Social and Interpersonal Skills **CT**:Combination therapy **PT:**Psychotherapy **AT:**Attention training **READ:**Reading therapy

**Supplementary S12.15 –** Network evidence graph for Individual face-to-face format

**
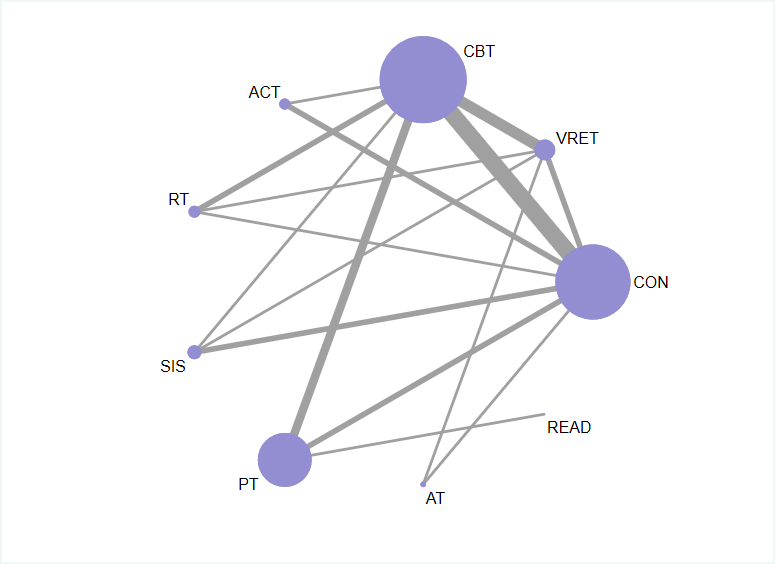
**

**Notes: CON:**Control group **VRET:**Virtual Reality Exposure Therapy **CBT:**Cognitive behavioral therapy **ACT:**Acceptance and Commitment Therapy **RT:**Relaxation therapy **SIS:**Social and Interpersonal Skills **PT:**Psychotherapy **AT:**Attention training **READ:**Reading therapy

**Supplementary S12.16 –** Network evidence graph for Group face-to-face format


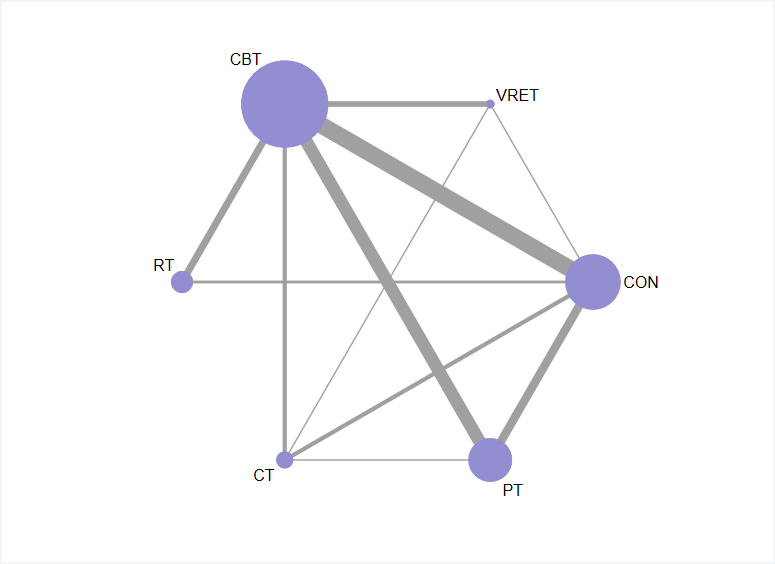


**Notes: CON:**Control group **VRET:**Virtual Reality Exposure Therapy **CBT:**Cognitive behavioral therapy **RT:**Relaxation therapy **CT**:Combination therapy **PT:**Psychotherapy

**Supplementary S12.17 –** Network evidence graph for Online format


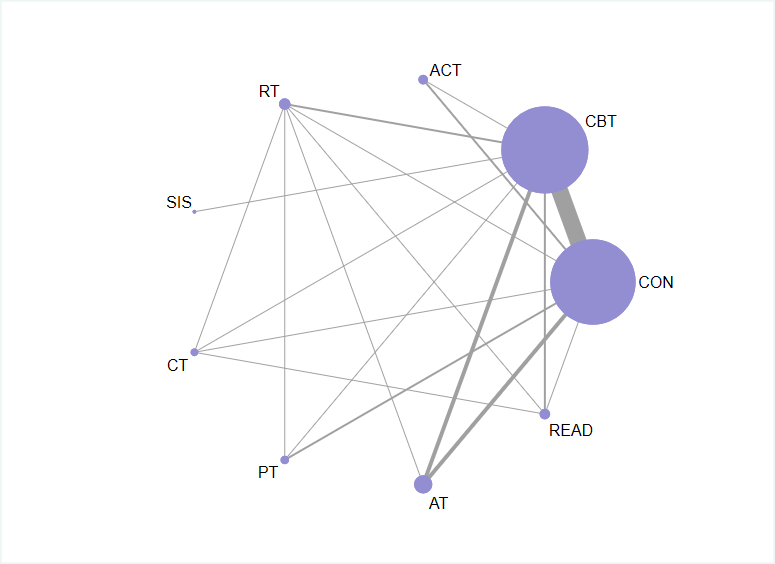


**Notes: CON:**Control group **CBT:**Cognitive behavioral therapy **ACT:**Acceptance and Commitment Therapy **RT:**Relaxation therapy **SIS:**Social and Interpersonal Skills **CT**:Combination therapy **PT:**Psychotherapy **AT:**Attention training **READ:**Reading therapy

**Supplementary S12.18 –** Network evidence graph for Mixed format

**
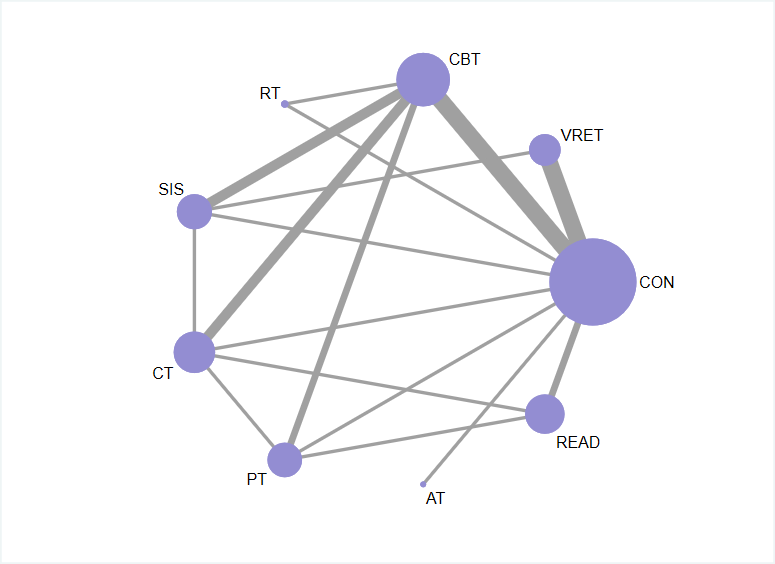
**

**Notes: CON:**Control group **VRET:**Virtual Reality Exposure Therapy **CBT:**Cognitive behavioral therapy **RT:**Relaxation therapy **SIS:**Social and Interpersonal Skills **CT**:Combination therapy **PT:**Psychotherapy **AT:**Attention training **READ:**Reading therapy

**Supplementary S12.19 –** Forest plot and certainty evidence for Individual face-to-face format

**
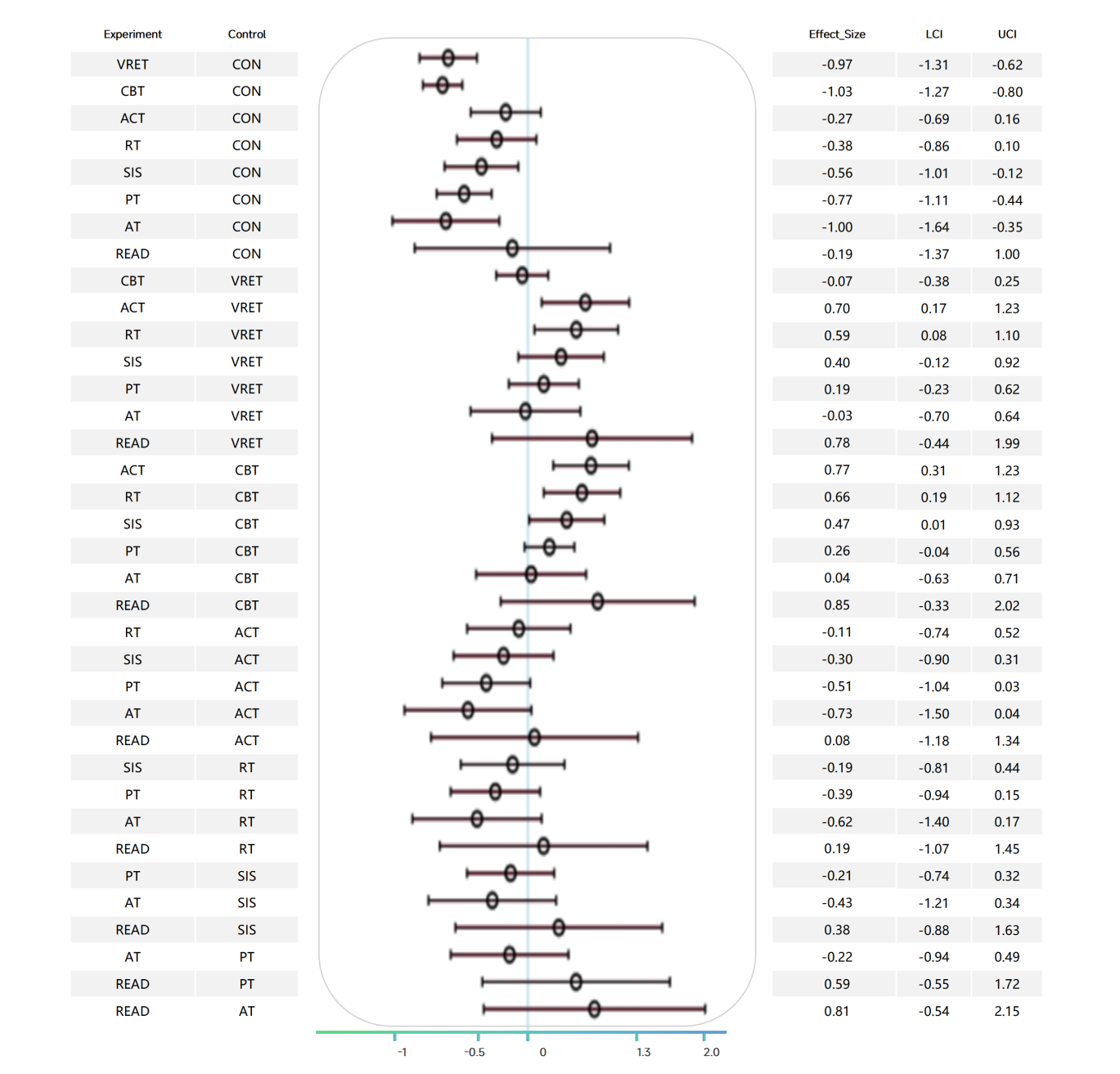
**

**Notes: CON:**Control group **VRET:**Virtual Reality Exposure Therapy **CBT:**Cognitive behavioral therapy **ACT:**Acceptance and Commitment Therapy **RT:**Relaxation therapy **SIS:**Social and Interpersonal Skills **PT:**Psychotherapy **AT:**Attention training **READ:**Reading therapy

**Supplementary S12.20 –** Forest plot and certainty evidence for Group face-to-face format


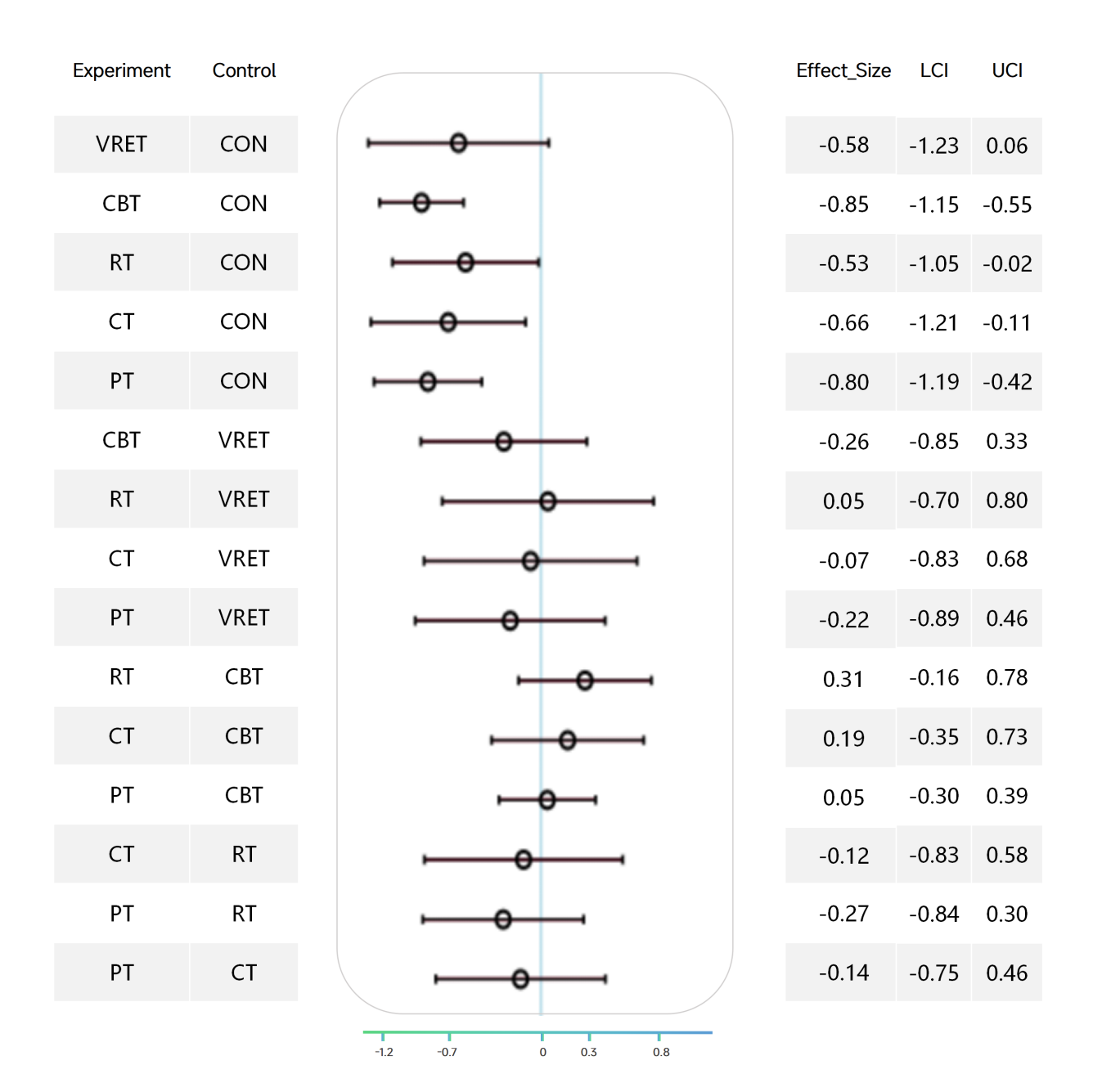


**Notes: CON:**Control group **VRET:**Virtual Reality Exposure Therapy **CBT:**Cognitive behavioral therapy **RT:**Relaxation therapy **CT**:Combination therapy **PT:**Psychotherapy

**Supplementary S12.21 –** Forest plot and certainty evidence for Online format


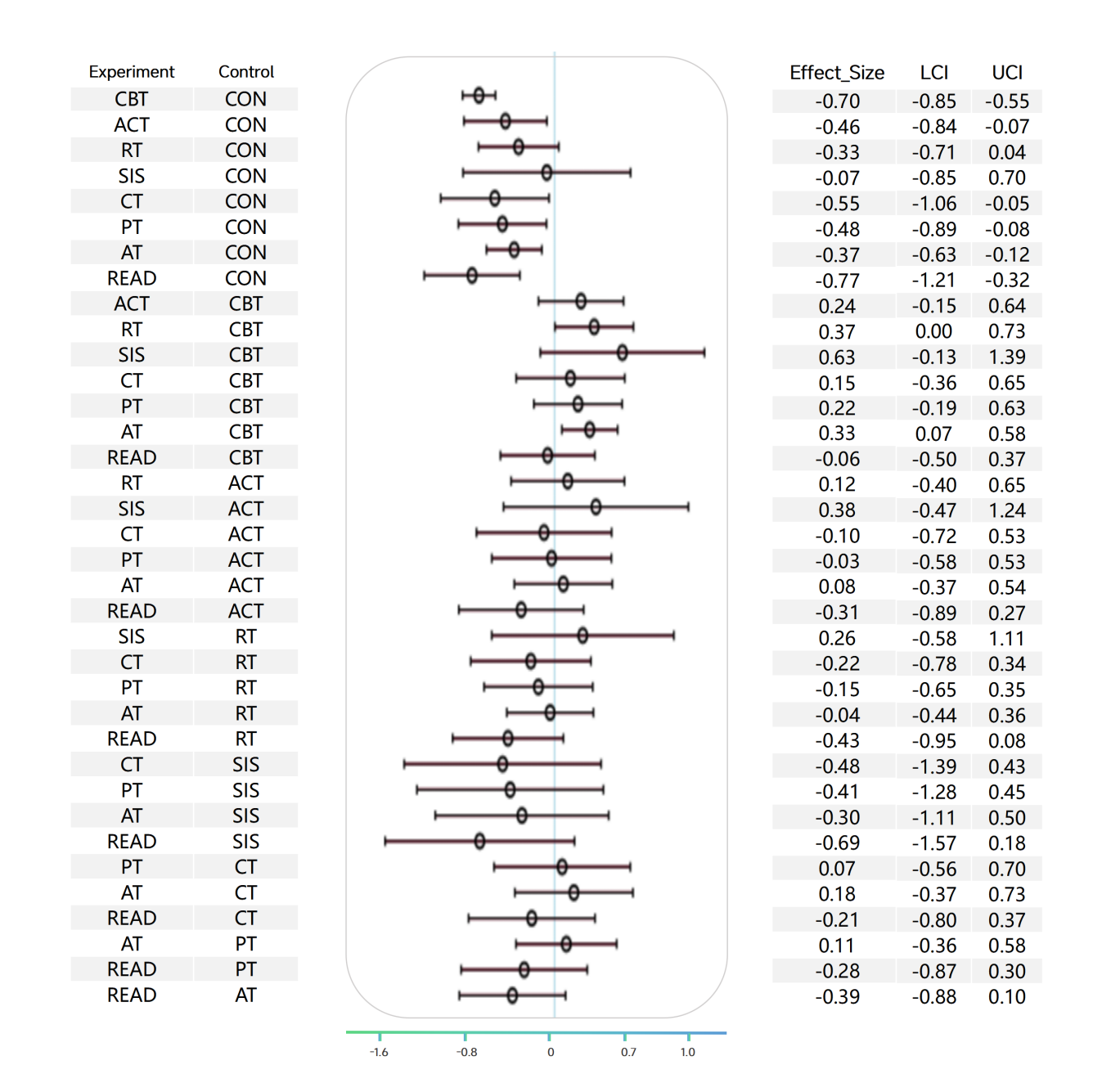


**Notes: CON:**Control group **CBT:**Cognitive behavioral therapy **ACT:**Acceptance and Commitment Therapy **RT:**Relaxation therapy **SIS:**Social and Interpersonal Skills **CT**:Combination therapy **PT:**Psychotherapy **AT:**Attention training **READ:**Reading therapy

**Supplementary S12.22 –** Forest plot and certainty evidence for Mixed format


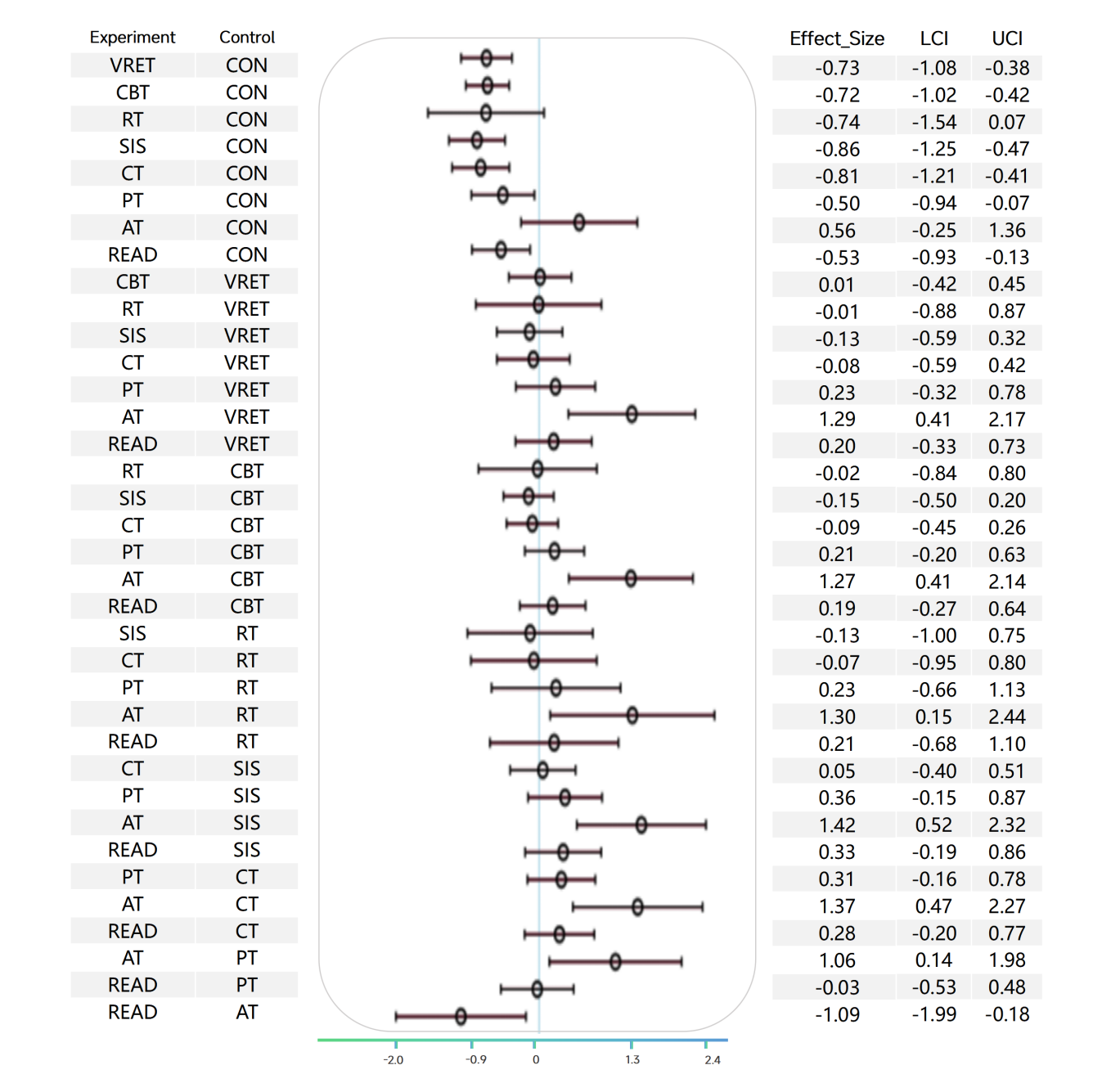


**Notes: CON:**Control group **VRET:**Virtual Reality Exposure Therapy **CBT:**Cognitive behavioral therapy **RT:**Relaxation therapy **SIS:**Social and Interpersonal Skills **CT**:Combination therapy **PT:**Psychotherapy **AT:**Attention training **READ:**Reading therapy

**Supplementary S12.23 –** Comparison-adjusted funnel plot for **developed country**


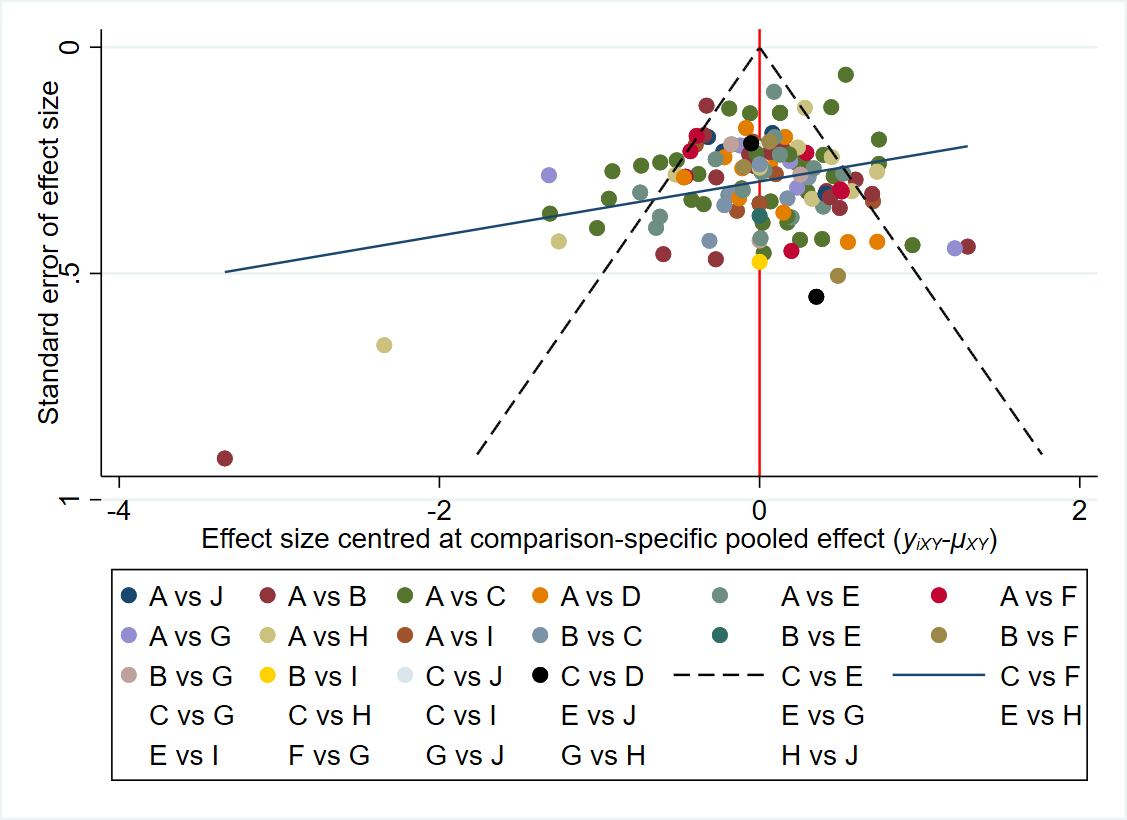


**Notes: A:**CON **B:**VRET **C:**CBT **D:**ACT **E:**RT **F:**SIS **G:**CT **H:**PT **I:**AT **J:**READ

**Notes: CON:**Control group **VRET:**Virtual Reality Exposure Therapy **CBT:**Cognitive behavioral therapy **ACT:**Acceptance and Commitment Therapy **RT:**Relaxation therapy **SIS:**Social and Interpersonal Skills **CT**:Combination therapy **PT:**Psychotherapy **AT:**Attention training **READ:**Reading therapy

**Supplementary S12.24 –** Comparison-adjusted funnel plot for **developing country**


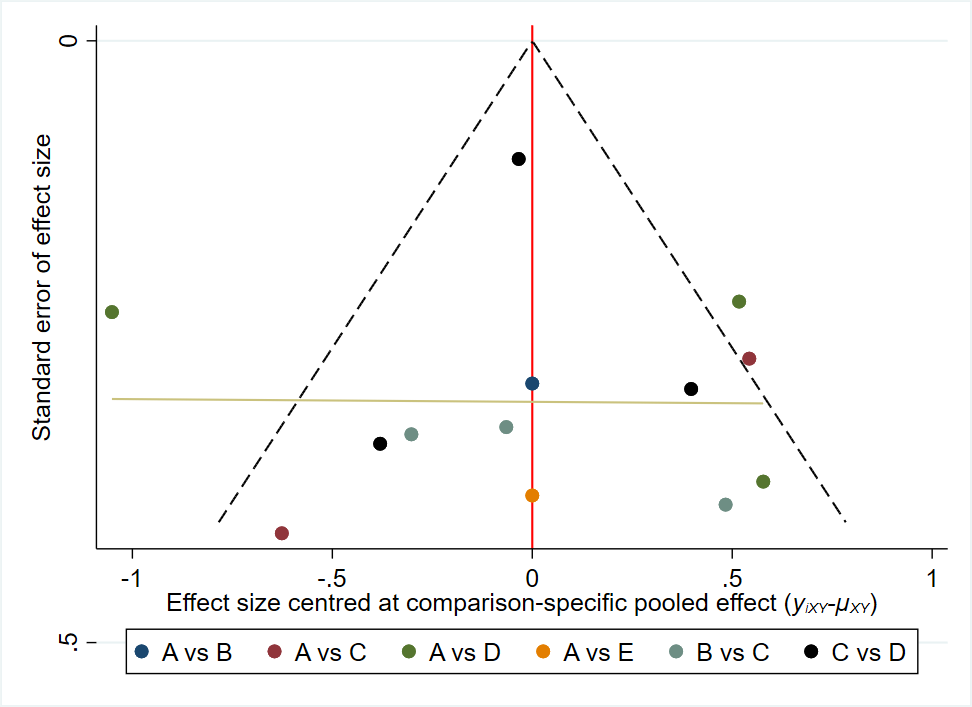


**Notes: A:**CON **B:**VRET **C:**CBT **D:**PT **E:**AT

**Notes: CON:**Control group **VRET:**Virtual Reality Exposure Therapy **CBT:**Cognitive behavioral therapy **PT:**Psychotherapy **AT:**Attention training

**Supplementary S12.25** – Comparison-adjusted funnel plot for Intervention duration of ≥ 8 weeks


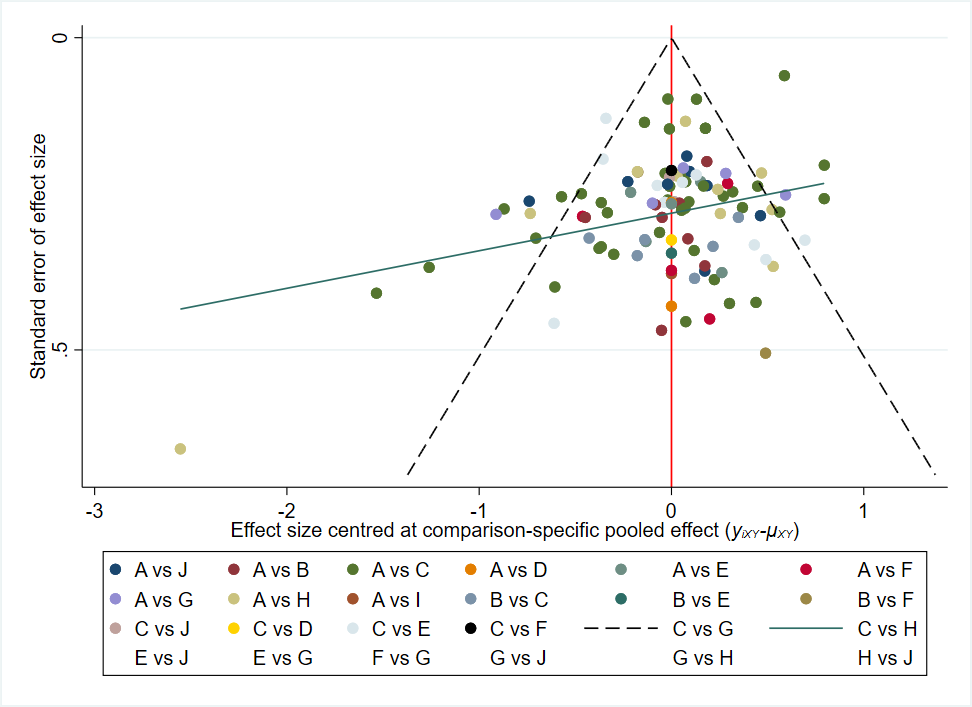


**Notes: A:**CON **B:**VRET **C:**CBT **D:**ACT **E:**RT **F:**SIS **G:**CT **H:**PT **I:**AT **J:**READ

**Notes: CON:**Control group **VRET:**Virtual Reality Exposure Therapy **CBT:**Cognitive behavioral therapy **ACT:**Acceptance and Commitment Therapy **RT:**Relaxation therapy **SIS:**Social and Interpersonal Skills **CT**:Combination therapy **PT:**Psychotherapy **AT:**Attention training **READ:**Reading therapy

**Supplementary S12.26 –** Comparison-adjusted funnel plot for **Intervention duration of ＜ 8 weeks**


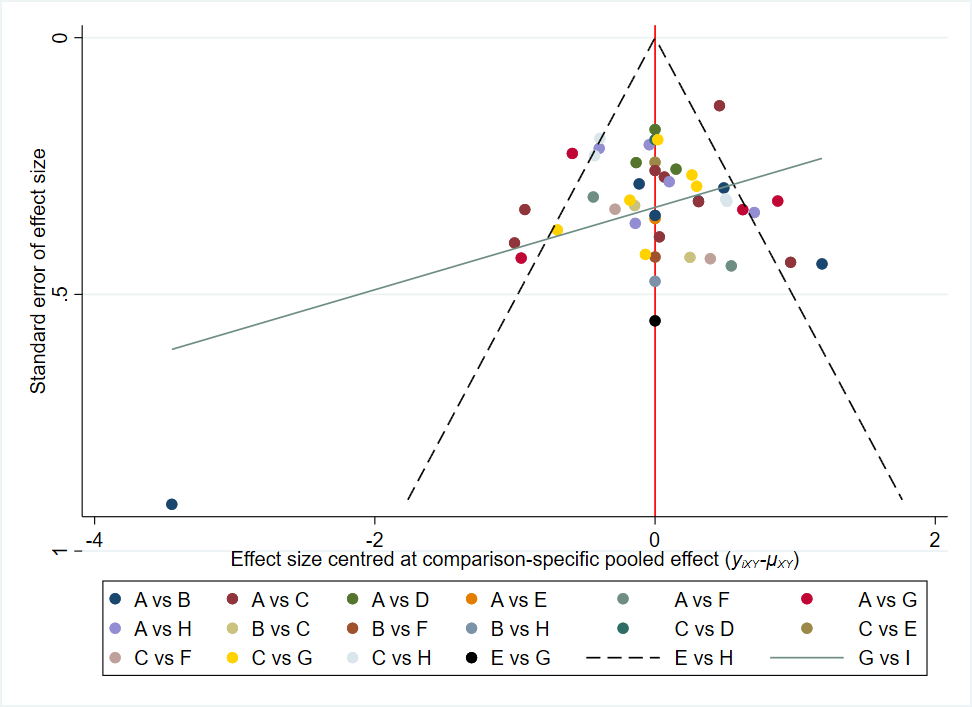


**Notes: A:**CON **B:**VRET **C:**CBT **D:**ACT **E:**RT **F:**CT **G:**PT **H:**AT **I:**READ

**Notes: CON:**Control group **VRET:**Virtual Reality Exposure Therapy **CBT:**Cognitive behavioral therapy **ACT:**Acceptance and Commitment Therapy **RT:**Relaxation therapy **CT**:Combination therapy **PT:**Psychotherapy **AT:**Attention training **READ:**Reading therapy

**Supplementary S12.27 –** Comparison-adjusted funnel plot for Subclinical baseline severity


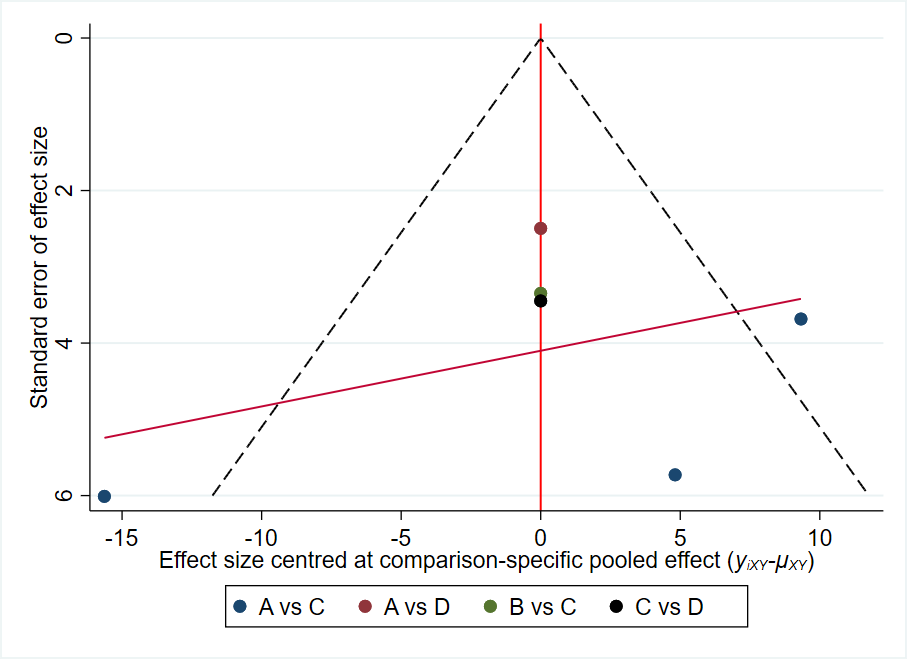


**Notes: A:**CON **B:**VRET **C:**CBT **D:**PT

**Notes: CON:**Control group **VRET:**Virtual Reality Exposure Therapy **CBT:**Cognitive behavioral therapy **PT:**Psychotherapy

**Supplementary S12.28 –** Comparison-adjusted funnel plot for Moderate to Severe baseline severity


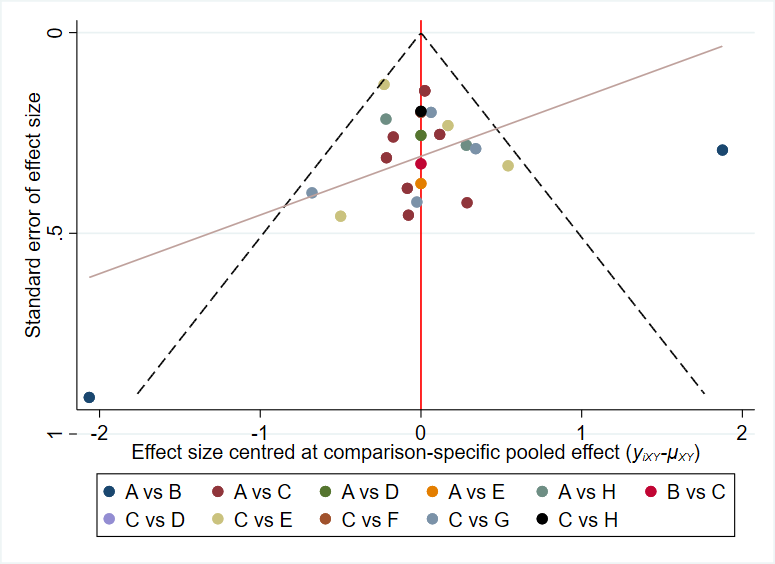


**Notes: A:**CON **B:**VRET **C:**CBT **D:**ACT **E:**RT **F:**CT **G:**PT **H:**AT

**Notes: CON:**Control group **VRET:**Virtual Reality Exposure Therapy **CBT:**Cognitive behavioral therapy **ACT:**Acceptance and Commitment Therapy **RT:**Relaxation therapy **CT**:Combination therapy **PT:**Psychotherapy **AT:**Attention training

**Supplementary S12.29 –** Comparison-adjusted funnel plot for Severe baseline severity


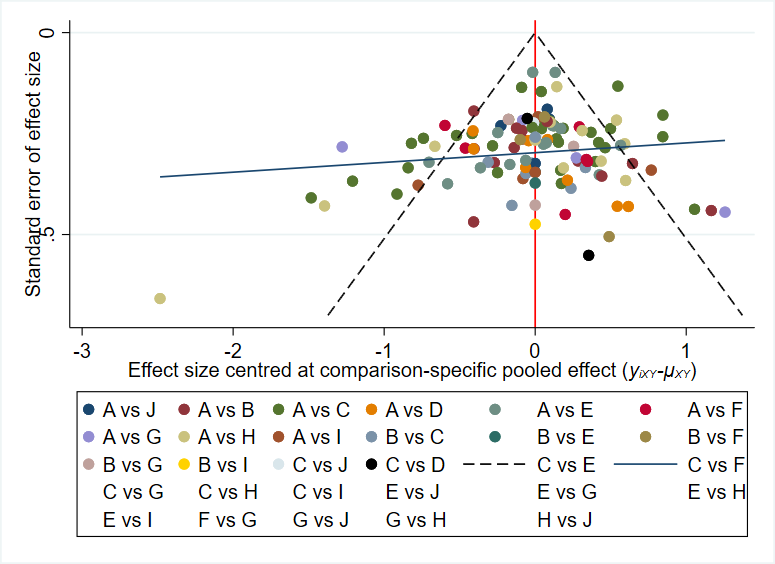


**Notes: A:**CON **B:**VRET **C:**CBT **D:**ACT **E:**RT **F:**SIS **G:**CT **H:**PT **I:**AT **J:**READ

**Notes: CON:**Control group **VRET:**Virtual Reality Exposure Therapy **CBT:**Cognitive behavioral therapy **ACT:**Acceptance and Commitment Therapy **RT:**Relaxation therapy **SIS:**Social and Interpersonal Skills **CT**:Combination therapy **PT:**Psychotherapy **AT:**Attention training **READ:**Reading therapy

**Supplementary S12.30 –** Comparison-adjusted funnel plot for Individual face-to-face format


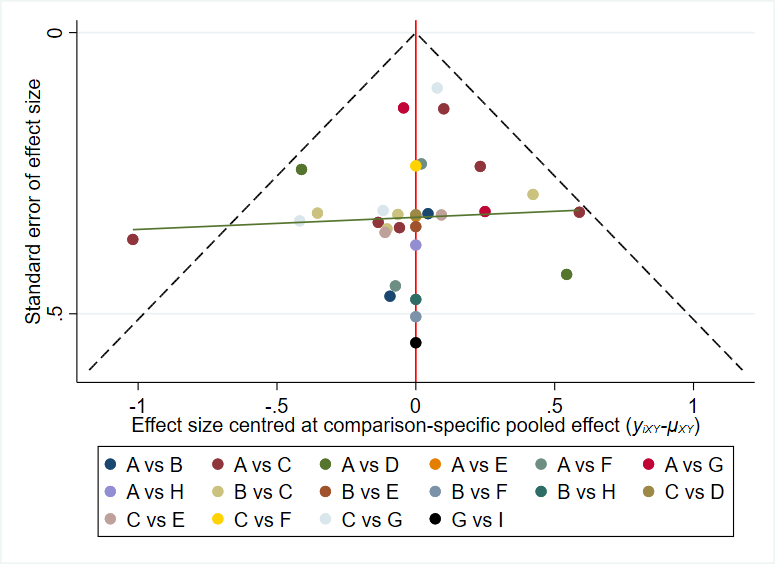


**Notes: A:**CON **B:**VRET **C:**CBT **D:**ACT **E:**RT **F:**SIS **G:**PT **H:**AT **I**:READ

**Notes: CON:**Control group **VRET:**Virtual Reality Exposure Therapy **CBT:**Cognitive behavioral therapy **ACT:**Acceptance and Commitment Therapy **RT:**Relaxation therapy **SIS:**Social and Interpersonal Skills **PT:**Psychotherapy **AT:**Attention training **READ:**Reading therapy

**Supplementary S12.31 –** Comparison-adjusted funnel plot for Group face-to-face format


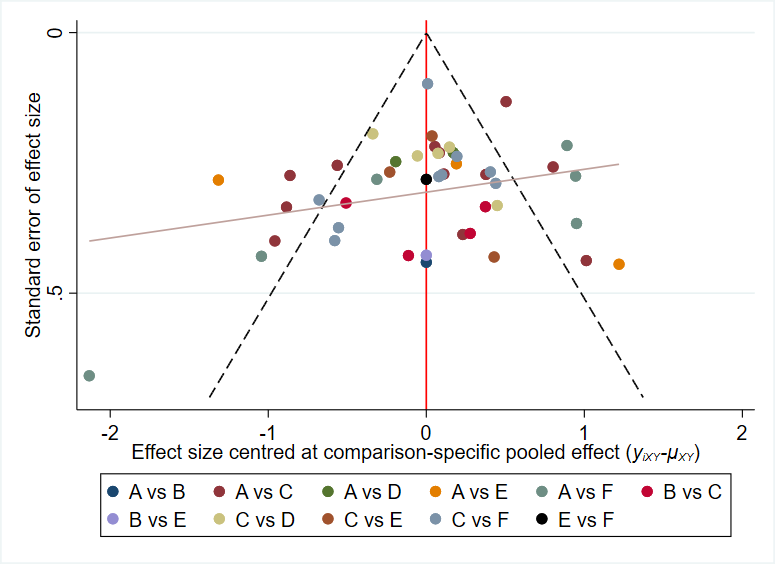


**Notes: A:**CON **B:**VRET **C:**CBT **D:**RT **E:**CT **F:**PT

**Notes: CON:**Control group **VRET:**Virtual Reality Exposure Therapy **CBT:**Cognitive behavioral therapy **RT:**Relaxation therapy **CT**:Combination therapy **PT:**Psychotherapy

**Supplementary S12.32 –** Comparison-adjusted funnel plot for Online format


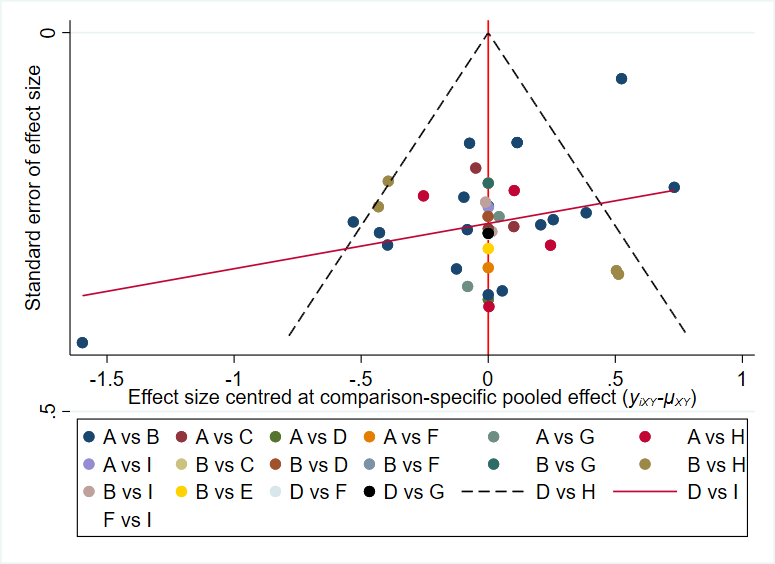


**Notes: A:**CON **B:**CBT **C:**ACT **D:**RT **E:**SIS **F:**CT **G:**PT **H:**AT **I:**READ

**Notes: CON:**Control group **CBT:**Cognitive behavioral therapy **ACT:**Acceptance and Commitment Therapy **RT:**Relaxation therapy **SIS:**Social and Interpersonal Skills **CT**:Combination therapy **PT:**Psychotherapy **AT:**Attention training **READ:**Reading therapy

**Supplementary S12.33 –** Comparison-adjusted funnel plot for Mixed format


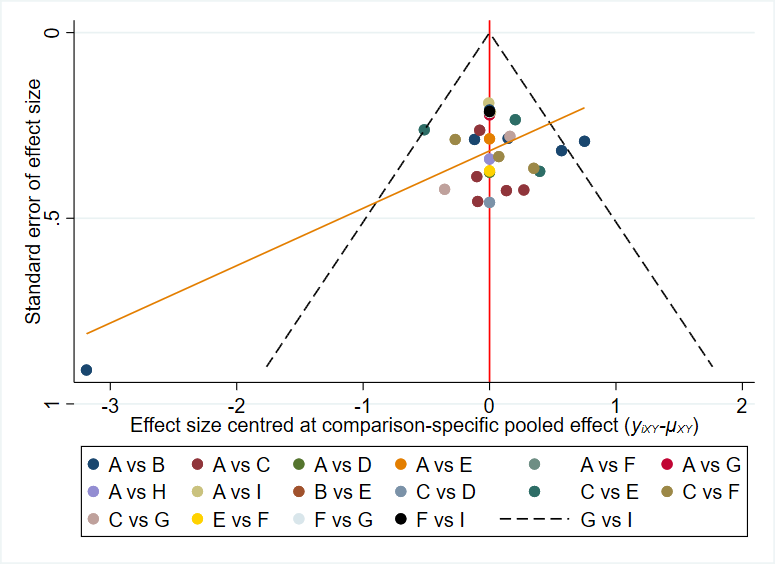


**Notes: A:**CON **B:**VRET **C:**CBT **D:**RT **E:**SIS **F:**CT **G:**PT **H:**AT **I:**READ

**Notes: CON:**Control group **VRET:**Virtual Reality Exposure Therapy **CBT:**Cognitive behavioral therapy **RT:**Relaxation therapy **SIS:**Social and Interpersonal Skills **CT**:Combination therapy **PT:**Psychotherapy **AT:**Attention training **READ:**Reading therapy

**Supplementary S12.34 –** League table for **developed country**

| CON | -0.74 (-1.02,-0.47) | -0.75 (-0.86,-0.63) | -0.33 (-0.66,-0.00) | -0.44 (-0.66,-0.22) | -0.65 (-0.96,-0.35) | -0.71 (-0.96,-0.46) | -0.69 (-0.89,-0.48) | -0.35 (-0.60,-0.09) | -0.65 (-0.97,-0.32) |
| --- | --- | --- | --- | --- | --- | --- | --- | --- | --- |
| 0.74 (0.47,1.02) | VRET | -0.00 (-0.28,0.27) | 0.41 (-0.01,0.84) | 0.31 (-0.03,0.64) | 0.09 (-0.28,0.46) | 0.04 (-0.32,0.39) | 0.06 (-0.27,0.39) | 0.40 (0.04,0.75) | 0.10 (-0.32,0.51) |
| 0.75 (0.63,0.86) | 0.00 (-0.27,0.28) | CBT | 0.42 (0.08,0.75) | 0.31 (0.10,0.52) | 0.10 (-0.20,0.39) | 0.04 (-0.20,0.29) | 0.06 (-0.14,0.26) | 0.40 (0.14,0.66) | 0.10 (-0.23,0.43) |
| 0.33 (0.00,0.66) | -0.41 (-0.84,0.01) | -0.42 (-0.75,-0.08) | ACT | -0.11 (-0.50,0.28) | -0.32 (-0.76,0.12) | -0.38 (-0.78,0.03) | -0.36 (-0.74,0.03) | -0.02 (-0.43,0.40) | -0.32 (-0.78,0.14) |
| 0.44 (0.22,0.66) | -0.31 (-0.64,0.03) | -0.31 (-0.52,-0.10) | 0.11 (-0.28,0.50) | RT | -0.21 (-0.57,0.14) | -0.27 (-0.58,0.04) | -0.25 (-0.53,0.03) | 0.09 (-0.22,0.40) | -0.21 (-0.58,0.16) |
| 0.65 (0.35,0.96) | -0.09 (-0.46,0.28) | -0.10 (-0.39,0.20) | 0.32 (-0.12,0.76) | 0.21 (-0.14,0.57) | SIS | -0.05 (-0.42,0.32) | -0.03 (-0.39,0.32) | 0.31 (-0.08,0.69) | 0.01 (-0.43,0.44) |
| 0.71 (0.46,0.96) | -0.04 (-0.39,0.32) | -0.04 (-0.29,0.20) | 0.38 (-0.03,0.78) | 0.27 (-0.04,0.58) | 0.05 (-0.32,0.42) | CT | 0.02 (-0.28,0.32) | 0.36 (0.01,0.71) | 0.06 (-0.31,0.43) |
| 0.69 (0.48,0.89) | -0.06 (-0.39,0.27) | -0.06 (-0.26,0.14) | 0.36 (-0.03,0.74) | 0.25 (-0.03,0.53) | 0.03 (-0.32,0.39) | -0.02 (-0.32,0.28) | PT | 0.34 (0.02,0.66) | 0.04 (-0.32,0.40) |
| 0.35 (0.09,0.60) | -0.40 (-0.75,-0.04) | -0.40 (-0.66,-0.14) | 0.02 (-0.40,0.43) | -0.09 (-0.40,0.22) | -0.31 (-0.69,0.08) | -0.36 (-0.71,-0.01) | -0.34 (-0.66,-0.02) | AT | -0.30 (-0.71,0.11) |
| 0.65 (0.32,0.97) | -0.10 (-0.51,0.32) | -0.10 (-0.43,0.23) | 0.32 (-0.14,0.78) | 0.21 (-0.16,0.58) | -0.01 (-0.44,0.43) | -0.06 (-0.43,0.31) | -0.04 (-0.40,0.32) | 0.30 (-0.11,0.71) | READ |

**Notes: CON:**Control group **VRET:**Virtual Reality Exposure Therapy **CBT:**Cognitive behavioral therapy **ACT:**Acceptance and Commitment Therapy **RT:**Relaxation therapy **SIS:**Social and Interpersonal Skills **CT**:Combination therapy **PT:**Psychotherapy **AT:**Attention training **READ:**Reading therapy

**Supplementary S12.35 –** League table for **developing country**

| CON | -0.93 (-1.68,-0.17) | -1.34 (-1.93,-0.75) | -0.98 (-1.55,-0.42) | -0.99 (-2.28,0.31) |
| --- | --- | --- | --- | --- |
| 0.93 (0.17,1.68) | VRET | -0.41 (-1.05,0.23) | -0.06 (-0.85,0.74) | -0.06 (-1.56,1.44) |
| 1.34 (0.75,1.93) | 0.41 (-0.23,1.05) | CBT | 0.35 (-0.21,0.92) | 0.35 (-1.07,1.77) |
| 0.98 (0.42,1.55) | 0.06 (-0.74,0.85) | -0.35 (-0.92,0.21) | PT | -0.00 (-1.41,1.41) |
| 0.99 (-0.31,2.28) | 0.06 (-1.44,1.56) | -0.35 (-1.77,1.07) | 0.00 (-1.41,1.41) | AT |

**Notes: CON:**Control group **VRET:**Virtual Reality Exposure Therapy **CBT:**Cognitive behavioral therapy **ACT:**Acceptance and Commitment Therapy **RT:**Relaxation therapy **SIS:**Social and Interpersonal Skills **CT**:Combination therapy **PT:**Psychotherapy **AT:**Attention training **READ:**Reading therapy

**Supplementary S12.36 –** League table for **Intervention duration of ≥ 8 weeks**

| CON | -0.84 (-1.15,-0.53) | -0.79 (-0.92,-0.67) | 0.21 (-0.46,0.88) | -0.50 (-0.74,-0.25) | -0.70 (-1.01,-0.40) | -0.83 (-1.11,-0.55) | -0.68 (-0.89,-0.46) | -0.99 (-1.94,-0.04) | -0.72 (-1.05,-0.39) |
| --- | --- | --- | --- | --- | --- | --- | --- | --- | --- |
| 0.84 (0.53,1.15) | VRET | 0.05 (-0.25,0.34) | 1.05 (0.32,1.78) | 0.35 (-0.02,0.71) | 0.14 (-0.24,0.52) | 0.01 (-0.38,0.41) | 0.16 (-0.19,0.52) | -0.14 (-1.14,0.86) | 0.12 (-0.32,0.56) |
| 0.79 (0.67,0.92) | -0.05 (-0.34,0.25) | CBT | 1.00 (0.33,1.67) | 0.30 (0.07,0.53) | 0.09 (-0.20,0.39) | -0.03 (-0.31,0.24) | 0.12 (-0.09,0.33) | -0.19 (-1.15,0.77) | 0.07 (-0.26,0.41) |
| -0.21 (-0.88,0.46) | -1.05 (-1.78,-0.32) | -1.00 (-1.67,-0.33) | ACT | -0.70 (-1.41,0.00) | -0.91 (-1.64,-0.18) | -1.04 (-1.76,-0.32) | -0.89 (-1.58,-0.19) | -1.19 (-2.36,-0.03) | -0.93 (-1.67,-0.19) |
| 0.50 (0.25,0.74) | -0.35 (-0.71,0.02) | -0.30 (-0.53,-0.07) | 0.70 (-0.00,1.41) | RT | -0.21 (-0.57,0.16) | -0.33 (-0.68,0.01) | -0.18 (-0.49,0.12) | -0.49 (-1.47,0.49) | -0.22 (-0.61,0.16) |
| 0.70 (0.40,1.01) | -0.14 (-0.52,0.24) | -0.09 (-0.39,0.20) | 0.91 (0.18,1.64) | 0.21 (-0.16,0.57) | SIS | -0.13 (-0.51,0.26) | 0.02 (-0.33,0.38) | -0.28 (-1.28,0.71) | -0.02 (-0.46,0.42) |
| 0.83 (0.55,1.11) | -0.01 (-0.41,0.38) | 0.03 (-0.24,0.31) | 1.04 (0.32,1.76) | 0.33 (-0.01,0.68) | 0.13 (-0.26,0.51) | CT | 0.15 (-0.17,0.47) | -0.16 (-1.15,0.83) | 0.11 (-0.28,0.50) |
| 0.68 (0.46,0.89) | -0.16 (-0.52,0.19) | -0.12 (-0.33,0.09) | 0.89 (0.19,1.58) | 0.18 (-0.12,0.49) | -0.02 (-0.38,0.33) | -0.15 (-0.47,0.17) | PT | -0.31 (-1.28,0.67) | -0.04 (-0.41,0.33) |
| 0.99 (0.04,1.94) | 0.14 (-0.86,1.14) | 0.19 (-0.77,1.15) | 1.19 (0.03,2.36) | 0.49 (-0.49,1.47) | 0.28 (-0.71,1.28) | 0.16 (-0.83,1.15) | 0.31 (-0.67,1.28) | AT | 0.27 (-0.74,1.27) |
| 0.72 (0.39,1.05) | -0.12 (-0.56,0.32) | -0.07 (-0.41,0.26) | 0.93 (0.19,1.67) | 0.22 (-0.16,0.61) | 0.02 (-0.42,0.46) | -0.11 (-0.50,0.28) | 0.04 (-0.33,0.41) | -0.27 (-1.27,0.74) | READ |

**Notes: CON:**Control group **VRET:**Virtual Reality Exposure Therapy **CBT:**Cognitive behavioral therapy **ACT:**Acceptance and Commitment Therapy **RT:**Relaxation therapy **SIS:**Social and Interpersonal Skills **CT**:Combination therapy **PT:**Psychotherapy **AT:**Attention training **READ:**Reading therapy

**Supplementary S12.37 –** League table for **Intervention duration of ＜ 8 weeks**

| CON | -0.53 (-0.97,-0.08) | -0.83 (-1.12,-0.54) | -0.53 (-1.02,-0.03) | -0.44 (-1.04,0.17) | -0.39 (-1.01,0.23) | -0.80 (-1.20,-0.40) | -0.35 (-0.70,-0.01) | -0.22 (-1.66,1.23) |
| --- | --- | --- | --- | --- | --- | --- | --- | --- |
| 0.53 (0.08,0.97) | VRET | -0.30 (-0.78,0.18) | 0.00 (-0.65,0.65) | 0.09 (-0.63,0.82) | 0.13 (-0.58,0.85) | -0.27 (-0.84,0.29) | 0.18 (-0.34,0.69) | 0.31 (-1.19,1.81) |
| 0.83 (0.54,1.12) | 0.30 (-0.18,0.78) | CBT | 0.30 (-0.23,0.84) | 0.39 (-0.20,0.99) | 0.44 (-0.19,1.06) | 0.03 (-0.34,0.40) | 0.48 (0.12,0.83) | 0.61 (-0.82,2.05) |
| 0.53 (0.03,1.02) | -0.00 (-0.65,0.65) | -0.30 (-0.84,0.23) | ACT | 0.09 (-0.68,0.85) | 0.13 (-0.65,0.91) | -0.28 (-0.89,0.34) | 0.17 (-0.41,0.76) | 0.31 (-1.21,1.83) |
| 0.44 (-0.17,1.04) | -0.09 (-0.82,0.63) | -0.39 (-0.99,0.20) | -0.09 (-0.85,0.68) | RT | 0.04 (-0.80,0.88) | -0.36 (-1.01,0.28) | 0.09 (-0.53,0.70) | 0.22 (-1.31,1.75) |
| 0.39 (-0.23,1.01) | -0.13 (-0.85,0.58) | -0.44 (-1.06,0.19) | -0.13 (-0.91,0.65) | -0.04 (-0.88,0.80) | CT | -0.41 (-1.11,0.30) | 0.04 (-0.64,0.72) | 0.18 (-1.38,1.73) |
| 0.80 (0.40,1.20) | 0.27 (-0.29,0.84) | -0.03 (-0.40,0.34) | 0.28 (-0.34,0.89) | 0.36 (-0.28,1.01) | 0.41 (-0.30,1.11) | PT | 0.45 (-0.03,0.93) | 0.59 (-0.80,1.97) |
| 0.35 (0.01,0.70) | -0.18 (-0.69,0.34) | -0.48 (-0.83,-0.12) | -0.17 (-0.76,0.41) | -0.09 (-0.70,0.53) | -0.04 (-0.72,0.64) | -0.45 (-0.93,0.03) | AT | 0.14 (-1.33,1.60) |
| 0.22 (-1.23,1.66) | -0.31 (-1.81,1.19) | -0.61 (-2.05,0.82) | -0.31 (-1.83,1.21) | -0.22 (-1.75,1.31) | -0.18 (-1.73,1.38) | -0.59 (-1.97,0.80) | -0.14 (-1.60,1.33) | READ |

**Notes: CON:**Control group **VRET:**Virtual Reality Exposure Therapy **CBT:**Cognitive behavioral therapy **ACT:**Acceptance and Commitment Therapy **RT:**Relaxation therapy **SIS:**Social and Interpersonal Skills **CT**:Combination therapy **PT:**Psychotherapy **AT:**Attention training **READ:**Reading therapy

**Supplementary S12.38 –** League table for **Subclinical baseline severity**

| CON | -1.36 (-2.91,0.18) | -1.05 (-1.80,-0.29) | -1.18 (-2.19,-0.18) |
| --- | --- | --- | --- |
| 1.36 (-0.18,2.91) | VRET | 0.32 (-1.03,1.66) | 0.18 (-1.50,1.86) |
| 1.05 (0.29,1.80) | -0.32 (-1.66,1.03) | CBT | -0.14 (-1.14,0.87) |
| 1.18 (0.18,2.19) | -0.18 (-1.86,1.50) | 0.14 (-0.87,1.14) | PT |

**Notes: CON:**Control group **VRET:**Virtual Reality Exposure Therapy **CBT:**Cognitive behavioral therapy **PT:**Psychotherapy

**Supplementary S12.39 –** League table for **Moderate to Severe baseline severity**

| CON | -0.33 (-0.78,0.12) | -0.65 (-0.81,-0.49) | -0.42 (-0.76,-0.08) | -0.46 (-0.75,-0.17) | -0.53 (-0.98,-0.08) | -0.39 (-0.74,-0.05) | -0.53 (-0.81,-0.25) |
| --- | --- | --- | --- | --- | --- | --- | --- |
| 0.33 (-0.12,0.78) | VRET | -0.32 (-0.77,0.12) | -0.09 (-0.64,0.46) | -0.13 (-0.67,0.41) | -0.20 (-0.81,0.41) | -0.07 (-0.59,0.46) | -0.20 (-0.73,0.33) |
| 0.65 (0.49,0.81) | 0.32 (-0.12,0.77) | CBT | 0.23 (-0.10,0.57) | 0.19 (-0.08,0.47) | 0.12 (-0.30,0.54) | 0.26 (-0.04,0.55) | 0.12 (-0.17,0.42) |
| 0.42 (0.08,0.76) | 0.09 (-0.46,0.64) | -0.23 (-0.57,0.10) | ACT | -0.04 (-0.46,0.38) | -0.11 (-0.64,0.42) | 0.03 (-0.42,0.47) | -0.11 (-0.54,0.32) |
| 0.46 (0.17,0.75) | 0.13 (-0.41,0.67) | -0.19 (-0.47,0.08) | 0.04 (-0.38,0.46) | RT | -0.07 (-0.57,0.43) | 0.06 (-0.35,0.48) | -0.07 (-0.44,0.29) |
| 0.53 (0.08,0.98) | 0.20 (-0.41,0.81) | -0.12 (-0.54,0.30) | 0.11 (-0.42,0.64) | 0.07 (-0.43,0.57) | CT | 0.14 (-0.38,0.65) | -0.00 (-0.51,0.51) |
| 0.39 (0.05,0.74) | 0.07 (-0.46,0.59) | -0.26 (-0.55,0.04) | -0.03 (-0.47,0.42) | -0.06 (-0.48,0.35) | -0.14 (-0.65,0.38) | PT | -0.14 (-0.56,0.29) |
| 0.53 (0.25,0.81) | 0.20 (-0.33,0.73) | -0.12 (-0.42,0.17) | 0.11 (-0.32,0.54) | 0.07 (-0.29,0.44) | 0.00 (-0.51,0.51) | 0.14 (-0.29,0.56) | AT |

**Notes: CON:**Control group **VRET:**Virtual Reality Exposure Therapy **CBT:**Cognitive behavioral therapy **ACT:**Acceptance and Commitment Therapy **RT:**Relaxation therapy **CT**:Combination therapy **PT:**Psychotherapy **AT:**Attention training

**Supplementary S12.40 –** League table for S**evere baseline severity**

| CON | -0.72 (-1.01,-0.42) | -0.83 (-0.98,-0.68) | -0.15 (-0.70,0.41) | -0.47 (-0.75,-0.18) | -0.70 (-1.03,-0.36) | -0.80 (-1.11,-0.49) | -0.73 (-0.96,-0.51) | -0.36 (-0.68,-0.03) | -0.69 (-1.05,-0.32) |
| --- | --- | --- | --- | --- | --- | --- | --- | --- | --- |
| 0.72 (0.42,1.01) | VRET | -0.11 (-0.40,0.18) | 0.57 (-0.05,1.19) | 0.25 (-0.13,0.62) | 0.02 (-0.38,0.42) | -0.09 (-0.48,0.31) | -0.02 (-0.36,0.33) | 0.36 (-0.05,0.76) | 0.03 (-0.42,0.48) |
| 0.83 (0.68,0.98) | 0.11 (-0.18,0.40) | CBT | 0.68 (0.12,1.24) | 0.36 (0.09,0.64) | 0.13 (-0.19,0.46) | 0.03 (-0.27,0.33) | 0.10 (-0.12,0.32) | 0.47 (0.14,0.80) | 0.14 (-0.22,0.51) |
| 0.15 (-0.41,0.70) | -0.57 (-1.19,0.05) | -0.68 (-1.24,-0.12) | ACT | -0.32 (-0.94,0.29) | -0.55 (-1.19,0.09) | -0.66 (-1.28,-0.03) | -0.59 (-1.18,0.01) | -0.21 (-0.85,0.42) | -0.54 (-1.20,0.12) |
| 0.47 (0.18,0.75) | -0.25 (-0.62,0.13) | -0.36 (-0.64,-0.09) | 0.32 (-0.29,0.94) | RT | -0.23 (-0.64,0.19) | -0.33 (-0.72,0.05) | -0.26 (-0.60,0.07) | 0.11 (-0.28,0.50) | -0.22 (-0.65,0.21) |
| 0.70 (0.36,1.03) | -0.02 (-0.42,0.38) | -0.13 (-0.46,0.19) | 0.55 (-0.09,1.19) | 0.23 (-0.19,0.64) | SIS | -0.11 (-0.53,0.32) | -0.04 (-0.42,0.35) | 0.34 (-0.11,0.79) | 0.01 (-0.47,0.49) |
| 0.80 (0.49,1.11) | 0.09 (-0.31,0.48) | -0.03 (-0.33,0.27) | 0.66 (0.03,1.28) | 0.33 (-0.05,0.72) | 0.11 (-0.32,0.53) | CT | 0.07 (-0.27,0.41) | 0.44 (0.01,0.87) | 0.12 (-0.31,0.54) |
| 0.73 (0.51,0.96) | 0.02 (-0.33,0.36) | -0.10 (-0.32,0.12) | 0.59 (-0.01,1.18) | 0.26 (-0.07,0.60) | 0.04 (-0.35,0.42) | -0.07 (-0.41,0.27) | PT | 0.37 (-0.01,0.75) | 0.05 (-0.35,0.44) |
| 0.36 (0.03,0.68) | -0.36 (-0.76,0.05) | -0.47 (-0.80,-0.14) | 0.21 (-0.42,0.85) | -0.11 (-0.50,0.28) | -0.34 (-0.79,0.11) | -0.44 (-0.87,-0.01) | -0.37 (-0.75,0.01) | AT | -0.33 (-0.80,0.15) |
| 0.69 (0.32,1.05) | -0.03 (-0.48,0.42) | -0.14 (-0.51,0.22) | 0.54 (-0.12,1.20) | 0.22 (-0.21,0.65) | -0.01 (-0.49,0.47) | -0.12 (-0.54,0.31) | -0.05 (-0.44,0.35) | 0.33 (-0.15,0.80) | READ |

**Notes: CON:**Control group **VRET:**Virtual Reality Exposure Therapy **CBT:**Cognitive behavioral therapy **ACT:**Acceptance and Commitment Therapy **RT:**Relaxation therapy **SIS:**Social and Interpersonal Skills **CT**:Combination therapy **PT:**Psychotherapy **AT:**Attention training **READ:**Reading therapy

**Supplementary S12.41 –** League table for **Individual face-to-face format**

| CON | -0.97 (-1.31,-0.62) | -1.03 (-1.27,-0.80) | -0.27 (-0.69,0.16) | -0.38 (-0.86,0.10) | -0.56 (-1.01,-0.12) | -0.77 (-1.11,-0.44) | -1.00 (-1.64,-0.35) | -0.19 (-1.37,1.00) |
| --- | --- | --- | --- | --- | --- | --- | --- | --- |
| 0.97 (0.62,1.31) | VRET | -0.07 (-0.38,0.25) | 0.70 (0.17,1.23) | 0.59 (0.08,1.10) | 0.40 (-0.12,0.92) | 0.19 (-0.23,0.62) | -0.03 (-0.70,0.64) | 0.78 (-0.44,1.99) |
| 1.03 (0.80,1.27) | 0.07 (-0.25,0.38) | CBT | 0.77 (0.31,1.23) | 0.66 (0.19,1.12) | 0.47 (0.01,0.93) | 0.26 (-0.04,0.56) | 0.04 (-0.63,0.71) | 0.85 (-0.33,2.02) |
| 0.27 (-0.16,0.69) | -0.70 (-1.23,-0.17) | -0.77 (-1.23,-0.31) | ACT | -0.11 (-0.74,0.52) | -0.30 (-0.90,0.31) | -0.51 (-1.04,0.03) | -0.73 (-1.50,0.04) | 0.08 (-1.18,1.34) |
| 0.38 (-0.10,0.86) | -0.59 (-1.10,-0.08) | -0.66 (-1.12,-0.19) | 0.11 (-0.52,0.74) | RT | -0.19 (-0.81,0.44) | -0.39 (-0.94,0.15) | -0.62 (-1.40,0.17) | 0.19 (-1.07,1.45) |
| 0.56 (0.12,1.01) | -0.40 (-0.92,0.12) | -0.47 (-0.93,-0.01) | 0.30 (-0.31,0.90) | 0.19 (-0.44,0.81) | SIS | -0.21 (-0.74,0.32) | -0.43 (-1.21,0.34) | 0.38 (-0.88,1.63) |
| 0.77 (0.44,1.11) | -0.19 (-0.62,0.23) | -0.26 (-0.56,0.04) | 0.51 (-0.03,1.04) | 0.39 (-0.15,0.94) | 0.21 (-0.32,0.74) | PT | -0.22 (-0.94,0.49) | 0.59 (-0.55,1.72) |
| 1.00 (0.35,1.64) | 0.03 (-0.64,0.70) | -0.04 (-0.71,0.63) | 0.73 (-0.04,1.50) | 0.62 (-0.17,1.40) | 0.43 (-0.34,1.21) | 0.22 (-0.49,0.94) | AT | 0.81 (-0.54,2.15) |
| 0.19 (-1.00,1.37) | -0.78 (-1.99,0.44) | -0.85 (-2.02,0.33) | -0.08 (-1.34,1.18) | -0.19 (-1.45,1.07) | -0.38 (-1.63,0.88) | -0.59 (-1.72,0.55) | -0.81 (-2.15,0.54) | READ |

**Notes: CON:**Control group **VRET:**Virtual Reality Exposure Therapy **CBT:**Cognitive behavioral therapy **ACT:**Acceptance and Commitment Therapy **RT:**Relaxation therapy **SIS:**Social and Interpersonal Skills **PT:**Psychotherapy **AT:**Attention training **READ:**Reading therapy

**Supplementary S12.42 –** League table for **Group face-to-face format**

| CON | -0.58 (-1.23,0.06) | -0.85 (-1.15,-0.55) | -0.53 (-1.05,-0.02) | -0.66 (-1.21,-0.11) | -0.80 (-1.19,-0.42) |
| --- | --- | --- | --- | --- | --- |
| 0.58 (-0.06,1.23) | VRET | -0.26 (-0.85,0.33) | 0.05 (-0.70,0.80) | -0.07 (-0.83,0.68) | -0.22 (-0.89,0.46) |
| 0.85 (0.55,1.15) | 0.26 (-0.33,0.85) | CBT | 0.31 (-0.16,0.78) | 0.19 (-0.35,0.73) | 0.05 (-0.30,0.39) |
| 0.53 (0.02,1.05) | -0.05 (-0.80,0.70) | -0.31 (-0.78,0.16) | RT | -0.12 (-0.83,0.58) | -0.27 (-0.84,0.30) |
| 0.66 (0.11,1.21) | 0.07 (-0.68,0.83) | -0.19 (-0.73,0.35) | 0.12 (-0.58,0.83) | CT | -0.14 (-0.75,0.46) |
| 0.80 (0.42,1.19) | 0.22 (-0.46,0.89) | -0.05 (-0.39,0.30) | 0.27 (-0.30,0.84) | 0.14 (-0.46,0.75) | PT |

**Notes: CON:**Control group **VRET:**Virtual Reality Exposure Therapy **CBT:**Cognitive behavioral therapy **RT:**Relaxation therapy **CT**:Combination therapy **PT:**Psychotherapy

**Supplementary S12.43 –** League table for Online **format**

| CON | -0.70 (-0.85,-0.55) | -0.46 (-0.84,-0.07) | -0.33 (-0.71,0.04) | -0.07 (-0.85,0.70) | -0.55 (-1.06,-0.05) | -0.48 (-0.89,-0.08) | -0.37 (-0.63,-0.12) | -0.77 (-1.21,-0.32) |
| --- | --- | --- | --- | --- | --- | --- | --- | --- |
| 0.70 (0.55,0.85) | CBT | 0.24 (-0.15,0.64) | 0.37 (0.00,0.73) | 0.63 (-0.13,1.39) | 0.15 (-0.36,0.65) | 0.22 (-0.19,0.63) | 0.33 (0.07,0.58) | -0.06 (-0.50,0.37) |
| 0.46 (0.07,0.84) | -0.24 (-0.64,0.15) | ACT | 0.12 (-0.40,0.65) | 0.38 (-0.47,1.24) | -0.10 (-0.72,0.53) | -0.03 (-0.58,0.53) | 0.08 (-0.37,0.54) | -0.31 (-0.89,0.27) |
| 0.33 (-0.04,0.71) | -0.37 (-0.73,-0.00) | -0.12 (-0.65,0.40) | RT | 0.26 (-0.58,1.11) | -0.22 (-0.78,0.34) | -0.15 (-0.65,0.35) | -0.04 (-0.44,0.36) | -0.43 (-0.95,0.08) |
| 0.07 (-0.70,0.85) | -0.63 (-1.39,0.13) | -0.38 (-1.24,0.47) | -0.26 (-1.11,0.58) | SIS | -0.48 (-1.39,0.43) | -0.41 (-1.28,0.45) | -0.30 (-1.11,0.50) | -0.69 (-1.57,0.18) |
| 0.55 (0.05,1.06) | -0.15 (-0.65,0.36) | 0.10 (-0.53,0.72) | 0.22 (-0.34,0.78) | 0.48 (-0.43,1.39) | CT | 0.07 (-0.56,0.70) | 0.18 (-0.37,0.73) | -0.21 (-0.80,0.37) |
| 0.48 (0.08,0.89) | -0.22 (-0.63,0.19) | 0.03 (-0.53,0.58) | 0.15 (-0.35,0.65) | 0.41 (-0.45,1.28) | -0.07 (-0.70,0.56) | PT | 0.11 (-0.36,0.58) | -0.28 (-0.87,0.30) |
| 0.37 (0.12,0.63) | -0.33 (-0.58,-0.07) | -0.08 (-0.54,0.37) | 0.04 (-0.36,0.44) | 0.30 (-0.50,1.11) | -0.18 (-0.73,0.37) | -0.11 (-0.58,0.36) | AT | -0.39 (-0.88,0.10) |
| 0.77 (0.32,1.21) | 0.06 (-0.37,0.50) | 0.31 (-0.27,0.89) | 0.43 (-0.08,0.95) | 0.69 (-0.18,1.57) | 0.21 (-0.37,0.80) | 0.28 (-0.30,0.87) | 0.39 (-0.10,0.88) | READ |

**Notes: CON:**Control group **CBT:**Cognitive behavioral therapy **ACT:**Acceptance and Commitment Therapy **RT:**Relaxation therapy **SIS:**Social and Interpersonal Skills **CT**:Combination therapy **PT:**Psychotherapy **AT:**Attention training **READ:**Reading therapy

**Supplementary S12.44 –** League table for Mixed **format**

| CON | -0.73 (-1.08,-0.38) | -0.72 (-1.02,-0.42) | -0.74 (-1.54,0.07) | -0.86 (-1.25,-0.47) | -0.81 (-1.21,-0.41) | -0.50 (-0.94,-0.07) | 0.56 (-0.25,1.36) | -0.53 (-0.93,-0.13) |
| --- | --- | --- | --- | --- | --- | --- | --- | --- |
| 0.73 (0.38,1.08) | VRET | 0.01 (-0.42,0.45) | -0.01 (-0.88,0.87) | -0.13 (-0.59,0.32) | -0.08 (-0.59,0.42) | 0.23 (-0.32,0.78) | 1.29 (0.41,2.17) | 0.20 (-0.33,0.73) |
| 0.72 (0.42,1.02) | -0.01 (-0.45,0.42) | CBT | -0.02 (-0.84,0.80) | -0.15 (-0.50,0.20) | -0.09 (-0.45,0.26) | 0.21 (-0.20,0.63) | 1.27 (0.41,2.14) | 0.19 (-0.27,0.64) |
| 0.74 (-0.07,1.54) | 0.01 (-0.87,0.88) | 0.02 (-0.80,0.84) | RT | -0.13 (-1.00,0.75) | -0.07 (-0.95,0.80) | 0.23 (-0.66,1.13) | 1.30 (0.15,2.44) | 0.21 (-0.68,1.10) |
| 0.86 (0.47,1.25) | 0.13 (-0.32,0.59) | 0.15 (-0.20,0.50) | 0.13 (-0.75,1.00) | SIS | 0.05 (-0.40,0.51) | 0.36 (-0.15,0.87) | 1.42 (0.52,2.32) | 0.33 (-0.19,0.86) |
| 0.81 (0.41,1.21) | 0.08 (-0.42,0.59) | 0.09 (-0.26,0.45) | 0.07 (-0.80,0.95) | -0.05 (-0.51,0.40) | CT | 0.31 (-0.16,0.78) | 1.37 (0.47,2.27) | 0.28 (-0.20,0.77) |
| 0.50 (0.07,0.94) | -0.23 (-0.78,0.32) | -0.21 (-0.63,0.20) | -0.23 (-1.13,0.66) | -0.36 (-0.87,0.15) | -0.31 (-0.78,0.16) | PT | 1.06 (0.14,1.98) | -0.03 (-0.53,0.48) |
| -0.56 (-1.36,0.25) | -1.29 (-2.17,-0.41) | -1.27 (-2.14,-0.41) | -1.30 (-2.44,-0.15) | -1.42 (-2.32,-0.52) | -1.37 (-2.27,-0.47) | -1.06 (-1.98,-0.14) | AT | -1.09 (-1.99,-0.18) |
| 0.53 (0.13,0.93) | -0.20 (-0.73,0.33) | -0.19 (-0.64,0.27) | -0.21 (-1.10,0.68) | -0.33 (-0.86,0.19) | -0.28 (-0.77,0.20) | 0.03 (-0.48,0.53) | 1.09 (0.18,1.99) | READ |

**Notes: CON:**Control group **VRET:**Virtual Reality Exposure Therapy **CBT:**Cognitive behavioral therapy **RT:**Relaxation therapy **SIS:**Social and Interpersonal Skills **CT**:Combination therapy **PT:**Psychotherapy **AT:**Attention training **READ:**Reading therapy

**Supplementary S12.45** – Surface Under the Cumulative Ranking curve Score and Ranking for **developed country**

| ****Developed Country**** | | | |
| --- | --- | --- | --- |
| Treatment | SUCRA | PrBest | Mean Rank |
| CON | 0.3 | 0.0 | 10.0 |
| VRET | 77.5 | 30.8 | 3.0 |
| CBT | 81.4 | 15.8 | 2.7 |
| ACT | 22.2 | 0.2 | 8.0 |
| RT | 31.0 | 0.0 | 7.2 |
| SIS | 63.0 | 12 | 4.3 |
| CT | 71.7 | 17.2 | 3.5 |
| PT | 68.2 | 10.0 | 3.9 |
| AT | 22.2 | 0.0 | 8.0 |
| READ | 62.2 | 13.9 | 4.4 |

**Notes: CON:**Control group **VRET:**Virtual Reality Exposure Therapy **CBT:**Cognitive behavioral therapy **ACT:**Acceptance and Commitment Therapy **RT:**Relaxation therapy **SIS:**Social and Interpersonal Skills **CT**:Combination therapy **PT:**Psychotherapy **AT:**Attention training **READ:**Reading therapy

**Supplementary S12.46** – Surface Under the Cumulative Ranking curve Score and Ranking for **developing country**

| ****Developing Country**** | | | |
| --- | --- | --- | --- |
| Treatment | SUCRA | PrBest | Mean Rank |
| CON | 1.9 | 0.0 | 4.9 |
| VRET | 50.7 | 7.0 | 3.0 |
| CBT | 87.0 | 56.7 | 1.5 |
| PT | 53.8 | 6.1 | 2.8 |
| AT | 57.0 | 30.2 | 2.7 |

**Notes: CON:**Control group **VRET:**Virtual Reality Exposure Therapy **CBT:**Cognitive behavioral therapy **PT:**Psychotherapy **AT:**Attention training

**Supplementary S12.47** – Surface Under the Cumulative Ranking curve Score and Ranking for **Intervention duration of ≥ 8 weeks**

| ****Intervention duration of ≥ 8 weeks**** | | | |
| --- | --- | --- | --- |
| Treatment | SUCRA | PrBest | Mean Rank |
| CON | 8.3 | 0.0 | 9.3 |
| VRET | 75.6 | 18.1 | 3.2 |
| CBT | 71.0 | 3.4 | 3.6 |
| ACT | 3.9 | 0.1 | 9.6 |
| RT | 28.7 | 0.0 | 7.4 |
| SIS | 54.8 | 3.8 | 5.1 |
| CT | 74.0 | 14.0 | 3.3 |
| PT | 50.0 | 1.0 | 5.5 |
| AT | 75.9 | 53.1 | 3.2 |
| READ | 57.9 | 6.5 | 4.8 |

**Notes: CON:**Control group **VRET:**Virtual Reality Exposure Therapy **CBT:**Cognitive behavioral therapy **ACT:**Acceptance and Commitment Therapy **RT:**Relaxation therapy **SIS:**Social and Interpersonal Skills **CT**:Combination therapy **PT:**Psychotherapy **AT:**Attention training **READ:**Reading therapy

**Supplementary S12.48** – Surface Under the Cumulative Ranking curve Score and Ranking for **Intervention duration of ＜ 8 weeks**

| ****Intervention duration of ＜ 8 weeks**** | | | |
| --- | --- | --- | --- |
| Treatment | SUCRA | PrBest | Mean Rank |
| CON | 7.7 | 0 | 8.4 |
| VRET | 55.4 | 5.3 | 4.6 |
| CBT | 86.5 | 32.6 | 2.1 |
| ACT | 55 | 6.7 | 4.6 |
| RT | 46.3 | 4.9 | 5.3 |
| CT | 43 | 4.9 | 5.6 |
| PT | 82.2 | 29.2 | 2.4 |
| AT | 37 | 0.1 | 6 |
| READ | 36.8 | 16.4 | 6.1 |

**Notes: CON:**Control group **VRET:**Virtual Reality Exposure Therapy **CBT:**Cognitive behavioral therapy **ACT:**Acceptance and Commitment Therapy **RT:**Relaxation therapy **CT**:Combination therapy **PT:**Psychotherapy **AT:**Attention training **READ:**Reading therapy

**Supplementary S12.49** – Surface Under the Cumulative Ranking curve Score and Ranking for Subclinical baseline severity

| **Subclinical baseline severity** | | | |
| --- | --- | --- | --- |
| Treatment | SUCRA | PrBest | Mean Rank |
| CON | 1.80 | 0.0 | 3.9 |
| VRET | 74.2 | 52.6 | 1.8 |
| CBT | 56.8 | 12.8 | 2.3 |
| PT | 67.2 | 34.6 | 2.0 |

**Notes: CON:**Control group **VRET:**Virtual Reality Exposure Therapy **CBT:**Cognitive behavioral therapy **PT:**Psychotherapy

**Supplementary S12.50** – Surface Under the Cumulative Ranking curve Score and Ranking for Moderate to Severe baseline severity

| ****Moderate to Severe baseline severity**** | | | |
| --- | --- | --- | --- |
| Treatment | SUCRA | PrBest | Mean Rank |
| CON | 1.5 | 0 | 7.9 |
| VRET | 36.9 | 5.5 | 5.4 |
| CBT | 88.9 | 42.8 | 1.8 |
| ACT | 47.1 | 5.3 | 4.7 |
| RT | 52.8 | 4.9 | 4.3 |
| CT | 64 | 24 | 3.5 |
| PT | 43.5 | 2.7 | 5 |
| AT | 65.3 | 14.8 | 3.4 |

**Notes: CON:**Control group **VRET:**Virtual Reality Exposure Therapy **CBT:**Cognitive behavioral therapy **ACT:**Acceptance and Commitment Therapy **RT:**Relaxation therapy **CT**:Combination therapy **PT:**Psychotherapy **AT:**Attention training

**Supplementary S12.51** – Surface Under the Cumulative Ranking curve Score and Ranking for Severe baseline severity

| ****Severe baseline severity**** | | | |
| --- | --- | --- | --- |
| Treatment | SUCRA | PrBest | Mean Rank |
| CON | 3.6 | 0 | 9.7 |
| VRET | 66.2 | 11.5 | 4 |
| CBT | 85.7 | 26.3 | 2.3 |
| ACT | 14.2 | 0.3 | 8.7 |
| RT | 34 | 0.1 | 6.9 |
| SIS | 63 | 11.4 | 4.3 |
| CT | 78.3 | 29.7 | 3 |
| PT | 67.9 | 7.9 | 3.9 |
| AT | 25.4 | 0.1 | 7.7 |
| READ | 61.9 | 12.8 | 4.4 |

**Notes: CON:**Control group **VRET:**Virtual Reality Exposure Therapy **CBT:**Cognitive behavioral therapy **ACT:**Acceptance and Commitment Therapy **RT:**Relaxation therapy **SIS:**Social and Interpersonal Skills **CT**:Combination therapy **PT:**Psychotherapy **AT:**Attention training **READ:**Reading therapy

**Supplementary S12.52** – Surface Under the Cumulative Ranking curve Score and Ranking for Individual face-to-face format

| ****Individual face-to-face format**** | | | |
| --- | --- | --- | --- |
| Treatment | SUCRA | PrBest | Mean Rank |
| CON | 6.8 | 0 | 8.5 |
| VRET | 80.5 | 16.9 | 2.6 |
| CBT | 88.3 | 35.2 | 1.9 |
| ACT | 25.6 | 0 | 7 |
| RT | 32.8 | 0.1 | 6.4 |
| SIS | 46.1 | 0.9 | 5.3 |
| PT | 62.8 | 2 | 4 |
| AT | 79.6 | 39.4 | 2.6 |
| READ | 27.6 | 5.6 | 6.8 |

**Notes: CON:**Control group **VRET:**Virtual Reality Exposure Therapy **CBT:**Cognitive behavioral therapy **ACT:**Acceptance and Commitment Therapy **RT:**Relaxation therapy **SIS:**Social and Interpersonal Skills **PT:**Psychotherapy **AT:**Attention training **READ:**Reading therapy

**Supplementary S12.53** – Surface Under the Cumulative Ranking curve Score and Ranking for Group face-to-face format

| ****Group face-to-face format**** | | | |
| --- | --- | --- | --- |
| Treatment | SUCRA | PrBest | Mean Rank |
| CON | 1.4 | 0 | 5.9 |
| VRET | 47.9 | 13.3 | 3.6 |
| CBT | 81.5 | 35.7 | 1.9 |
| RT | 41.4 | 5.9 | 3.9 |
| CT | 54.9 | 16.7 | 3.3 |
| PT | 72.8 | 28.4 | 2.4 |

**Notes: CON:**Control group **VRET:**Virtual Reality Exposure Therapy **CBT:**Cognitive behavioral therapy **RT:**Relaxation therapy **CT**:Combination therapy **PT:**Psychotherapy

**Supplementary S12.54** – Surface Under the Cumulative Ranking curve Score and Ranking for Online format

| ****Online format**** | | | |
| --- | --- | --- | --- |
| Treatment | SUCRA | PrBest | Mean Rank |
| CON | 6.2 | 0 | 8.5 |
| CBT | 84.2 | 22.3 | 2.3 |
| ACT | 52.9 | 4.5 | 4.8 |
| RT | 37.1 | 0.4 | 6 |
| SIS | 21.7 | 3 | 7.3 |
| CT | 63.8 | 14.3 | 3.9 |
| PT | 56.1 | 6.4 | 4.5 |
| AT | 42 | 0.2 | 5.6 |
| READ | 86 | 48.8 | 2.1 |

**Notes: CON:**Control group **CBT:**Cognitive behavioral therapy **ACT:**Acceptance and Commitment Therapy **RT:**Relaxation therapy **SIS:**Social and Interpersonal Skills **CT**:Combination therapy **PT:**Psychotherapy **AT:**Attention training **READ:**Reading therapy

**Supplementary S12.55** – Surface Under the Cumulative Ranking curve Score and Ranking for Mixed format

| ****Online format**** | | | |
| --- | --- | --- | --- |
| Treatment | SUCRA | PrBest | Mean Rank |
| CON | 12.1 | 0 | 8 |
| VRET | 65.7 | 11.4 | 3.7 |
| CBT | 63.3 | 2.9 | 3.9 |
| RT | 64.4 | 29.4 | 3.8 |
| SIS | 81.5 | 31.1 | 2.5 |
| CT | 76.1 | 21.7 | 2.9 |
| PT | 41.1 | 1.3 | 5.7 |
| AT | 1.6 | 0.1 | 8.9 |
| READ | 44.3 | 2.1 | 5.5 |

**Notes: CON:**Control group **VRET:**Virtual Reality Exposure Therapy **CBT:**Cognitive behavioral therapy **RT:**Relaxation therapy **SIS:**Social and Interpersonal Skills **CT**:Combination therapy **PT:**Psychotherapy **AT:**Attention training **READ:**Reading therapy

**Supplementary S12.56** – Surface Under the Cumulative Ranking curve Probability Sorting Plot for d**eveloped country**


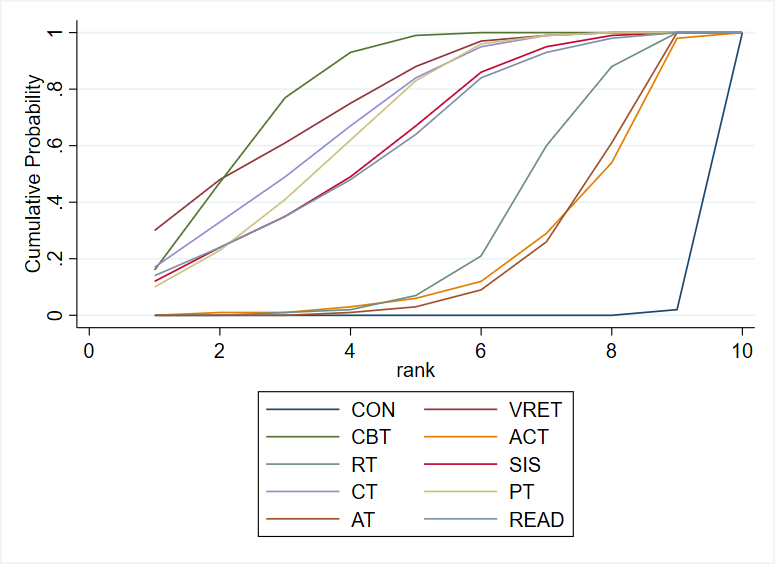


**Notes: CON:**Control group **VRET:**Virtual Reality Exposure Therapy **CBT:**Cognitive behavioral therapy **ACT:**Acceptance and Commitment Therapy **RT:**Relaxation therapy **SIS:**Social and Interpersonal Skills **CT**:Combination therapy **PT:**Psychotherapy **AT:**Attention training **READ:**Reading therapy

**Supplementary S12.57** – Surface Under the Cumulative Ranking curve Probability Sorting Plot for developing country

**
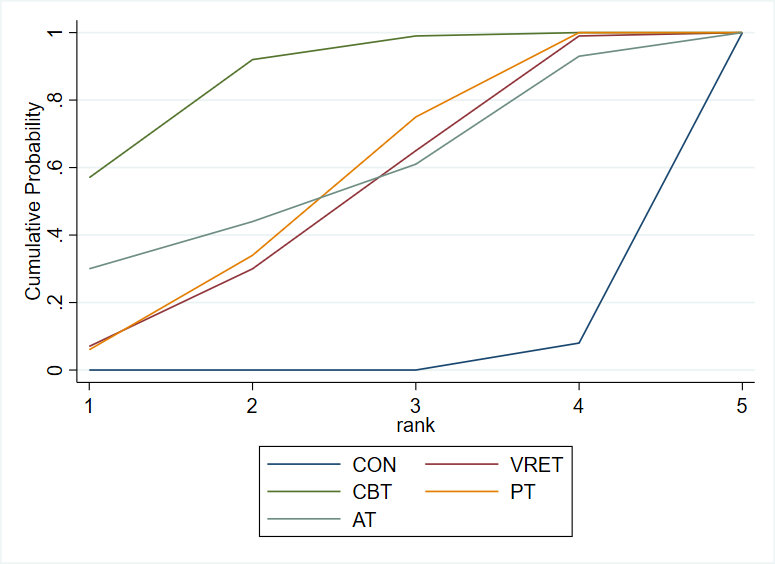
**

**Notes: CON:**Control group **VRET:**Virtual Reality Exposure Therapy **CBT:**Cognitive behavioral therapy **PT:**Psychotherapy **AT:**Attention training

**Supplementary S12.58** – Surface Under the Cumulative Ranking curve Probability Sorting Plot for **Intervention duration of ≥ 8 weeks**

**
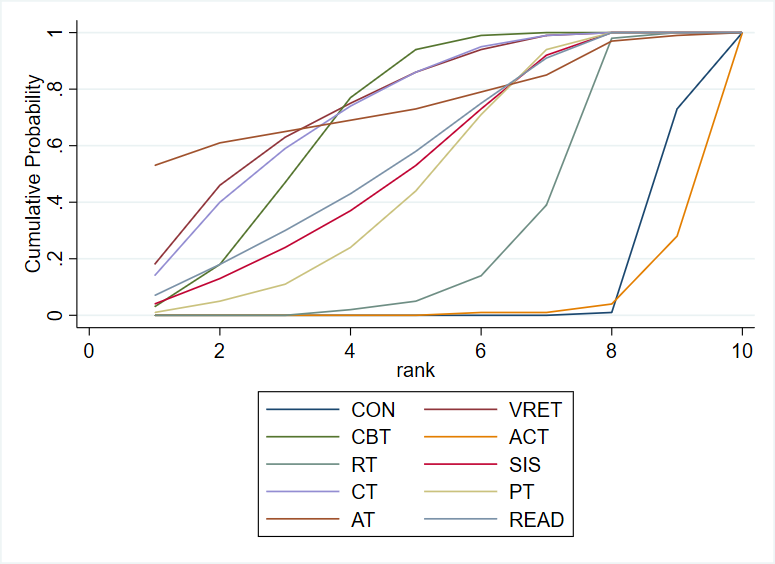
**

**Notes: CON:**Control group **VRET:**Virtual Reality Exposure Therapy **CBT:**Cognitive behavioral therapy **ACT:**Acceptance and Commitment Therapy **RT:**Relaxation therapy **SIS:**Social and Interpersonal Skills **CT**:Combination therapy **PT:**Psychotherapy **AT:**Attention training **READ:**Reading therapy

**Supplementary S12.59** – Surface Under the Cumulative Ranking curve Probability Sorting Plot for **Intervention duration of ＜ 8 weeks**

**
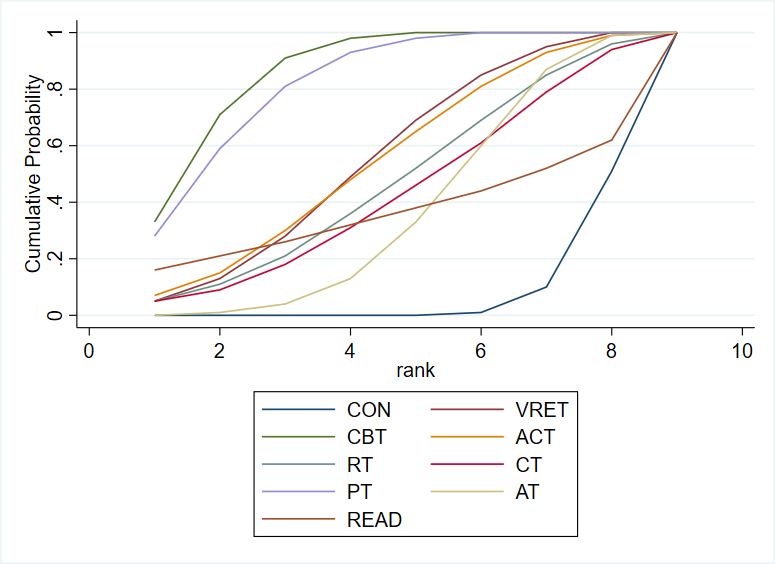
**

**Notes: CON:**Control group **VRET:**Virtual Reality Exposure Therapy **CBT:**Cognitive behavioral therapy **ACT:**Acceptance and Commitment Therapy **RT:**Relaxation therapy **CT**:Combination therapy **PT:**Psychotherapy **AT:**Attention training **READ:**Reading therapy

**Supplementary S12.60** – Surface Under the Cumulative Ranking curve Probability Sorting Plot for **Subclinical baseline severity**

**
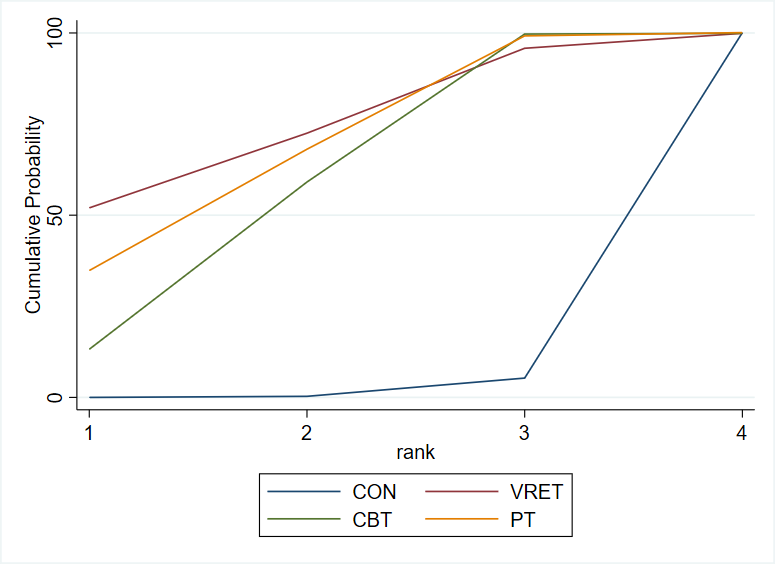
**

**Notes: CON:**Control group **VRET:**Virtual Reality Exposure Therapy **CBT:**Cognitive behavioral therapy **PT:**Psychotherapy

**Supplementary S12.61** – Surface Under the Cumulative Ranking curve Probability Sorting Plot for **Moderate to Severe baseline severity**

**
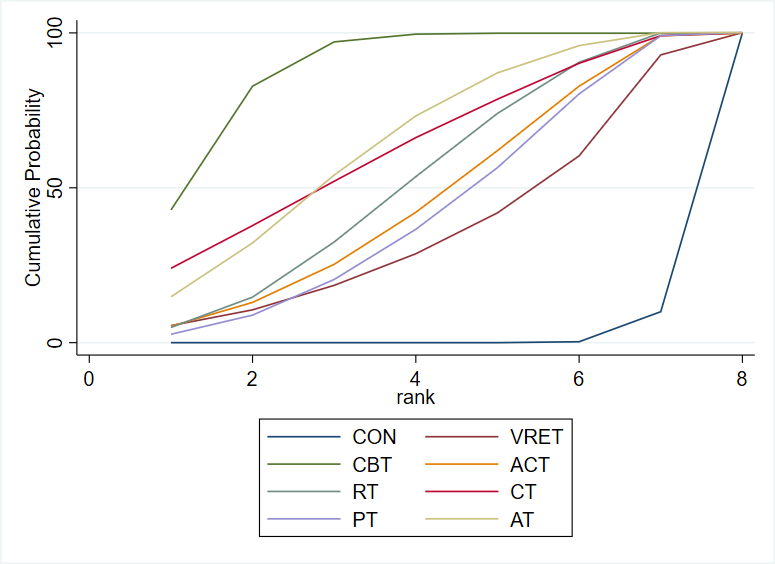
**

**Notes: CON:**Control group **VRET:**Virtual Reality Exposure Therapy **CBT:**Cognitive behavioral therapy **ACT:**Acceptance and Commitment Therapy **RT:**Relaxation therapy **CT**:Combination therapy **PT:**Psychotherapy **AT:**Attention training

**Supplementary S12.62** – Surface Under the Cumulative Ranking curve Probability Sorting Plot for **Severe baseline severity**

**
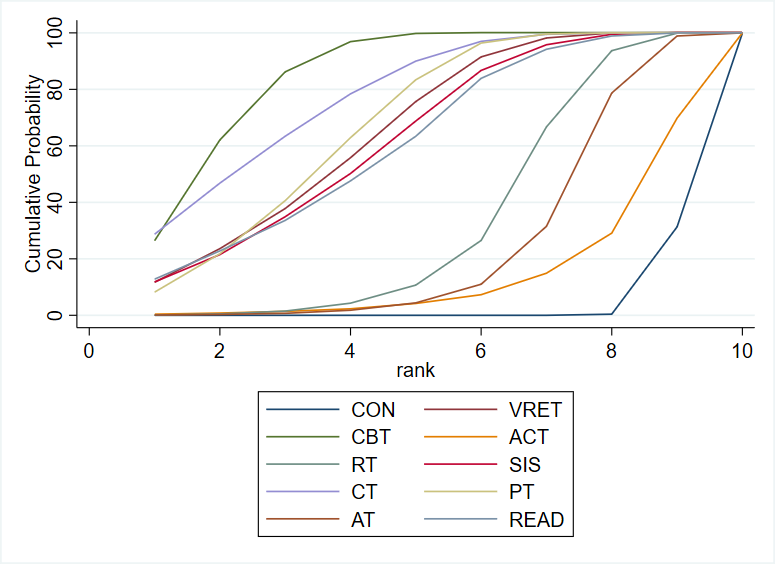
**

**Notes: CON:**Control group **VRET:**Virtual Reality Exposure Therapy **CBT:**Cognitive behavioral therapy **ACT:**Acceptance and Commitment Therapy **RT:**Relaxation therapy **SIS:**Social and Interpersonal Skills **CT**:Combination therapy **PT:**Psychotherapy **AT:**Attention training **READ:**Reading therapy

**Supplementary S12.63**– Surface Under the Cumulative Ranking curve Probability Sorting Plot for **Individual face-to-face format**

**
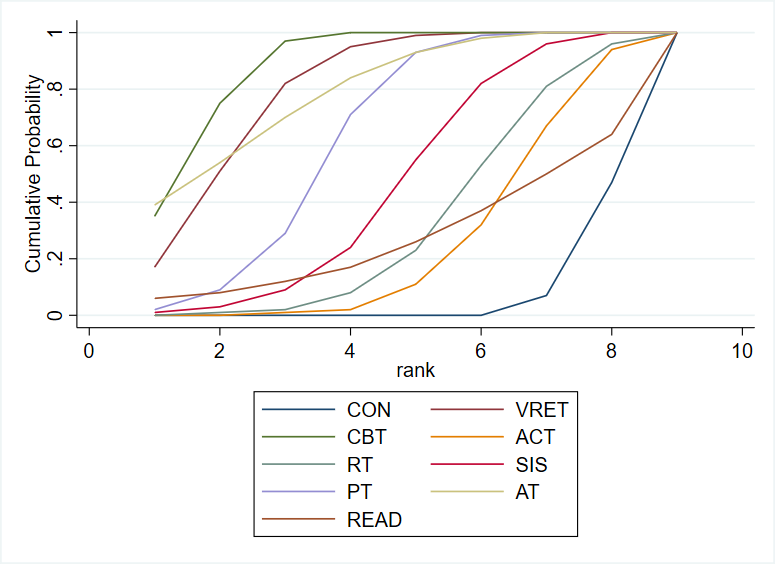
**

**Notes: CON:**Control group **VRET:**Virtual Reality Exposure Therapy **CBT:**Cognitive behavioral therapy **ACT:**Acceptance and Commitment Therapy **RT:**Relaxation therapy **SIS:**Social and Interpersonal Skills **PT:**Psychotherapy **AT:**Attention training **READ:**Reading therapy

**Supplementary S12.64**– Surface Under the Cumulative Ranking curve Probability Sorting Plot for **Group face-to-face format**

**
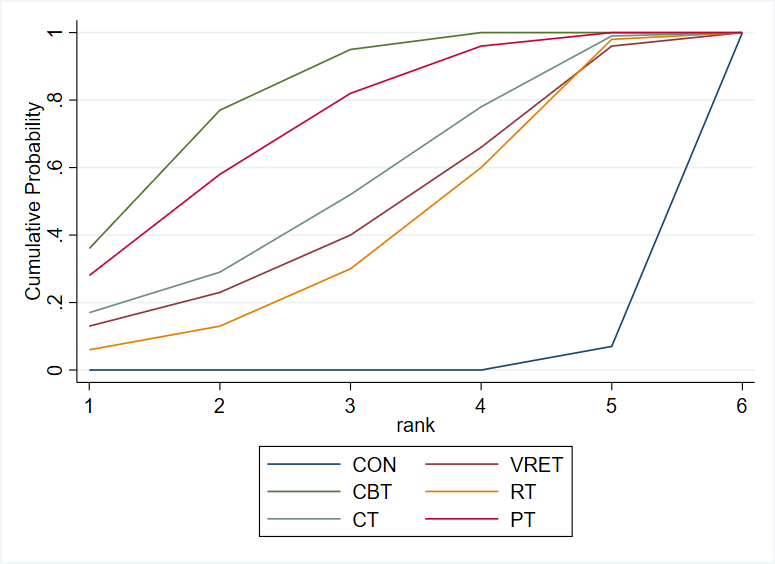
**

**Notes: CON:**Control group **VRET:**Virtual Reality Exposure Therapy **CBT:**Cognitive behavioral therapy **RT:**Relaxation therapy **CT**:Combination therapy **PT:**Psychotherapy**Supplementary S12.65**– Surface Under the Cumulative Ranking curve Probability Sorting Plot for **Online format**

**
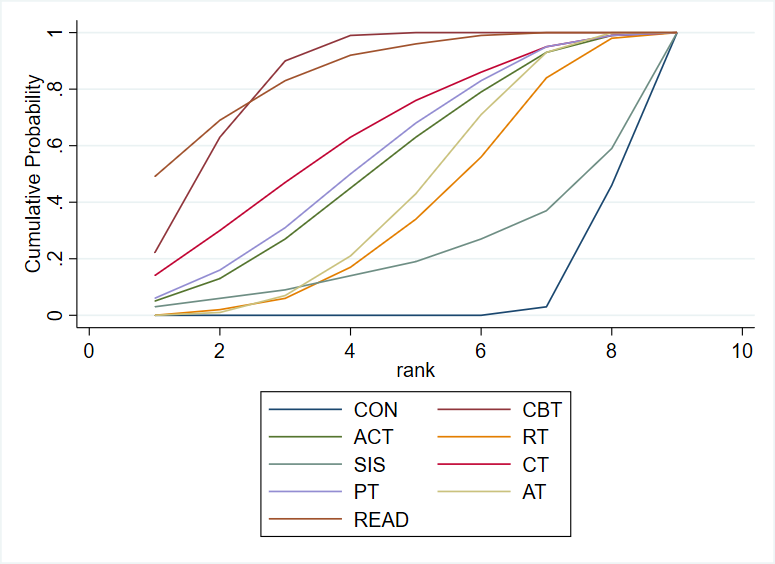
**

**Notes: CON:**Control group **CBT:**Cognitive behavioral therapy **ACT:**Acceptance and Commitment Therapy **RT:**Relaxation therapy **SIS:**Social and Interpersonal Skills **CT**:Combination therapy **PT:**Psychotherapy **AT:**Attention training **READ:**Reading therapy

**Supplementary S12.66**– Surface Under the Cumulative Ranking curve Probability Sorting Plot for **Mixed format**

**
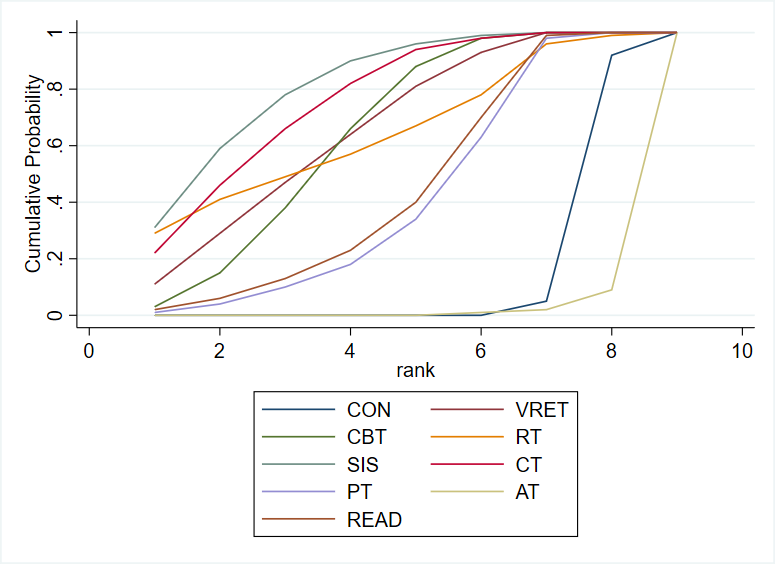
**

**Notes: CON:**Control group **VRET:**Virtual Reality Exposure Therapy **CBT:**Cognitive behavioral therapy **RT:**Relaxation therapy **SIS:**Social and Interpersonal Skills **CT**:Combination therapy **PT:**Psychotherapy **AT:**Attention training **READ:**Reading therapy

**Supplementary S13 –** GRADE assessment

**Supplementary S13.1** – The Grading of Recommendations Assessment, Development and Evaluation (GRADE) assessment for adult social anxiety

| Comparison | Risk of Bias | Publication bias | Indirectness | Imprecision | Inconsistency | Confidence rating |
| --- | --- | --- | --- | --- | --- | --- |
| VRET vs CON | Some concerns | Low risk | Low risk | Low risk | Low risk | Moderate |
| CBT vs CON | Some concerns | Low risk | Low risk | Low risk | Low risk | Moderate |
| ACT vs CON | High risk | Low risk | Low risk | Some concerns | Low risk | Very Low |
| RT vs CON | Some concerns | Low risk | Low risk | Low risk | Low risk | Moderate |
| SIS vs CON | High risk | Low risk | Low risk | Low risk | Low risk | Low |
| CT vs CON | High risk | Low risk | Low risk | Low risk | Low risk | Low |
| PT vs CON | Some concerns | Low risk | Low risk | Low risk | Low risk | Moderate |
| AT vs CON | Some concerns | Low risk | Low risk | Low risk | Low risk | Moderate |
| READ vs CON | Some concerns | Low risk | Low risk | Low risk | Low risk | Moderate |
| CBT vs VRET | Low risk | Low risk | Low risk | Some concerns | Low risk | Moderate |
| ACT vs VRET | Some concerns | Low risk | Low risk | Some concerns | Low risk | Low |
| RT vs VRET | High risk | Low risk | Low risk | Some concerns | Low risk | Very Low |
| SIS vs VRET | High risk | Low risk | Low risk | Some concerns | Low risk | Very Low |
| CT vs VRET | High risk | Low risk | Low risk | Some concerns | Low risk | Very Low |
| PT vs VRET | Low risk | Low risk | Low risk | Some concerns | Low risk | Moderate |
| AT vs VRET | Some concerns | Low risk | Low risk | Some concerns | Low risk | Low |
| READ vs VRET | Some concerns | Low risk | Low risk | Some concerns | Low risk | Low |
| ACT vs CBT | Some concerns | Low risk | Low risk | Low risk | Low risk | Moderate |
| RT vs CBT | Some concerns | Low risk | Low risk | Low risk | Low risk | Moderate |
| SIS vs CBT | Low risk | Low risk | Low risk | Some concerns | Low risk | Moderate |
| CT vs CBT | Low risk | Low risk | Low risk | Some concerns | Low risk | Moderate |
| PT vs CBT | Low risk | Low risk | Low risk | Some concerns | Low risk | Moderate |
| AT vs CBT | Some concerns | Low risk | Low risk | Low risk | Low risk | Moderate |
| READ vs CBT | High risk | Low risk | Low risk | Some concerns | Low risk | Very Low |
| RT vs ACT | Low risk | Low risk | Low risk | Some concerns | Low risk | Moderate |
| SIS vs ACT | Low risk | Low risk | Low risk | Some concerns | Low risk | Moderate |
| CT vs ACT | Low risk | Low risk | Low risk | Some concerns | Low risk | Moderate |
| PT vs ACT | Low risk | Low risk | Low risk | Some concerns | Low risk | Moderate |
| AT vs ACT | Low risk | Low risk | Low risk | Some concerns | Low risk | Moderate |
| READ vs ACT | Some concerns | Low risk | Low risk | Some concerns | Low risk | Low |
| SIS vs RT | Low risk | Low risk | Low risk | Some concerns | Low risk | Moderate |
| CT vs RT | High risk | Low risk | Low risk | Some concerns | Low risk | Very Low |
| PT vs RT | High risk | Low risk | Low risk | Some concerns | Low risk | Very Low |
| AT  vs RT | High risk | Low risk | Low risk | Some concerns | Low risk | Very Low |
| READ vs RT | High risk | Low risk | Low risk | Some concerns | Low risk | Very Low |
| CT vs SIS | Low risk | Low risk | Low risk | Some concerns | Low risk | Moderate |
| PT vs SIS | Some concerns | Low risk | Low risk | Some concerns | Low risk | Low |
| AT  vs SIS | Some concerns | Low risk | Low risk | Some concerns | Low risk | Low |
| READ vs SIS | Some concerns | Low risk | Low risk | Some concerns | Low risk | Low |
| PT  vs CT | High risk | Low risk | Low risk | Some concerns | Low risk | Very Low |
| AT   vs CT | Some concerns | Low risk | Low risk | Some concerns | Low risk | Low |
| READ   vs CT | High risk | Low risk | Low risk | Some concerns | Low risk | Very Low |
| AT vs PT | Some concerns | Low risk | Low risk | Low risk | Low risk | Moderate |
| READ vs PT | Some concerns | Low risk | Low risk | Some concerns | Low risk | Low |
| READ vs AT | Some concerns | Low risk | Low risk | Some concerns | Low risk | Low |

**CON:**Control group **VRET:**Virtual Reality Exposure Therapy **CBT:**Cognitive behavioral therapy **ACT:**Acceptance and Commitment Therapy **RT:**Relaxation therapy **SIS:**Social and Interpersonal Skills **CT**:Combination therapy **PT:**Psychotherapy **AT:**Attention training **READ:**Reading therapy

**Supplementary S13.2** – The Grading of Recommendations Assessment, Development and Evaluation (GRADE) assessment for developed country

| Comparison | Risk of Bias | Publication bias | Indirectness | Imprecision | Inconsistency | Confidence rating |
| --- | --- | --- | --- | --- | --- | --- |
| VRET vs CON | Some concerns | Low risk | Low risk | Low risk | Low risk | Moderate |
| CBT vs CON | Some concerns | Low risk | Low risk | Low risk | Low risk | Moderate |
| ACT vs CON | High risk | Low risk | Low risk | Some concerns | Low risk | Very Low |
| RT vs CON | Some concerns | Low risk | Low risk | Low risk | Low risk | Moderate |
| SIS vs CON | High risk | Low risk | Low risk | Low risk | Low risk | Low |
| CT vs CON | High risk | Low risk | Low risk | Low risk | Low risk | Low |
| PT vs CON | Some concerns | Low risk | Low risk | Low risk | Low risk | Moderate |
| AT vs CON | Some concerns | Low risk | Low risk | Low risk | Low risk | Moderate |
| READ vs CON | Some concerns | Low risk | Low risk | Low risk | Low risk | Moderate |
| CBT vs VRET | Low risk | Low risk | Low risk | Some concerns | Low risk | Moderate |
| ACT vs VRET | Some concerns | Low risk | Low risk | Some concerns | Low risk | Low |
| RT vs VRET | High risk | Low risk | Low risk | Some concerns | Low risk | Very Low |
| SIS vs VRET | High risk | Low risk | Low risk | Some concerns | Low risk | Very Low |
| CT vs VRET | High risk | Low risk | Low risk | Some concerns | Low risk | Very Low |
| PT vs VRET | Low risk | Low risk | Low risk | Some concerns | Low risk | Moderate |
| AT vs VRET | Some concerns | Low risk | Low risk | Low risk | Low risk | Moderate |
| READ vs VRET | Some concerns | Low risk | Low risk | Some concerns | Low risk | Low |
| ACT vs CBT | Some concerns | Low risk | Low risk | Low risk | Low risk | Moderate |
| RT vs CBT | Some concerns | Low risk | Low risk | Low risk | Low risk | Moderate |
| SIS vs CBT | Low risk | Low risk | Low risk | Some concerns | Low risk | Moderate |
| CT vs CBT | Low risk | Low risk | Low risk | Some concerns | Low risk | Moderate |
| PT vs CBT | Low risk | Low risk | Low risk | Some concerns | Low risk | Moderate |
| AT vs CBT | Some concerns | Low risk | Low risk | Low risk | Low risk | Moderate |
| READ vs CBT | High risk | Low risk | Low risk | Some concerns | Low risk | Very Low |
| RT vs ACT | Low risk | Low risk | Low risk | Some concerns | Low risk | Moderate |
| SIS vs ACT | Low risk | Low risk | Low risk | Some concerns | Low risk | Moderate |
| CT vs ACT | Low risk | Low risk | Low risk | Some concerns | Low risk | Moderate |
| PT vs ACT | Low risk | Low risk | Low risk | Some concerns | Low risk | Moderate |
| AT vs ACT | Some concerns | Low risk | Low risk | Some concerns | Low risk | Low |
| READ vs ACT | Some concerns | Low risk | Low risk | Some concerns | Low risk | Low |
| SIS vs RT | Low risk | Low risk | Low risk | Some concerns | Low risk | Moderate |
| CT vs RT | Some concerns | Low risk | Low risk | Some concerns | Low risk | Low |
| PT vs RT | Some concerns | Low risk | Low risk | Some concerns | Low risk | Low |
| AT  vs RT | Some concerns | Low risk | Low risk | Some concerns | Low risk | Low |
| READ vs RT | High risk | Low risk | Low risk | Some concerns | Low risk | Very Low |
| CT vs SIS | Low risk | Low risk | Low risk | Some concerns | Low risk | Moderate |
| PT vs SIS | Low risk | Low risk | Low risk | Some concerns | Low risk | Moderate |
| AT  vs SIS | Some concerns | Low risk | Low risk | Some concerns | Low risk | Low |
| READ vs SIS | Some concerns | Low risk | Low risk | Some concerns | Low risk | Low |
| PT  vs CT | Some concerns | Low risk | Low risk | Some concerns | Low risk | Low |
| AT   vs CT | Some concerns | Low risk | Low risk | Low risk | Low risk | Moderate |
| READ   vs CT | Some concerns | Low risk | Low risk | Some concerns | Low risk | Low |
| AT vs PT | Some concerns | Low risk | Low risk | Low risk | Low risk | Moderate |
| READ vs PT | Some concerns | Low risk | Low risk | Some concerns | Low risk | Low |
| READ vs AT | Some concerns | Low risk | Low risk | Some concerns | Low risk | Low |

**CON:**Control group **VRET:**Virtual Reality Exposure Therapy **CBT:**Cognitive behavioral therapy **ACT:**Acceptance and Commitment Therapy **RT:**Relaxation therapy **SIS:**Social and Interpersonal Skills **CT**:Combination therapy **PT:**Psychotherapy **AT:**Attention training **READ:**Reading therapy

**Supplementary S13.3** – The Grading of Recommendations Assessment, Development and Evaluation (GRADE) assessment for developing country

| Comparison | Risk of Bias | Publication bias | Indirectness | Imprecision | Inconsistency | Confidence rating |
| --- | --- | --- | --- | --- | --- | --- |
| VRET vs CON | High risk | Low risk | Low risk | Low risk | Low risk | Low |
| CBT vs CON | High risk | Low risk | Low risk | Low risk | Low risk | Low |
| PT vs CON | Some concerns | Low risk | Low risk | Low risk | Low risk | Moderate |
| AT vs CON | High risk | Low risk | Low risk | Some concerns | Low risk | Very Low |
| CBT vs VRET | Some concerns | Low risk | Low risk | Some concerns | Low risk | Low |
| PT vs VRET | Some concerns | Low risk | Low risk | Some concerns | Low risk | Low |
| AT vs VRET | High risk | Low risk | Low risk | Some concerns | Low risk | Very Low |
| PT vs CBT | Low risk | Low risk | Low risk | Some concerns | Low risk | Moderate |
| AT vs CBT | High risk | Low risk | Low risk | Some concerns | Low risk | Very Low |
| AT vs PT | Some concerns | Low risk | Low risk | Some concerns | Low risk | Low |

**Notes: CON:**Control group **VRET:**Virtual Reality Exposure Therapy **CBT:**Cognitive behavioral therapy **PT:**Psychotherapy **AT:**Attention training

**Supplementary S13.4** – The Grading of Recommendations Assessment, Development and Evaluation (GRADE) assessment for Intervention duration of ≥ 8 weeks

| Comparison | Risk of Bias | Publication bias | Indirectness | Imprecision | Inconsistency | Confidence rating |
| --- | --- | --- | --- | --- | --- | --- |
| VRET vs CON | High risk | Low risk | Low risk | Low risk | Low risk | Very Low |
| CBT vs CON | Some concerns | Low risk | Low risk | Low risk | Low risk | Moderate |
| ACT vs CON | High risk | Low risk | Low risk | Some concerns | Low risk | Very Low |
| RT vs CON | Some concerns | Low risk | Low risk | Low risk | Low risk | Moderate |
| SIS vs CON | High risk | Low risk | Low risk | Low risk | Low risk | Low |
| CT vs CON | High risk | Low risk | Low risk | Low risk | Low risk | Low |
| PT vs CON | Some concerns | Low risk | Low risk | Low risk | Low risk | Moderate |
| AT vs CON | High risk | Low risk | Low risk | Low risk | Low risk | Low |
| READ vs CON | Some concerns | Low risk | Low risk | Low risk | Low risk | Moderate |
| CBT vs VRET | Low risk | Low risk | Low risk | Some concerns | Low risk | Moderate |
| ACT vs VRET | Some concerns | Low risk | Low risk | Low risk | Low risk | Moderate |
| RT vs VRET | High risk | Low risk | Low risk | Some concerns | Low risk | Very Low |
| SIS vs VRET | High risk | Low risk | Low risk | Some concerns | Low risk | Very Low |
| CT vs VRET | Some concerns | Low risk | Low risk | Some concerns | Low risk | Low |
| PT vs VRET | Some concerns | Low risk | Low risk | Some concerns | Low risk | Low |
| AT vs VRET | High risk | Low risk | Low risk | Some concerns | Low risk | Very Low |
| READ vs VRET | Some concerns | Low risk | Low risk | Some concerns | Low risk | Low |
| ACT vs CBT | Some concerns | Low risk | Low risk | Low risk | Low risk | Moderate |
| RT vs CBT | Some concerns | Low risk | Low risk | Low risk | Low risk | Moderate |
| SIS vs CBT | Low risk | Low risk | Low risk | Some concerns | Low risk | Moderate |
| CT vs CBT | Low risk | Low risk | Low risk | Some concerns | Low risk | Moderate |
| PT vs CBT | Low risk | Low risk | Low risk | Some concerns | Low risk | Moderate |
| AT vs CBT | Some concerns | Low risk | Low risk | Some concerns | Low risk | Low |
| READ vs CBT | High risk | Low risk | Low risk | Some concerns | Low risk | Very Low |
| RT vs ACT | Low risk | Low risk | Low risk | Some concerns | Low risk | Moderate |
| SIS vs ACT | Some concerns | Low risk | Low risk | Low risk | Low risk | Moderate |
| CT vs ACT | Some concerns | Low risk | Low risk | Low risk | Low risk | Moderate |
| PT vs ACT | Some concerns | Low risk | Low risk | Low risk | Low risk | Moderate |
| AT vs ACT | Some concerns | Low risk | Low risk | Low risk | Low risk | Moderate |
| READ vs ACT | Some concerns | Low risk | Low risk | Low risk | Low risk | Moderate |
| SIS vs RT | Low risk | Low risk | Low risk | Some concerns | Low risk | Moderate |
| CT vs RT | Some concerns | Low risk | Low risk | Some concerns | Low risk | Low |
| PT vs RT | Some concerns | Low risk | Low risk | Some concerns | Low risk | Low |
| AT  vs RT | Some concerns | Low risk | Low risk | Some concerns | Low risk | Low |
| READ vs RT | Some concerns | Low risk | Low risk | Some concerns | Low risk | Low |
| CT vs SIS | Low risk | Low risk | Low risk | Some concerns | Low risk | Moderate |
| PT vs SIS | Some concerns | Low risk | Low risk | Some concerns | Low risk | Low |
| AT  vs SIS | High risk | Low risk | Low risk | Some concerns | Low risk | Very Low |
| READ vs SIS | Some concerns | Low risk | Low risk | Some concerns | Low risk | Low |
| PT  vs CT | Some concerns | Low risk | Low risk | Some concerns | Low risk | Low |
| AT   vs CT | High risk | Low risk | Low risk | Some concerns | Low risk | Very Low |
| READ   vs CT | Some concerns | Low risk | Low risk | Some concerns | Low risk | Low |
| AT vs PT | Some concerns | Low risk | Low risk | Some concerns | Low risk | Low |
| READ vs PT | Some concerns | Low risk | Low risk | Some concerns | Low risk | Low |
| READ vs AT | Some concerns | Low risk | Low risk | Some concerns | Low risk | Low |

**CON:**Control group **VRET:**Virtual Reality Exposure Therapy **CBT:**Cognitive behavioral therapy **ACT:**Acceptance and Commitment Therapy **RT:**Relaxation therapy **SIS:**Social and Interpersonal Skills **CT**:Combination therapy **PT:**Psychotherapy **AT:**Attention training **READ:**Reading therapy

**Supplementary S13.5** – The Grading of Recommendations Assessment, Development and Evaluation (GRADE) assessment for Intervention duration of ＜ 8 weeks

| Comparison | Risk of Bias | Publication bias | Indirectness | Imprecision | Inconsistency | Confidence rating |
| --- | --- | --- | --- | --- | --- | --- |
| VRET vs CON | Some concerns | Low risk | Low risk | Low risk | Low risk | Moderate |
| CBT vs CON | Some concerns | Low risk | Low risk | Low risk | Low risk | Moderate |
| ACT vs CON | High risk | Low risk | Low risk | Low risk | Low risk | Low |
| RT vs CON | High risk | Low risk | Low risk | Some concerns | Low risk | Very Low |
| CT vs CON | High risk | Low risk | Low risk | Some concerns | Low risk | Very Low |
| PT vs CON | Some concerns | Low risk | Low risk | Low risk | Low risk | Moderate |
| AT vs CON | Some concerns | Low risk | Low risk | Low risk | Low risk | Moderate |
| READ vs CON | Some concerns | Low risk | Low risk | Some concerns | Low risk | Low |
| CBT vs VRET | High risk | Low risk | Low risk | Some concerns | Low risk | Very Low |
| ACT vs VRET | Some concerns | Low risk | Low risk | Some concerns | Low risk | Moderate |
| RT vs VRET | Some concerns | Low risk | Low risk | Some concerns | Low risk | Low |
| CT vs VRET | High risk | Low risk | Low risk | Some concerns | Low risk | Very Low |
| PT vs VRET | Some concerns | Low risk | Low risk | Some concerns | Low risk | Low |
| AT vs VRET | Some concerns | Low risk | Low risk | Some concerns | Low risk | Low |
| READ vs VRET | Some concerns | Low risk | Low risk | Some concerns | Low risk | Low |
| ACT vs CBT | Low risk | Low risk | Low risk | Some concerns | Low risk | Moderate |
| RT vs CBT | Low risk | Low risk | Low risk | Some concerns | Low risk | Moderate |
| CT vs CBT | High risk | Low risk | Low risk | Some concerns | Low risk | Very Low |
| PT vs CBT | Low risk | Low risk | Low risk | Some concerns | Low risk | Moderate |
| AT vs CBT | Some concerns | Low risk | Low risk | Low risk | Low risk | Moderate |
| READ vs CBT | Some concerns | Low risk | Low risk | Some concerns | Low risk | Low |
| RT vs ACT | Low risk | Low risk | Low risk | Some concerns | Low risk | Moderate |
| CT vs ACT | Some concerns | Low risk | Low risk | Some concerns | Low risk | Low |
| PT vs ACT | Low risk | Low risk | Low risk | Some concerns | Low risk | Moderate |
| AT vs ACT | Some concerns | Low risk | Low risk | Some concerns | Low risk | Low |
| READ vs ACT | Some concerns | Low risk | Low risk | Some concerns | Low risk | Low |
| CT vs RT | Some concerns | Low risk | Low risk | Some concerns | Low risk | Low |
| PT vs RT | Some concerns | Low risk | Low risk | Some concerns | Low risk | Low |
| AT vs RT | Some concerns | Low risk | Low risk | Some concerns | Low risk | Low |
| READ vs RT | High risk | Low risk | Low risk | Some concerns | Low risk | Very Low |
| PT vs CT | Some concerns | Low risk | Low risk | Some concerns | Low risk | Low |
| AT vs CT | Some concerns | Low risk | Low risk | Some concerns | Low risk | Low |
| READ vs CT | Some concerns | Low risk | Low risk | Some concerns | Low risk | Low |
| AT vs PT | Some concerns | Low risk | Low risk | Some concerns | Low risk | Low |
| READ vs PT | High risk | Low risk | Low risk | Some concerns | Low risk | Very Low |
| READ vs AT | Some concerns | Low risk | Low risk | Some concerns | Low risk | Low |

**CON:**Control group **VRET:**Virtual Reality Exposure Therapy **CBT:**Cognitive behavioral therapy **ACT:**Acceptance and Commitment Therapy **RT:**Relaxation therapy **CT**:Combination therapy **PT:**Psychotherapy **AT:**Attention training **READ:**Reading therapy

**Supplementary S13.6** – The Grading of Recommendations Assessment, Development and Evaluation (GRADE) assessment for Subclinical baseline severity

| Comparison | Risk of Bias | Publication bias | Indirectness | Imprecision | Inconsistency | Confidence rating |
| --- | --- | --- | --- | --- | --- | --- |
| VRET vs CON | Some concerns | Low risk | Low risk | Some concerns | Low risk | Low |
| CBT vs CON | Some concerns | Low risk | Low risk | Low risk | Low risk | Moderate |
| PT vs CON | Some concerns | Low risk | Low risk | Low risk | Low risk | Moderate |
| CBT vs VRET | Some concerns | Low risk | Low risk | Some concerns | Low risk | Low |
| PT vs VRET | Some concerns | Low risk | Low risk | Some concerns | Low risk | Low |
| PT vs CBT | Some concerns | Low risk | Low risk | Some concerns | Low risk | Low |

**Notes: CON:**Control group **VRET:**Virtual Reality Exposure Therapy **CBT:**Cognitive behavioral therapy **PT:**Psychotherapy

**Supplementary S13.7** – The Grading of Recommendations Assessment, Development and Evaluation (GRADE) assessment for Moderate to Severe baseline severity

| Comparison | Risk of Bias | Publication bias | Indirectness | Imprecision | Inconsistency | Confidence rating |
| --- | --- | --- | --- | --- | --- | --- |
| VRET vs CON | Low risk | Low risk | Low risk | Some concerns | Low risk | Moderate |
| CBT vs CON | Low risk | Low risk | Low risk | Low risk | Low risk | High |
| ACT vs CON | Low risk | Low risk | Low risk | Low risk | Low risk | High |
| RT vs CON | Low risk | Low risk | Low risk | Low risk | Low risk | High |
| CT vs CON | Low risk | Low risk | Low risk | Low risk | Low risk | High |
| PT vs CON | Low risk | Low risk | Low risk | Low risk | Low risk | High |
| AT vs CON | Low risk | Low risk | Low risk | Low risk | Low risk | High |
| CBT vs VRET | High risk | Low risk | Low risk | Some concerns | Low risk | Very Low |
| ACT vs VRET | Some concerns | Low risk | Low risk | Some concerns | Low risk | Low |
| RT vs VRET | Some concerns | Low risk | Low risk | Some concerns | Low risk | Low |
| CT vs VRET | Some concerns | Low risk | Low risk | Some concerns | Low risk | Low |
| PT vs VRET | Some concerns | Low risk | Low risk | Some concerns | Low risk | Low |
| AT vs VRET | Low risk | Low risk | Low risk | Some concerns | Low risk | Moderate |
| ACT vs CBT | Low risk | Low risk | Low risk | Some concerns | Low risk | Moderate |
| RT vs CBT | Low risk | Low risk | Low risk | Some concerns | Low risk | Moderate |
| CT vs CBT | Low risk | Low risk | Low risk | Some concerns | Low risk | Moderate |
| PT vs CBT | Low risk | Low risk | Low risk | Some concerns | Low risk | Moderate |
| AT vs CBT | Some concerns | Low risk | Low risk | Some concerns | Low risk | Low |
| RT vs ACT | Low risk | Low risk | Low risk | Some concerns | Low risk | Moderate |
| CT vs ACT | Low risk | Low risk | Low risk | Some concerns | Low risk | Moderate |
| PT vs ACT | Low risk | Low risk | Low risk | Some concerns | Low risk | Moderate |
| AT vs ACT | Some concerns | Low risk | Low risk | Some concerns | Low risk | Low |
| CT vs RT | Low risk | Low risk | Low risk | Some concerns | Low risk | Moderate |
| PT vs RT | Low risk | Low risk | Low risk | Some concerns | Low risk | Moderate |
| AT vs RT | Some concerns | Low risk | Low risk | Some concerns | Low risk | Low |
| PT vs CT | Low risk | Low risk | Low risk | Some concerns | Low risk | Moderate |
| AT vs CT | Some concerns | Low risk | Low risk | Some concerns | Low risk | Low |
| AT vs PT | Some concerns | Low risk | Low risk | Some concerns | Low risk | Low |

**Notes: CON:**Control group **VRET:**Virtual Reality Exposure Therapy **CBT:**Cognitive behavioral therapy **ACT:**Acceptance and Commitment Therapy **RT:**Relaxation therapy **CT**:Combination therapy **PT:**Psychotherapy **AT:**Attention training

**Supplementary S13.8** – The Grading of Recommendations Assessment, Development and Evaluation (GRADE) assessment for Severe baseline severity

| Comparison | Risk of Bias | Publication bias | Indirectness | Imprecision | Inconsistency | Confidence rating |
| --- | --- | --- | --- | --- | --- | --- |
| VRET vs CON | Low risk | Low risk | Low risk | Low risk | Low risk | High |
| CBT vs CON | Low risk | Low risk | Low risk | Low risk | Low risk | High |
| ACT vs CON | Some concerns | Low risk | Low risk | Some concerns | Low risk | Low |
| RT vs CON | Low risk | Low risk | Low risk | Low risk | Low risk | High |
| SIS vs CON | Low risk | Low risk | Low risk | Low risk | Low risk | High |
| CT vs CON | Low risk | Low risk | Low risk | Low risk | Low risk | High |
| PT vs CON | Low risk | Low risk | Low risk | Low risk | Low risk | High |
| AT vs CON | Some concerns | Low risk | Low risk | Low risk | Low risk | Moderate |
| READ vs CON | Low risk | Low risk | Low risk | Low risk | Low risk | High |
| CBT vs VRET | Low risk | Low risk | Low risk | Some concerns | Low risk | Moderate |
| ACT vs VRET | Low risk | Low risk | Low risk | Some concerns | Low risk | Moderate |
| RT vs VRET | Low risk | Low risk | Low risk | Some concerns | Low risk | Moderate |
| SIS vs VRET | Low risk | Low risk | Low risk | Some concerns | Low risk | Moderate |
| CT vs VRET | High risk | Low risk | Low risk | Some concerns | Low risk | Very Low |
| PT vs VRET | Low risk | Low risk | Low risk | Some concerns | Low risk | Moderate |
| AT vs VRET | Some concerns | Low risk | Low risk | Some concerns | Low risk | Low |
| READ vs VRET | Some concerns | Low risk | Low risk | Some concerns | Low risk | Low |
| ACT vs CBT | Low risk | Low risk | Low risk | Low risk | Low risk | High |
| RT vs CBT | Low risk | Low risk | Low risk | Low risk | Low risk | High |
| SIS vs CBT | Low risk | Low risk | Low risk | Some concerns | Low risk | Moderate |
| CT vs CBT | Low risk | Low risk | Low risk | Some concerns | Low risk | Moderate |
| PT vs CBT | Low risk | Low risk | Low risk | Some concerns | Low risk | Moderate |
| AT vs CBT | High risk | Low risk | Low risk | Low risk | Low risk | Moderate |
| READ vs CBT | Some concerns | Low risk | Low risk | Some concerns | Low risk | Low |
| RT vs ACT | Low risk | Low risk | Low risk | Some concerns | Low risk | Moderate |
| SIS vs ACT | Low risk | Low risk | Low risk | Some concerns | Low risk | Moderate |
| CT vs ACT | Low risk | Low risk | Low risk | Some concerns | Low risk | Moderate |
| PT vs ACT | Low risk | Low risk | Low risk | Some concerns | Low risk | Moderate |
| AT vs ACT | Some concerns | Low risk | Low risk | Some concerns | Low risk | Low |
| READ vs ACT | Some concerns | Low risk | Low risk | Some concerns | Low risk | Low |
| SIS vs RT | Low risk | Low risk | Low risk | Some concerns | Low risk | Moderate |
| CT vs RT | Some concerns | Low risk | Low risk | Some concerns | Low risk | Low |
| PT vs RT | Low risk | Low risk | Low risk | Some concerns | Low risk | Moderate |
| AT vs RT | Some concerns | Low risk | Low risk | Some concerns | Low risk | Low |
| READ vs RT | Some concerns | Low risk | Low risk | Some concerns | Low risk | Low |
| CT vs SIS | Low risk | Low risk | Low risk | Some concerns | Low risk | Moderate |
| PT vs SIS | Low risk | Low risk | Low risk | Some concerns | Low risk | Moderate |
| AT vs SIS | Some concerns | Low risk | Low risk | Some concerns | Low risk | Low |
| READ vs SIS | Some concerns | Low risk | Low risk | Some concerns | Low risk | Low |
| PT vs CT | Low risk | Low risk | Low risk | Some concerns | Low risk | Moderate |
| AT vs CT | Some concerns | Low risk | Low risk | Some concerns | Low risk | Low |
| READ vs CT | Low risk | Low risk | Low risk | Some concerns | Low risk | Moderate |
| AT vs PT | Some concerns | Low risk | Low risk | Low risk | Low risk | Moderate |
| READ vs PT | Low risk | Low risk | Low risk | Some concerns | Low risk | Moderate |
| READ vs AT | Some concerns | Low risk | Low risk | Some concerns | Low risk | Low |

**Notes: CON:**Control group **VRET:**Virtual Reality Exposure Therapy **CBT:**Cognitive behavioral therapy **ACT:**Acceptance and Commitment Therapy **RT:**Relaxation therapy **SIS:**Social and Interpersonal Skills **CT**:Combination therapy **PT:**Psychotherapy **AT:**Attention training **READ:**Reading therapy

**Supplementary S13.9** – The Grading of Recommendations Assessment, Development and Evaluation (GRADE) assessment for Individual face-to-face format

| Comparison | Risk of Bias | Publication bias | Indirectness | Imprecision | Inconsistency | Confidence rating |
| --- | --- | --- | --- | --- | --- | --- |
| VRET vs CON | Some concerns | Low risk | Low risk | Low risk | Low risk | Moderate |
| CBT vs CON | Low risk | Low risk | Low risk | Low risk | Low risk | High |
| ACT vs CON | Some concerns | Low risk | Low risk | Some concerns | Low risk | Low |
| RT vs CON | High risk | Low risk | Low risk | Some concerns | Low risk | Very Low |
| SIS vs CON | Some concerns | Low risk | Low risk | Low risk | Low risk | Moderate |
| PT vs CON | Low risk | Low risk | Low risk | Low risk | Low risk | High |
| AT vs CON | Some concerns | Low risk | Low risk | Low risk | Low risk | Moderate |
| READ vs CON | Some concerns | Low risk | Low risk | Some concerns | Low risk | Low |
| CBT vs VRET | Low risk | Low risk | Low risk | Some concerns | Low risk | Moderate |
| ACT vs VRET | Low risk | Low risk | Low risk | Low risk | Low risk | High |
| RT vs VRET | Low risk | Low risk | Low risk | Low risk | Low risk | High |
| SIS vs VRET | Some concerns | Low risk | Low risk | Some concerns | Low risk | Low |
| PT vs VRET | Low risk | Low risk | Low risk | Some concerns | Low risk | Moderate |
| AT vs VRET | Some concerns | Low risk | Low risk | Some concerns | Low risk | Low |
| READ vs VRET | Some concerns | Low risk | Low risk | Some concerns | Low risk | Low |
| ACT vs CBT | Low risk | Low risk | Low risk | Low risk | Low risk | High |
| RT vs CBT | Low risk | Low risk | Low risk | Low risk | Low risk | High |
| SIS vs CBT | High risk | Low risk | Low risk | Low risk | Low risk | Low |
| PT vs CBT | Low risk | Low risk | Low risk | Some concerns | Low risk | Moderate |
| AT vs CBT | Some concerns | Low risk | Low risk | Some concerns | Low risk | Low |
| READ vs CBT | Some concerns | Low risk | Low risk | Some concerns | Low risk | Low |
| RT vs ACT | Low risk | Low risk | Low risk | Some concerns | Low risk | Moderate |
| SIS vs ACT | Some concerns | Low risk | Low risk | Some concerns | Low risk | Low |
| PT vs ACT | Low risk | Low risk | Low risk | Some concerns | Low risk | Moderate |
| AT vs ACT | Some concerns | Low risk | Low risk | Some concerns | Low risk | Low |
| READ vs ACT | Some concerns | Low risk | Low risk | Some concerns | Low risk | Low |
| SIS vs RT | Some concerns | Low risk | Low risk | Some concerns | Low risk | Low |
| PT vs RT | Low risk | Low risk | Low risk | Some concerns | Low risk | Moderate |
| AT vs RT | Some concerns | Low risk | Low risk | Some concerns | Low risk | Low |
| READ vs RT | Some concerns | Low risk | Low risk | Some concerns | Low risk | Low |
| PT vs SIS | Some concerns | Low risk | Low risk | Some concerns | Low risk | Low |
| AT vs SIS | Some concerns | Low risk | Low risk | Some concerns | Low risk | Low |
| READ vs SIS | Some concerns | Low risk | Low risk | Some concerns | Low risk | Low |
| AT vs PT | Some concerns | Low risk | Low risk | Some concerns | Low risk | Low |
| READ vs PT | Some concerns | Low risk | Low risk | Some concerns | Low risk | Low |
| READ vs AT | Some concerns | Low risk | Low risk | Some concerns | Low risk | Low |

**Notes: CON:**Control group **VRET:**Virtual Reality Exposure Therapy **CBT:**Cognitive behavioral therapy **ACT:**Acceptance and Commitment Therapy **RT:**Relaxation therapy **SIS:**Social and Interpersonal Skills **PT:**Psychotherapy **AT:**Attention training **READ:**Reading therapy

**Supplementary S13.10** – The Grading of Recommendations Assessment, Development and Evaluation (GRADE) assessment for Group face-to-face format

| Comparison | Risk of Bias | Publication bias | Indirectness | Imprecision | Inconsistency | Confidence rating |
| --- | --- | --- | --- | --- | --- | --- |
| VRET vs CON | High risk | Low risk | Low risk | Some concerns | Low risk | Very Low |
| CBT vs CON | Low risk | Low risk | Low risk | Low risk | Low risk | High |
| RT vs CON | Low risk | Low risk | Low risk | Some concerns | Low risk | Moderate |
| CT vs CON | Some concerns | Low risk | Low risk | Low risk | Low risk | Moderate |
| PT vs CON | Low risk | Low risk | Low risk | Low risk | Low risk | High |
| CBT vs VRET | Some concerns | Low risk | Low risk | Some concerns | Low risk | Low |
| RT vs VRET | Some concerns | Low risk | Low risk | Some concerns | Low risk | Low |
| CT vs VRET | High risk | Low risk | Low risk | Some concerns | Low risk | Very Low |
| PT vs VRET | Some concerns | Low risk | Low risk | Some concerns | Low risk | Low |
| RT vs CBT | Low risk | Low risk | Low risk | Some concerns | Low risk | Moderate |
| CT vs CBT | Low risk | Low risk | Low risk | Some concerns | Low risk | Moderate |
| PT vs CBT | Low risk | Low risk | Low risk | Some concerns | Low risk | Moderate |
| CT vs RT | Low risk | Low risk | Low risk | Some concerns | Low risk | Moderate |
| PT vs RT | Low risk | Low risk | Low risk | Some concerns | Low risk | Moderate |
| PT vs CT | Some concerns | Low risk | Low risk | Some concerns | Low risk | Low |

**Notes: CON:**Control group **VRET:**Virtual Reality Exposure Therapy **CBT:**Cognitive behavioral therapy **RT:**Relaxation therapy **CT**:Combination therapy **PT:**Psychotherapy

**Supplementary S13.11** – The Grading of Recommendations Assessment, Development and Evaluation (GRADE) assessment for Online format

| Comparison | Risk of Bias | Publication bias | Indirectness | Imprecision | Inconsistency | Confidence rating |
| --- | --- | --- | --- | --- | --- | --- |
| CBT vs CON | Low risk | Low risk | Low risk | Low risk | Low risk | High |
| ACT vs CON | Low risk | Low risk | Low risk | Low risk | Low risk | High |
| RT vs CON | Low risk | Low risk | Low risk | Some concerns | Low risk | Moderate |
| SIS vs CON | Low risk | Low risk | Low risk | Some concerns | Low risk | Moderate |
| CT vs CON | Some concerns | Low risk | Low risk | Low risk | Low risk | Moderate |
| PT vs CON | Low risk | Low risk | Low risk | Low risk | Low risk | High |
| AT vs CON | Low risk | Low risk | Low risk | Low risk | Low risk | High |
| READ vs CON | Some concerns | Low risk | Low risk | Low risk | Low risk | Moderate |
| ACT vs CBT | Low risk | Low risk | Low risk | Some concerns | Low risk | Moderate |
| RT vs CBT | Some concerns | Low risk | Low risk | Low risk | Low risk | Moderate |
| SIS vs CBT | Low risk | Low risk | Low risk | Some concerns | Low risk | Moderate |
| CT vs CBT | Some concerns | Low risk | Low risk | Some concerns | Low risk | Low |
| PT vs CBT | Low risk | Low risk | Low risk | Some concerns | Low risk | Moderate |
| AT vs CBT | Some concerns | Low risk | Low risk | Low risk | Low risk | Moderate |
| READ vs CBT | Some concerns | Low risk | Low risk | Some concerns | Low risk | Low |
| RT vs ACT | Some concerns | Low risk | Low risk | Some concerns | Low risk | Low |
| SIS vs ACT | Low risk | Low risk | Low risk | Some concerns | Low risk | Moderate |
| CT vs ACT | Some concerns | Low risk | Low risk | Some concerns | Low risk | Low |
| PT vs ACT | Low risk | Low risk | Low risk | Some concerns | Low risk | Moderate |
| AT vs ACT | Some concerns | Low risk | Low risk | Some concerns | Low risk | Low |
| READ vs ACT | Some concerns | Low risk | Low risk | Some concerns | Low risk | Low |
| SIS vs RT | Some concerns | Low risk | Low risk | Some concerns | Low risk | Low |
| CT vs RT | Some concerns | Low risk | Low risk | Some concerns | Low risk | Low |
| PT vs RT | Low risk | Low risk | Low risk | Some concerns | Low risk | Moderate |
| AT vs RT | Some concerns | Low risk | Low risk | Some concerns | Low risk | Low |
| READ vs RT | Some concerns | Low risk | Low risk | Some concerns | Low risk | Low |
| CT vs SIS | Some concerns | Low risk | Low risk | Some concerns | Low risk | Low |
| PT vs SIS | Some concerns | Low risk | Low risk | Some concerns | Low risk | Low |
| AT vs SIS | Some concerns | Low risk | Low risk | Some concerns | Low risk | Low |
| READ vs SIS | Some concerns | Low risk | Low risk | Some concerns | Low risk | Low |
| PT vs CT | Some concerns | Low risk | Low risk | Some concerns | Low risk | Low |
| AT vs CT | Some concerns | Low risk | Low risk | Some concerns | Low risk | Low |
| READ vs CT | Some concerns | Low risk | Low risk | Some concerns | Low risk | Low |
| AT vs PT | Some concerns | Low risk | Low risk | Some concerns | Low risk | Low |
| READ vs PT | Some concerns | Low risk | Low risk | Some concerns | Low risk | Low |
| READ vs AT | Some concerns | Low risk | Low risk | Some concerns | Low risk | Low |

**Notes: CON:**Control group **CBT:**Cognitive behavioral therapy **ACT:**Acceptance and Commitment Therapy **RT:**Relaxation therapy **SIS:**Social and Interpersonal Skills **CT**:Combination therapy **PT:**Psychotherapy **AT:**Attention training **READ:**Reading therapy

**Supplementary S13.12** – The Grading of Recommendations Assessment, Development and Evaluation (GRADE) assessment for Mixed foemat

| Comparison | Risk of Bias | Publication bias | Indirectness | Imprecision | Inconsistency | Confidence rating |
| --- | --- | --- | --- | --- | --- | --- |
| VRET vs CON | Low risk | Low risk | Low risk | Low risk | Low risk | High |
| CBT vs CON | Low risk | Low risk | Low risk | Low risk | Low risk | High |
| RT vs CON | Low risk | Low risk | Low risk | Some concerns | Low risk | Moderate |
| SIS vs CON | Low risk | Low risk | Low risk | Low risk | Low risk | High |
| CT vs CON | Low risk | Low risk | Low risk | Low risk | Low risk | High |
| PT vs CON | Low risk | Low risk | Low risk | Low risk | Low risk | High |
| AT vs CON | High risk | Low risk | Low risk | Some concerns | Low risk | Very Low |
| READ vs CON | Low risk | Low risk | Low risk | Low risk | Low risk | High |
| CBT vs VRET | Low risk | Low risk | Low risk | Some concerns | Low risk | Moderate |
| RT vs VRET | Low risk | Low risk | Low risk | Some concerns | Low risk | Moderate |
| SIS vs VRET | Low risk | Low risk | Low risk | Some concerns | Low risk | Moderate |
| CT vs VRET | Some concerns | Low risk | Low risk | Some concerns | Low risk | Low |
| PT vs VRET | Low risk | Low risk | Low risk | Some concerns | Low risk | Moderate |
| AT vs VRET | Some concerns | Low risk | Low risk | Low risk | Low risk | Moderate |
| READ vs VRET | Low risk | Low risk | Low risk | Some concerns | Low risk | Moderate |
| RT vs CBT | Low risk | Low risk | Low risk | Some concerns | Low risk | Moderate |
| SIS vs CBT | Low risk | Low risk | Low risk | Some concerns | Low risk | Moderate |
| CT vs CBT | Low risk | Low risk | Low risk | Some concerns | Low risk | Moderate |
| PT vs CBT | Some concerns | Low risk | Low risk | Some concerns | Low risk | Low |
| AT vs CBT | Some concerns | Low risk | Low risk | Low risk | Low risk | Moderate |
| READ vs CBT | Low risk | Low risk | Low risk | Some concerns | Low risk | Moderate |
| SIS vs RT | Low risk | Low risk | Low risk | Some concerns | Low risk | Moderate |
| CT vs RT | Low risk | Low risk | Low risk | Some concerns | Low risk | Moderate |
| PT vs RT | Some concerns | Low risk | Low risk | Some concerns | Low risk | Low |
| AT vs RT | Some concerns | Low risk | Low risk | Some concerns | Low risk | Low |
| READ vs RT | Low risk | Low risk | Low risk | Some concerns | Low risk | Moderate |
| CT vs SIS | Low risk | Low risk | Low risk | Some concerns | Low risk | Moderate |
| PT vs SIS | Some concerns | Low risk | Low risk | Some concerns | Low risk | Low |
| AT vs SIS | Some concerns | Low risk | Low risk | Low risk | Low risk | Moderate |
| READ vs SIS | Low risk | Low risk | Low risk | Some concerns | Low risk | Moderate |
| PT vs CT | Low risk | Low risk | Low risk | Some concerns | Low risk | Moderate |
| AT vs CT | Some concerns | Low risk | Low risk | Low risk | Low risk | Moderate |
| READ vs CT | Low risk | Low risk | Low risk | Some concerns | Low risk | Moderate |
| AT vs PT | Some concerns | Low risk | Low risk | Low risk | Low risk | Moderate |
| READ vs PT | Low risk | Low risk | Low risk | Some concerns | Low risk | Moderate |
| READ vs AT | Some concerns | Low risk | Low risk | Some concerns | Low risk | Low |

**Notes: CON:**Control group **VRET:**Virtual Reality Exposure Therapy **CBT:**Cognitive behavioral therapy **RT:**Relaxation therapy **SIS:**Social and Interpersonal Skills **CT**:Combination therapy **PT:**Psychotherapy **AT:**Attention training **READ:**Reading therapy
